# Supplementary material for: An open-source framework for large-scale, flexible evaluation of biomedical text mining systems
Source: J Biomed Discov Collab. 2008 Jan 29;3:1. doi: 10.1186/1747-5333-3-1 (PMC2276192; doi:10.1186/1747-5333-3-1)
Supplement: Additional file 1 — Evaluation platform source code. The additional file contains the software evaluation framework discussed in this paper. The most current release can be downloaded from SourceForge.net [1]. See the accompanying README file for instructions on installation and use. Also included in the distribution is a collection of UIMA wrappers for some commonly used BioNLP tools and annotated corpora. [file 1747-5333-3-1-S1.zip › bionlp_uima_repository-1.0.1/docs/api/index-all.html]

 
 
 
 
 
 
Index (Center for Computational Pharmacology Util/UIMA API)
 


 

 
function windowTitle()
{
    parent.document.title="Index (Center for Computational Pharmacology Util/UIMA API)";
}
 
 
 

 

 


 
   
  
 
 
 
   
 
   
          Overview     
        Package   
        Class   
          Tree     
          Deprecated     
       Index    
          Help     
   
 
 
  
 
 
 

 
  
 PREV 
 NEXT  
  
    FRAMES     
   NO FRAMES     
  
    All Classes  ');
  }
  //-->
 
 
    All Classes  
 


  
 
 
  
 

 A   B   C   D   E   F   G   H   I   K   L   M   N   O   P   R   S   T   U   V   W   X   Y   
    
 A  
 
   AbGene_AE   - Class in  edu.uchsc.ccp.uima.ae.nlp.ei.gene      AbGene_AE()   - 
Constructor for class edu.uchsc.ccp.uima.ae.nlp.ei.gene. AbGene_AE 
  
   AbGeneEISystem   - Class in  edu.uchsc.ccp.util.external.abgene      AbGeneEISystem(String)   - 
Constructor for class edu.uchsc.ccp.util.external.abgene. AbGeneEISystem 
 Initialize a new AbGene EI System
   ABNER_AE   - Class in  edu.uchsc.ccp.uima.ae.nlp.ei.gene  The class incorporates the ABNER entity identification system into the UIMA analysis engine framework.   ABNER_AE()   - 
Constructor for class edu.uchsc.ccp.uima.ae.nlp.ei.gene. ABNER_AE 
  
   Abner_Util   - Class in  edu.uchsc.ccp.util.external.abner  crap   Abner_Util(int)   - 
Constructor for class edu.uchsc.ccp.util.external.abner. Abner_Util 
 Create a new Abner_Util.
   ABSTRACT_ID   - 
Static variable in interface edu.uchsc.ccp.util.nlp.document. DocumentSectionTypes 
  
   ABSTRACT_NAME   - 
Static variable in interface edu.uchsc.ccp.util.nlp.document. DocumentSectionTypes 
  
   add(PRFResult)   - 
Method in class edu.uchsc.ccp.util.nlp.stats. PRFResult 
 Update this  PRFResult  object by adding the TP, FP, and FN counts from another  PRFResult  object.
   addAnnotation(TextAnnotation)   - 
Method in class edu.uchsc.ccp.util.nlp.document. GenericDocument 
  
   addAnnotationGroup(AnnotationGroupID)   - 
Method in class edu.uchsc.ccp.uima.cc.summary.comparison. ComparisonGroup 
  
   addAnnotationSet(AnnotationSet)   - 
Method in class edu.uchsc.ccp.util.nlp.annotation. TextAnnotation 
 Add an annotation set
   addAnnotationType(String)   - 
Method in class edu.uchsc.ccp.uima.cc.summary.comparison. AnnotationGroup 
  
   addCCPTextAnnotationToCCPClassMention(CCPTextAnnotation, CCPClassMention)   - 
Static method in class edu.uchsc.ccp.uima.util. UIMA_Util 
  
   addClassMention(ClassMention)   - 
Method in class edu.uchsc.ccp.util.nlp.mention. ComplexSlotMention 
 Add a class mention as a slot filler for this complex slot
   addComplexSlotMention(ComplexSlotMention)   - 
Method in class edu.uchsc.ccp.util.nlp.mention. ClassMention 
  
   addDocumentSection(DocumentSection)   - 
Method in class edu.uchsc.ccp.util.nlp.document. GenericDocument 
  
   addFNAnnotation(TextAnnotation)   - 
Method in class edu.uchsc.ccp.util.nlp.stats. PRFResult 
 Add a false negative  TextAnnotation 
   addFPAnnotation(TextAnnotation)   - 
Method in class edu.uchsc.ccp.util.nlp.stats. PRFResult 
 Add a false positive  TextAnnotation 
   addOtherDocumentID(String, String)   - 
Method in class edu.uchsc.ccp.util.nlp.document. GenericDocument 
 Insert a new document ID (other than the primary ID)
   addPatternFilename(String)   - 
Method in class edu.uchsc.ccp.uima.ae.nlp.opendmap. OpenDmapAnnotator 
 Add the name of an OpenDMAP pattern file to be loaded
 into the parser when this annotator is initialized.
   addSecondaryDocumentID(String)   - 
Method in class edu.uchsc.ccp.util.nlp.document. GenericDocument 
  Deprecated.  
   addSlotMention(SlotMention)   - 
Method in class edu.uchsc.ccp.util.nlp.mention. ClassMention 
  
   addSlotValue(CCPClassMention, String, String)   - 
Static method in class edu.uchsc.ccp.uima.util. UIMA_Util 
  
   addSlotValue(Object)   - 
Method in class edu.uchsc.ccp.util.nlp.mention. SlotMention 
 Add a slot value to this slot mention
   addSpan(Span)   - 
Method in class edu.uchsc.ccp.util.nlp.annotation. TextAnnotation 
 Add a span to this TextAnnotation.
   addTextAnnotation(TextAnnotation)   - 
Method in class edu.uchsc.ccp.util.nlp.mention. ClassMention 
  
   addTPAnnotation(TextAnnotation)   - 
Method in class edu.uchsc.ccp.util.nlp.stats. PRFResult 
 Add a true positive  TextAnnotation 
   ALANINE   - 
Static variable in interface edu.uchsc.ccp.util.nlp.mention. ClassMentionTypes 
  
   AMINO_ACID   - 
Static variable in interface edu.uchsc.ccp.util.nlp.mention. ClassMentionTypes 
  
   Annotation_Util   - Class in  edu.uchsc.ccp.util.nlp.annotation      Annotation_Util()   - 
Constructor for class edu.uchsc.ccp.util.nlp.annotation. Annotation_Util 
  
   AnnotationComparator   - Class in  edu.uchsc.ccp.util.nlp.annotation.comparison      AnnotationComparator()   - 
Constructor for class edu.uchsc.ccp.util.nlp.annotation.comparison. AnnotationComparator 
  
   AnnotationComparator_CC   - Class in  edu.uchsc.ccp.uima.cc.summary.comparison      AnnotationComparator_CC()   - 
Constructor for class edu.uchsc.ccp.uima.cc.summary.comparison. AnnotationComparator_CC 
  
   AnnotationConsensusFilter_AE   - Class in  edu.uchsc.ccp.uima.ae.util.filter      AnnotationConsensusFilter_AE()   - 
Constructor for class edu.uchsc.ccp.uima.ae.util.filter. AnnotationConsensusFilter_AE 
  
   AnnotationFromFileLoader_AE   - Class in  edu.uchsc.ccp.uima.ae.util  This AE reads in annotations from a file.   AnnotationFromFileLoader_AE()   - 
Constructor for class edu.uchsc.ccp.uima.ae.util. AnnotationFromFileLoader_AE 
  
   AnnotationGroup   - Class in  edu.uchsc.ccp.uima.cc.summary.comparison      AnnotationGroup()   - 
Constructor for class edu.uchsc.ccp.uima.cc.summary.comparison. AnnotationGroup 
  
   AnnotationGroupID   - Class in  edu.uchsc.ccp.uima.cc.summary.comparison      AnnotationGroupID()   - 
Constructor for class edu.uchsc.ccp.uima.cc.summary.comparison. AnnotationGroupID 
  
   AnnotationGroupID(String)   - 
Constructor for class edu.uchsc.ccp.uima.cc.summary.comparison. AnnotationGroupID 
  
   AnnotationMap   - Class in  edu.uchsc.ccp.uima.util.opendmap  This class loads a translation map for use in moving from
 CAS annotations to OpenDMAP input tokens and from OpenDMAP
 output references to CAS annotations.   AnnotationMap()   - 
Constructor for class edu.uchsc.ccp.uima.util.opendmap. AnnotationMap 
 Create an empty annotation map.
   AnnotationMap(String)   - 
Constructor for class edu.uchsc.ccp.uima.util.opendmap. AnnotationMap 
 Create an annotation map from an XML file.
   AnnotationPrinter   - Class in  edu.uchsc.ccp.uima.cc.summary  This class provides a summary of UIMA processing by outputing the annotations in various formats.   AnnotationPrinter()   - 
Constructor for class edu.uchsc.ccp.uima.cc.summary. AnnotationPrinter 
  
   AnnotationPrinterUtil   - Interface in  edu.uchsc.ccp.util.nlp.annotation.printer      AnnotationSet   - Class in  edu.uchsc.ccp.util.nlp.annotation      AnnotationSet(Integer, String, String)   - 
Constructor for class edu.uchsc.ccp.util.nlp.annotation. AnnotationSet 
  
   AnnotationSetFilter_AE   - Class in  edu.uchsc.ccp.uima.ae.util.filter  removes all annotations not created by a particular annotator   AnnotationSetFilter_AE()   - 
Constructor for class edu.uchsc.ccp.uima.ae.util.filter. AnnotationSetFilter_AE 
  
   AnnotationSetOverrider_AE   - Class in  edu.uchsc.ccp.uima.ae.util.converter  Changes annotation set references unless, the annotation has a set in the annotation sets to ignore   AnnotationSetOverrider_AE()   - 
Constructor for class edu.uchsc.ccp.uima.ae.util.converter. AnnotationSetOverrider_AE 
  
   annotationSetsToIgnore   - 
Variable in class edu.uchsc.ccp.uima.ae.util.converter. AnnotationSetOverrider_AE 
  
   annotationSetsToIgnore   - 
Variable in class edu.uchsc.ccp.uima.ae.util.filter. AnnotationConsensusFilter_AE 
  
   annotationSetsToIgnore   - 
Variable in class edu.uchsc.ccp.uima.ae.util.filter. OverlappingAnnotationResolver_AE 
  
   AnnotationString   - Class in  edu.uchsc.ccp.uima.util.opendmap  This class generates a string describing a JCas CCPTextAnnotation in a standard
 format that can be used for generating and executing regression tests.   AnnotationString(CCPTextAnnotation, String[], String, String)   - 
Constructor for class edu.uchsc.ccp.uima.util.opendmap. AnnotationString 
 Create an object that will print as a standard format string for a JCas annotation.
   AnnotationToFileOutput   - Class in  edu.uchsc.ccp.util.nlp.annotation.printer  This Annotation Printer outputs annotation in a format that can be loaded by the AnnotationFromFileLoader.   AnnotationToFileOutput()   - 
Constructor for class edu.uchsc.ccp.util.nlp.annotation.printer. AnnotationToFileOutput 
  
   annotationTypeOfInterest   - 
Variable in class edu.uchsc.ccp.uima.ae.util.filter. AnnotationConsensusFilter_AE 
  
   annotationTypeOfInterest   - 
Variable in class edu.uchsc.ccp.uima.ae.util.filter. OverlappingAnnotationResolver_AE 
  
   Annotator   - Class in  edu.uchsc.ccp.util.nlp.annotation      Annotator(Integer, String, String, String)   - 
Constructor for class edu.uchsc.ccp.util.nlp.annotation. Annotator 
  
   AnnotatorConverter_AE   - Class in  edu.uchsc.ccp.uima.ae.util.converter  Converts the annotator of an annotation based on its current annotator.   AnnotatorConverter_AE()   - 
Constructor for class edu.uchsc.ccp.uima.ae.util.converter. AnnotatorConverter_AE 
  
   AnnotatorFilter_AE   - Class in  edu.uchsc.ccp.uima.ae.util.filter  removes all annotations not created by a particular annotator   AnnotatorFilter_AE()   - 
Constructor for class edu.uchsc.ccp.uima.ae.util.filter. AnnotatorFilter_AE 
  
   AnnotatorOverrider_AE   - Class in  edu.uchsc.ccp.uima.ae.util.converter      AnnotatorOverrider_AE()   - 
Constructor for class edu.uchsc.ccp.uima.ae.util.converter. AnnotatorOverrider_AE 
  
   ARGININE   - 
Static variable in interface edu.uchsc.ccp.util.nlp.mention. ClassMentionTypes 
  
   ASPARAGINE   - 
Static variable in interface edu.uchsc.ccp.util.nlp.mention. ClassMentionTypes 
  
   ASPARTATE   - 
Static variable in interface edu.uchsc.ccp.util.nlp.mention. ClassMentionTypes 
  
 
 
    
 B  
 
   Bio1CorpusParser   - Class in  edu.uchsc.ccp.util.nlp.parser.corpus  A parser for the Bio1 corpus.   Bio1CorpusParser(String)   - 
Constructor for class edu.uchsc.ccp.util.nlp.parser.corpus. Bio1CorpusParser 
  
   BioIECorpusParser   - Class in  edu.uchsc.ccp.util.nlp.parser.corpus  Parser for the BioIE corpus.   BioIECorpusParser(String)   - 
Constructor for class edu.uchsc.ccp.util.nlp.parser.corpus. BioIECorpusParser 
 The corpus directory must contain the source files and word-freak annotation files
   BIOLOGICAL_SEQUENCE   - 
Static variable in interface edu.uchsc.ccp.util.nlp.mention. ClassMentionTypes 
  
   BIOLOGICAL_SEQUENCE_ELEMENT   - 
Static variable in interface edu.uchsc.ccp.util.nlp.mention. ClassMentionTypes 
  
   BIOLOGICAL_SEQUENCE_ELEMENT_POSITION_IN_SEQUENCE   - 
Static variable in interface edu.uchsc.ccp.util.nlp.mention. ComplexSlotMentionTypes 
  
   BIOLOGICAL_SEQUENCE_POSITION   - 
Static variable in interface edu.uchsc.ccp.util.nlp.mention. ClassMentionTypes 
  
   BIOLOGICAL_SEQUENCE_SEQUENCE   - 
Static variable in interface edu.uchsc.ccp.util.nlp.mention. SlotMentionTypes 
 for Mutation project
   BIOLOGICAL_SUBSEQUENCE   - 
Static variable in interface edu.uchsc.ccp.util.nlp.mention. ClassMentionTypes 
  
   BIOLOGICAL_SUBSEQUENCE_END_ELEMENT   - 
Static variable in interface edu.uchsc.ccp.util.nlp.mention. ComplexSlotMentionTypes 
  
   BIOLOGICAL_SUBSEQUENCE_START_ELEMENT   - 
Static variable in interface edu.uchsc.ccp.util.nlp.mention. ComplexSlotMentionTypes 
  
 
 
    
 C  
 
   calculateScore(Reference)   - 
Static method in class edu.uchsc.ccp.uima.util.opendmap. ReferenceProcessor 
 Score the reference.
   CCPAnnotation   - Class in  edu.uchsc.ccp.uima.annotation  The superclass for all CCP annotations.   CCPAnnotation()   - 
Constructor for class edu.uchsc.ccp.uima.annotation. CCPAnnotation 
 Never called.
   CCPAnnotation(int, TOP_Type)   - 
Constructor for class edu.uchsc.ccp.uima.annotation. CCPAnnotation 
 Internal - constructor used by generator
   CCPAnnotation(JCas)   - 
Constructor for class edu.uchsc.ccp.uima.annotation. CCPAnnotation 
  
   CCPAnnotation(JCas, int, int)   - 
Constructor for class edu.uchsc.ccp.uima.annotation. CCPAnnotation 
  
   CCPAnnotation_Type   - Class in  edu.uchsc.ccp.uima.annotation  The superclass for all CCP annotations.   CCPAnnotation_Type(JCas, Type)   - 
Constructor for class edu.uchsc.ccp.uima.annotation. CCPAnnotation_Type 
 initialize variables to correspond with Cas Type and Features
   CCPAnnotationSet   - Class in  edu.uchsc.ccp.uima.annotation  The annotation set provides a means for arbitrarily categorizing or clustering groups of annotations.   CCPAnnotationSet()   - 
Constructor for class edu.uchsc.ccp.uima.annotation. CCPAnnotationSet 
 Never called.
   CCPAnnotationSet(int, TOP_Type)   - 
Constructor for class edu.uchsc.ccp.uima.annotation. CCPAnnotationSet 
 Internal - constructor used by generator
   CCPAnnotationSet(JCas)   - 
Constructor for class edu.uchsc.ccp.uima.annotation. CCPAnnotationSet 
  
   CCPAnnotationSet_Type   - Class in  edu.uchsc.ccp.uima.annotation  The annotation set provides a means for arbitrarily categorizing or clustering groups of annotations.   CCPAnnotationSet_Type(JCas, Type)   - 
Constructor for class edu.uchsc.ccp.uima.annotation. CCPAnnotationSet_Type 
 initialize variables to correspond with Cas Type and Features
   CCPAnnotator   - Class in  edu.uchsc.ccp.uima.annotation  The annotator object contains information which is used to determine who/what generated an annotation.   CCPAnnotator()   - 
Constructor for class edu.uchsc.ccp.uima.annotation. CCPAnnotator 
 Never called.
   CCPAnnotator(int, TOP_Type)   - 
Constructor for class edu.uchsc.ccp.uima.annotation. CCPAnnotator 
 Internal - constructor used by generator
   CCPAnnotator(JCas)   - 
Constructor for class edu.uchsc.ccp.uima.annotation. CCPAnnotator 
  
   CCPAnnotator_Type   - Class in  edu.uchsc.ccp.uima.annotation  The annotator object contains information which is used to determine who/what generated an annotation.   CCPAnnotator_Type(JCas, Type)   - 
Constructor for class edu.uchsc.ccp.uima.annotation. CCPAnnotator_Type 
 initialize variables to correspond with Cas Type and Features
   CCPClassMention   - Class in  edu.uchsc.ccp.uima.mention  The CCP ClassMention is the root of a flexible class structure that can be used to store virtually any frame-based representation of a particular class.   CCPClassMention()   - 
Constructor for class edu.uchsc.ccp.uima.mention. CCPClassMention 
 Never called.
   CCPClassMention(int, TOP_Type)   - 
Constructor for class edu.uchsc.ccp.uima.mention. CCPClassMention 
 Internal - constructor used by generator
   CCPClassMention(JCas)   - 
Constructor for class edu.uchsc.ccp.uima.mention. CCPClassMention 
  
   CCPClassMention_Type   - Class in  edu.uchsc.ccp.uima.mention  The CCP ClassMention is the root of a flexible class structure that can be used to store virtually any frame-based representation of a particular class.   CCPClassMention_Type(JCas, Type)   - 
Constructor for class edu.uchsc.ccp.uima.mention. CCPClassMention_Type 
 initialize variables to correspond with Cas Type and Features
   CCPClauseAnnotation   - Class in  edu.uchsc.ccp.uima.annotation.syntactic  This annotation type is used to annotation clauses.   CCPClauseAnnotation()   - 
Constructor for class edu.uchsc.ccp.uima.annotation.syntactic. CCPClauseAnnotation 
 Never called.
   CCPClauseAnnotation(int, TOP_Type)   - 
Constructor for class edu.uchsc.ccp.uima.annotation.syntactic. CCPClauseAnnotation 
 Internal - constructor used by generator
   CCPClauseAnnotation(JCas)   - 
Constructor for class edu.uchsc.ccp.uima.annotation.syntactic. CCPClauseAnnotation 
  
   CCPClauseAnnotation(JCas, int, int)   - 
Constructor for class edu.uchsc.ccp.uima.annotation.syntactic. CCPClauseAnnotation 
  
   CCPClauseAnnotation_Type   - Class in  edu.uchsc.ccp.uima.annotation.syntactic  This annotation type is used to annotation clauses.   CCPClauseAnnotation_Type(JCas, Type)   - 
Constructor for class edu.uchsc.ccp.uima.annotation.syntactic. CCPClauseAnnotation_Type 
 initialize variables to correspond with Cas Type and Features
   CCPComplexSlotMention   - Class in  edu.uchsc.ccp.uima.mention  A slot mention is deemed "complex" when its slot filler is a class mention as opposed to a String (See non-complex slot mention for String fillers).   CCPComplexSlotMention()   - 
Constructor for class edu.uchsc.ccp.uima.mention. CCPComplexSlotMention 
 Never called.
   CCPComplexSlotMention(int, TOP_Type)   - 
Constructor for class edu.uchsc.ccp.uima.mention. CCPComplexSlotMention 
 Internal - constructor used by generator
   CCPComplexSlotMention(JCas)   - 
Constructor for class edu.uchsc.ccp.uima.mention. CCPComplexSlotMention 
  
   CCPComplexSlotMention_Type   - Class in  edu.uchsc.ccp.uima.mention  A slot mention is deemed "complex" when its slot filler is a class mention as opposed to a String (See non-complex slot mention for String fillers).   CCPComplexSlotMention_Type(JCas, Type)   - 
Constructor for class edu.uchsc.ccp.uima.mention. CCPComplexSlotMention_Type 
 initialize variables to correspond with Cas Type and Features
   CCPDocumentAnnotation   - Class in  edu.uchsc.ccp.uima.annotation  Superclass for all document level annotations.   CCPDocumentAnnotation()   - 
Constructor for class edu.uchsc.ccp.uima.annotation. CCPDocumentAnnotation 
 Never called.
   CCPDocumentAnnotation(int, TOP_Type)   - 
Constructor for class edu.uchsc.ccp.uima.annotation. CCPDocumentAnnotation 
 Internal - constructor used by generator
   CCPDocumentAnnotation(JCas)   - 
Constructor for class edu.uchsc.ccp.uima.annotation. CCPDocumentAnnotation 
  
   CCPDocumentAnnotation(JCas, int, int)   - 
Constructor for class edu.uchsc.ccp.uima.annotation. CCPDocumentAnnotation 
  
   CCPDocumentAnnotation_Type   - Class in  edu.uchsc.ccp.uima.annotation  Superclass for all document level annotations.   CCPDocumentAnnotation_Type(JCas, Type)   - 
Constructor for class edu.uchsc.ccp.uima.annotation. CCPDocumentAnnotation_Type 
 initialize variables to correspond with Cas Type and Features
   CCPDocumentInformation   - Class in  edu.uchsc.ccp.uima.annotation  The CCPDocumentInformation annotation includes document metadata such as the document ID, document collection ID, secondary document IDs, document size, etc.   CCPDocumentInformation()   - 
Constructor for class edu.uchsc.ccp.uima.annotation. CCPDocumentInformation 
 Never called.
   CCPDocumentInformation(int, TOP_Type)   - 
Constructor for class edu.uchsc.ccp.uima.annotation. CCPDocumentInformation 
 Internal - constructor used by generator
   CCPDocumentInformation(JCas)   - 
Constructor for class edu.uchsc.ccp.uima.annotation. CCPDocumentInformation 
  
   CCPDocumentInformation(JCas, int, int)   - 
Constructor for class edu.uchsc.ccp.uima.annotation. CCPDocumentInformation 
  
   CCPDocumentInformation_Type   - Class in  edu.uchsc.ccp.uima.annotation  The CCPDocumentInformation annotation includes document metadata such as the document ID, document collection ID, secondary document IDs, document size, etc.   CCPDocumentInformation_Type(JCas, Type)   - 
Constructor for class edu.uchsc.ccp.uima.annotation. CCPDocumentInformation_Type 
 initialize variables to correspond with Cas Type and Features
   CCPDocumentSection   - Class in  edu.uchsc.ccp.uima.annotation  The Document Section annotation allows for an entire document section to be annotated as such, and stores section-specific information such as the section identifier.   CCPDocumentSection()   - 
Constructor for class edu.uchsc.ccp.uima.annotation. CCPDocumentSection 
 Never called.
   CCPDocumentSection(int, TOP_Type)   - 
Constructor for class edu.uchsc.ccp.uima.annotation. CCPDocumentSection 
 Internal - constructor used by generator
   CCPDocumentSection(JCas)   - 
Constructor for class edu.uchsc.ccp.uima.annotation. CCPDocumentSection 
  
   CCPDocumentSection(JCas, int, int)   - 
Constructor for class edu.uchsc.ccp.uima.annotation. CCPDocumentSection 
  
   CCPDocumentSection_Type   - Class in  edu.uchsc.ccp.uima.annotation  The Document Section annotation allows for an entire document section to be annotated as such, and stores section-specific information such as the section identifier.   CCPDocumentSection_Type(JCas, Type)   - 
Constructor for class edu.uchsc.ccp.uima.annotation. CCPDocumentSection_Type 
 initialize variables to correspond with Cas Type and Features
   CCPDocumentSubSection   - Class in  edu.uchsc.ccp.uima.annotation  This annotation type is used to annotate document subsections.   CCPDocumentSubSection()   - 
Constructor for class edu.uchsc.ccp.uima.annotation. CCPDocumentSubSection 
 Never called.
   CCPDocumentSubSection(int, TOP_Type)   - 
Constructor for class edu.uchsc.ccp.uima.annotation. CCPDocumentSubSection 
 Internal - constructor used by generator
   CCPDocumentSubSection(JCas)   - 
Constructor for class edu.uchsc.ccp.uima.annotation. CCPDocumentSubSection 
  
   CCPDocumentSubSection(JCas, int, int)   - 
Constructor for class edu.uchsc.ccp.uima.annotation. CCPDocumentSubSection 
  
   CCPDocumentSubSection_Type   - Class in  edu.uchsc.ccp.uima.annotation  This annotation type is used to annotate document subsections.   CCPDocumentSubSection_Type(JCas, Type)   - 
Constructor for class edu.uchsc.ccp.uima.annotation. CCPDocumentSubSection_Type 
 initialize variables to correspond with Cas Type and Features
   CCPMention   - Class in  edu.uchsc.ccp.uima.mention  The superclass for all CCP Mentions (class mention, complex slot mention, and non-complex slot mention)
 Updated by JCasGen Tue Aug 28 16:44:10 MDT 2007
 XML source: /home/williamb/eclipse/RELEASED_SOURCE_CODE/descriptors/typeSystem/CCPTypeSystem.xml   CCPMention()   - 
Constructor for class edu.uchsc.ccp.uima.mention. CCPMention 
 Never called.
   CCPMention(int, TOP_Type)   - 
Constructor for class edu.uchsc.ccp.uima.mention. CCPMention 
 Internal - constructor used by generator
   CCPMention(JCas)   - 
Constructor for class edu.uchsc.ccp.uima.mention. CCPMention 
  
   CCPMention_Type   - Class in  edu.uchsc.ccp.uima.mention  The superclass for all CCP Mentions (class mention, complex slot mention, and non-complex slot mention)
 Updated by JCasGen Tue Aug 28 16:44:10 MDT 2007   CCPMention_Type(JCas, Type)   - 
Constructor for class edu.uchsc.ccp.uima.mention. CCPMention_Type 
 initialize variables to correspond with Cas Type and Features
   CCPNonComplexSlotMention   - Class in  edu.uchsc.ccp.uima.mention  The non-complex slot mention has slot values which are constrained to be Strings.   CCPNonComplexSlotMention()   - 
Constructor for class edu.uchsc.ccp.uima.mention. CCPNonComplexSlotMention 
 Never called.
   CCPNonComplexSlotMention(int, TOP_Type)   - 
Constructor for class edu.uchsc.ccp.uima.mention. CCPNonComplexSlotMention 
 Internal - constructor used by generator
   CCPNonComplexSlotMention(JCas)   - 
Constructor for class edu.uchsc.ccp.uima.mention. CCPNonComplexSlotMention 
  
   CCPNonComplexSlotMention_Type   - Class in  edu.uchsc.ccp.uima.mention  The non-complex slot mention has slot values which are constrained to be Strings.   CCPNonComplexSlotMention_Type(JCas, Type)   - 
Constructor for class edu.uchsc.ccp.uima.mention. CCPNonComplexSlotMention_Type 
 initialize variables to correspond with Cas Type and Features
   CCPOpenDMAP_AE   - Class in  edu.uchsc.ccp.uima.ae.nlp.opendmap  This class packages OpenDMAP into the UIMA framework.   CCPOpenDMAP_AE()   - 
Constructor for class edu.uchsc.ccp.uima.ae.nlp.opendmap. CCPOpenDMAP_AE 
 Create a new GeneRIF Annotator that uses OpenDMAP to
 process the input document text.
   CCPPhraseAnnotation   - Class in  edu.uchsc.ccp.uima.annotation.syntactic  This annotation type is used to annotation phrases.   CCPPhraseAnnotation()   - 
Constructor for class edu.uchsc.ccp.uima.annotation.syntactic. CCPPhraseAnnotation 
 Never called.
   CCPPhraseAnnotation(int, TOP_Type)   - 
Constructor for class edu.uchsc.ccp.uima.annotation.syntactic. CCPPhraseAnnotation 
 Internal - constructor used by generator
   CCPPhraseAnnotation(JCas)   - 
Constructor for class edu.uchsc.ccp.uima.annotation.syntactic. CCPPhraseAnnotation 
  
   CCPPhraseAnnotation(JCas, int, int)   - 
Constructor for class edu.uchsc.ccp.uima.annotation.syntactic. CCPPhraseAnnotation 
  
   CCPPhraseAnnotation_Type   - Class in  edu.uchsc.ccp.uima.annotation.syntactic  This annotation type is used to annotation phrases.   CCPPhraseAnnotation_Type(JCas, Type)   - 
Constructor for class edu.uchsc.ccp.uima.annotation.syntactic. CCPPhraseAnnotation_Type 
 initialize variables to correspond with Cas Type and Features
   CCPSemanticAnnotation   - Class in  edu.uchsc.ccp.uima.annotation  Superclass for all semantic (i.e.   CCPSemanticAnnotation()   - 
Constructor for class edu.uchsc.ccp.uima.annotation. CCPSemanticAnnotation 
 Never called.
   CCPSemanticAnnotation(int, TOP_Type)   - 
Constructor for class edu.uchsc.ccp.uima.annotation. CCPSemanticAnnotation 
 Internal - constructor used by generator
   CCPSemanticAnnotation(JCas)   - 
Constructor for class edu.uchsc.ccp.uima.annotation. CCPSemanticAnnotation 
  
   CCPSemanticAnnotation(JCas, int, int)   - 
Constructor for class edu.uchsc.ccp.uima.annotation. CCPSemanticAnnotation 
  
   CCPSemanticAnnotation_Type   - Class in  edu.uchsc.ccp.uima.annotation  Superclass for all semantic (i.e.   CCPSemanticAnnotation_Type(JCas, Type)   - 
Constructor for class edu.uchsc.ccp.uima.annotation. CCPSemanticAnnotation_Type 
 initialize variables to correspond with Cas Type and Features
   CCPSentenceAnnotation   - Class in  edu.uchsc.ccp.uima.annotation.syntactic  This annotation type is used to annotate sentences within the document text.   CCPSentenceAnnotation()   - 
Constructor for class edu.uchsc.ccp.uima.annotation.syntactic. CCPSentenceAnnotation 
 Never called.
   CCPSentenceAnnotation(int, TOP_Type)   - 
Constructor for class edu.uchsc.ccp.uima.annotation.syntactic. CCPSentenceAnnotation 
 Internal - constructor used by generator
   CCPSentenceAnnotation(JCas)   - 
Constructor for class edu.uchsc.ccp.uima.annotation.syntactic. CCPSentenceAnnotation 
  
   CCPSentenceAnnotation(JCas, int, int)   - 
Constructor for class edu.uchsc.ccp.uima.annotation.syntactic. CCPSentenceAnnotation 
  
   CCPSentenceAnnotation_Type   - Class in  edu.uchsc.ccp.uima.annotation.syntactic  This annotation type is used to annotate sentences within the document text.   CCPSentenceAnnotation_Type(JCas, Type)   - 
Constructor for class edu.uchsc.ccp.uima.annotation.syntactic. CCPSentenceAnnotation_Type 
 initialize variables to correspond with Cas Type and Features
   CCPSlotMention   - Class in  edu.uchsc.ccp.uima.mention  The superclass for all slot mentions (complex and non-complex).   CCPSlotMention()   - 
Constructor for class edu.uchsc.ccp.uima.mention. CCPSlotMention 
 Never called.
   CCPSlotMention(int, TOP_Type)   - 
Constructor for class edu.uchsc.ccp.uima.mention. CCPSlotMention 
 Internal - constructor used by generator
   CCPSlotMention(JCas)   - 
Constructor for class edu.uchsc.ccp.uima.mention. CCPSlotMention 
  
   CCPSlotMention_Type   - Class in  edu.uchsc.ccp.uima.mention  The superclass for all slot mentions (complex and non-complex).   CCPSlotMention_Type(JCas, Type)   - 
Constructor for class edu.uchsc.ccp.uima.mention. CCPSlotMention_Type 
 initialize variables to correspond with Cas Type and Features
   CCPSpan   - Class in  edu.uchsc.ccp.uima.annotation  The span object holds span information.This is a supplement to the default UIMA annotation which cannot handle multi-span annotations.   CCPSpan()   - 
Constructor for class edu.uchsc.ccp.uima.annotation. CCPSpan 
 Never called.
   CCPSpan(int, TOP_Type)   - 
Constructor for class edu.uchsc.ccp.uima.annotation. CCPSpan 
 Internal - constructor used by generator
   CCPSpan(JCas)   - 
Constructor for class edu.uchsc.ccp.uima.annotation. CCPSpan 
  
   CCPSpan_Type   - Class in  edu.uchsc.ccp.uima.annotation  The span object holds span information.This is a supplement to the default UIMA annotation which cannot handle multi-span annotations.   CCPSpan_Type(JCas, Type)   - 
Constructor for class edu.uchsc.ccp.uima.annotation. CCPSpan_Type 
 initialize variables to correspond with Cas Type and Features
   CCPSyntacticAnnotation   - Class in  edu.uchsc.ccp.uima.annotation  Super class for all syntactic annotations.   CCPSyntacticAnnotation()   - 
Constructor for class edu.uchsc.ccp.uima.annotation. CCPSyntacticAnnotation 
 Never called.
   CCPSyntacticAnnotation(int, TOP_Type)   - 
Constructor for class edu.uchsc.ccp.uima.annotation. CCPSyntacticAnnotation 
 Internal - constructor used by generator
   CCPSyntacticAnnotation(JCas)   - 
Constructor for class edu.uchsc.ccp.uima.annotation. CCPSyntacticAnnotation 
  
   CCPSyntacticAnnotation(JCas, int, int)   - 
Constructor for class edu.uchsc.ccp.uima.annotation. CCPSyntacticAnnotation 
  
   CCPSyntacticAnnotation_Type   - Class in  edu.uchsc.ccp.uima.annotation  Super class for all syntactic annotations.   CCPSyntacticAnnotation_Type(JCas, Type)   - 
Constructor for class edu.uchsc.ccp.uima.annotation. CCPSyntacticAnnotation_Type 
 initialize variables to correspond with Cas Type and Features
   CCPTextAnnotation   - Class in  edu.uchsc.ccp.uima.annotation  The CCP TextAnnotation extends the base annotation class to include an annotation ID, the capability for multiple annotation spans, a link to the annotator responsible for generating the annotation, membership to annotation sets, and a link to a class mention which defines the class of this annotation.   CCPTextAnnotation()   - 
Constructor for class edu.uchsc.ccp.uima.annotation. CCPTextAnnotation 
 Never called.
   CCPTextAnnotation(int, TOP_Type)   - 
Constructor for class edu.uchsc.ccp.uima.annotation. CCPTextAnnotation 
 Internal - constructor used by generator
   CCPTextAnnotation(JCas)   - 
Constructor for class edu.uchsc.ccp.uima.annotation. CCPTextAnnotation 
  
   CCPTextAnnotation(JCas, int, int)   - 
Constructor for class edu.uchsc.ccp.uima.annotation. CCPTextAnnotation 
  
   CCPTextAnnotation_Type   - Class in  edu.uchsc.ccp.uima.annotation  The CCP TextAnnotation extends the base annotation class to include an annotation ID, the capability for multiple annotation spans, a link to the annotator responsible for generating the annotation, membership to annotation sets, and a link to a class mention which defines the class of this annotation.   CCPTextAnnotation_Type(JCas, Type)   - 
Constructor for class edu.uchsc.ccp.uima.annotation. CCPTextAnnotation_Type 
 initialize variables to correspond with Cas Type and Features
   CCPTokenAnnotation   - Class in  edu.uchsc.ccp.uima.annotation.syntactic  This annotation type is used to mark up tokens.   CCPTokenAnnotation()   - 
Constructor for class edu.uchsc.ccp.uima.annotation.syntactic. CCPTokenAnnotation 
 Never called.
   CCPTokenAnnotation(int, TOP_Type)   - 
Constructor for class edu.uchsc.ccp.uima.annotation.syntactic. CCPTokenAnnotation 
 Internal - constructor used by generator
   CCPTokenAnnotation(JCas)   - 
Constructor for class edu.uchsc.ccp.uima.annotation.syntactic. CCPTokenAnnotation 
  
   CCPTokenAnnotation(JCas, int, int)   - 
Constructor for class edu.uchsc.ccp.uima.annotation.syntactic. CCPTokenAnnotation 
  
   CCPTokenAnnotation_Type   - Class in  edu.uchsc.ccp.uima.annotation.syntactic  This annotation type is used to mark up tokens.   CCPTokenAnnotation_Type(JCas, Type)   - 
Constructor for class edu.uchsc.ccp.uima.annotation.syntactic. CCPTokenAnnotation_Type 
 initialize variables to correspond with Cas Type and Features
   CCPTypedDependency   - Class in  edu.uchsc.ccp.uima.annotation.syntactic  This typed dependency structure was created to hold typed dependency information generated by the Stanford Parser.   CCPTypedDependency()   - 
Constructor for class edu.uchsc.ccp.uima.annotation.syntactic. CCPTypedDependency 
 Never called.
   CCPTypedDependency(int, TOP_Type)   - 
Constructor for class edu.uchsc.ccp.uima.annotation.syntactic. CCPTypedDependency 
 Internal - constructor used by generator
   CCPTypedDependency(JCas)   - 
Constructor for class edu.uchsc.ccp.uima.annotation.syntactic. CCPTypedDependency 
  
   CCPTypedDependency_Type   - Class in  edu.uchsc.ccp.uima.annotation.syntactic  This typed dependency structure was created to hold typed dependency information generated by the Stanford Parser.   CCPTypedDependency_Type(JCas, Type)   - 
Constructor for class edu.uchsc.ccp.uima.annotation.syntactic. CCPTypedDependency_Type 
 initialize variables to correspond with Cas Type and Features
   CELL_LINE   - 
Static variable in interface edu.uchsc.ccp.util.nlp.mention. ClassMentionTypes 
  
   CELL_TYPE   - 
Static variable in interface edu.uchsc.ccp.util.nlp.mention. ClassMentionTypes 
  
   checkForIgnoreBasedOnAnnotationSet(CCPTextAnnotation)   - 
Method in class edu.uchsc.ccp.uima.ae.util.converter. AnnotationSetOverrider_AE 
  
   checkForIgnoreBasedOnAnnotationSet(CCPTextAnnotation)   - 
Method in class edu.uchsc.ccp.uima.ae.util.filter. AnnotationConsensusFilter_AE 
  
   checkForIgnoreBasedOnAnnotationSet(CCPTextAnnotation)   - 
Method in class edu.uchsc.ccp.uima.ae.util.filter. OverlappingAnnotationResolver_AE 
  
   CLASS_MENTION_TYPE   - 
Static variable in interface edu.uchsc.ccp.util.nlp.mention. ClassMentionTypes 
 ClassMention Identifier - if this changes, Annotation DB must be adjusted accordingly
   ClassMention   - Class in  edu.uchsc.ccp.util.nlp.mention      ClassMention()   - 
Constructor for class edu.uchsc.ccp.util.nlp.mention. ClassMention 
  Deprecated.  
   ClassMention(String)   - 
Constructor for class edu.uchsc.ccp.util.nlp.mention. ClassMention 
  
   ClassMentionComparator   - Interface in  edu.uchsc.ccp.util.nlp.mention.comparison      ClassMentionConverter_AE   - Class in  edu.uchsc.ccp.uima.ae.util.converter  Converts the class mentions of the user supplied types to the target mantion type   ClassMentionConverter_AE()   - 
Constructor for class edu.uchsc.ccp.uima.ae.util.converter. ClassMentionConverter_AE 
  
   ClassMentionRemovalFilter_AE   - Class in  edu.uchsc.ccp.uima.ae.util.filter  removes all annotations that are not a specific class mention from the CAS indexes   ClassMentionRemovalFilter_AE()   - 
Constructor for class edu.uchsc.ccp.uima.ae.util.filter. ClassMentionRemovalFilter_AE 
  
   ClassMentionTypeFilter_AE   - Class in  edu.uchsc.ccp.uima.ae.util.filter  removes all annotations that are not a specific class mention   ClassMentionTypeFilter_AE()   - 
Constructor for class edu.uchsc.ccp.uima.ae.util.filter. ClassMentionTypeFilter_AE 
  
   ClassMentionTypes   - Interface in  edu.uchsc.ccp.util.nlp.mention      CLAUSE   - 
Static variable in interface edu.uchsc.ccp.util.nlp.mention. ClassMentionTypes 
  
   CLAUSE_LEVEL   - 
Static variable in interface edu.uchsc.ccp.util.nlp.mention. SlotMentionTypes 
  
   CLAUSE_NUMBER   - 
Static variable in interface edu.uchsc.ccp.util.nlp.mention. SlotMentionTypes 
  
   CLAUSE_TYPE   - 
Static variable in interface edu.uchsc.ccp.util.nlp.mention. SlotMentionTypes 
  
   clone()   - 
Method in class edu.uchsc.ccp.uima.util.opendmap. DMAPStanfordPhraseGraph 
 Copy this property function handler and any internal record functions.
   clone()   - 
Method in class edu.uchsc.ccp.util.nlp.annotation. Span 
  
   close()   - 
Method in class edu.uchsc.ccp.util.nlp.parser.corpus. Bio1CorpusParser 
  
   close()   - 
Method in class edu.uchsc.ccp.util.nlp.parser.corpus. BioIECorpusParser 
  
   close()   - 
Method in class edu.uchsc.ccp.util.nlp.parser.corpus. CorpusParser 
  
   close()   - 
Method in class edu.uchsc.ccp.util.nlp.parser.corpus. GeniaCorpusParser 
  
   close()   - 
Method in class edu.uchsc.ccp.util.nlp.parser.corpus. IProLinkCorpus1Parser 
  
   close()   - 
Method in class edu.uchsc.ccp.util.nlp.parser.corpus. IProLinkCorpus2Parser 
  
   close()   - 
Method in class edu.uchsc.ccp.util.nlp.parser.corpus. TexasCorpusParser 
  
   close()   - 
Method in class edu.uchsc.ccp.util.nlp.parser.corpus. YapexCorpusParser 
  
   collectionProcessComplete(ProcessTrace)   - 
Method in class edu.uchsc.ccp.uima.cc.summary. AnnotationPrinter 
 Closes the PrintStream, if one was used by the program.
   collectionProcessComplete(ProcessTrace)   - 
Method in class edu.uchsc.ccp.uima.cc.summary.comparison. AnnotationComparator_CC 
 Print the comparison results
   ComparatorConfigurator   - Class in  edu.uchsc.ccp.uima.cc.summary.comparison  This is a utility class for configuring an AnnotationComparator.   ComparatorConfigurator(String)   - 
Constructor for class edu.uchsc.ccp.uima.cc.summary.comparison. ComparatorConfigurator 
  
   compare(TextAnnotation, TextAnnotation)   - 
Method in class edu.uchsc.ccp.util.nlp.annotation.comparison. AnnotationComparator 
 Default TextAnnotation comparison utilizes the StrictSpanComparator and the IdenticalMentionComparator family
   compare(TextAnnotation, TextAnnotation, SpanComparator, ClassMentionComparator)   - 
Method in class edu.uchsc.ccp.util.nlp.annotation.comparison. AnnotationComparator 
 Compares two TextAnnotations using the supplied SpanComparator and ClassMentionComparator
   compare(List<TextAnnotation>, List<TextAnnotation>)   - 
Method in class edu.uchsc.ccp.util.nlp.annotation.comparison. AnnotationComparator 
 Default comparison of two lists of TextAnnotations utilizing the StrictSpanComparator and the IdenticalMentionComparator family
   compare(List<TextAnnotation>, List<TextAnnotation>, SpanComparator, ClassMentionComparator)   - 
Method in class edu.uchsc.ccp.util.nlp.annotation.comparison. AnnotationComparator 
 Compares two lists of TextAnnotations using the supplied SpanComparator and ClassMentionComparator
   compare(TextAnnotation, TextAnnotation, SpanComparator, ClassMentionComparator, int)   - 
Method in class edu.uchsc.ccp.util.nlp.annotation.comparison. AnnotationComparator 
 Compares two TextAnnotations using the supplied SpanComparator and ClassMentionComparator, but only compares to the supplied comparison depth.
   compare(List<TextAnnotation>, List<TextAnnotation>, SpanComparator, ClassMentionComparator, int)   - 
Method in class edu.uchsc.ccp.util.nlp.annotation.comparison. AnnotationComparator 
 Compares two lists of TextAnnotations using the supplied SpanComparator and ClassMentionComparator, but only compares to the supplied
 comparison depth.
   compare(List<TextAnnotation>, List<TextAnnotation>)   - 
Method in class edu.uchsc.ccp.util.nlp.annotation.comparison. EntityPartsAnnotationComparator 
  
   compare(TextAnnotation, TextAnnotation)   - 
Method in class edu.uchsc.ccp.util.nlp.annotation.comparison. EntityPartsAnnotationComparator 
 Since this method returns an integer, it does not make sense to use it to compare the entity parts, however, in order to override the default
 compare() method of AnnotationComparator which employs the strict span comparator, we will implement this method to return 0 if the resulting F
 measure is 1.0, -1 otherwise.
   compare(List<TextAnnotation>, List<TextAnnotation>, SpanComparator, ClassMentionComparator, int)   - 
Method in class edu.uchsc.ccp.util.nlp.annotation.comparison. EntityPartsAnnotationComparator 
 Overridden - note this ignores all extraneous input parameters and simple returns EntityPartsAnnotationComparator.compare(List 
 goldTaList, List  testTaList)
   compare(List<TextAnnotation>, List<TextAnnotation>, SpanComparator, ClassMentionComparator)   - 
Method in class edu.uchsc.ccp.util.nlp.annotation.comparison. EntityPartsAnnotationComparator 
 Overridden - note this ignores all extraneous input parameters and simple returns EntityPartsAnnotationComparator.compare(List 
 goldTaList, List  testTaList)
   compare(TextAnnotation, TextAnnotation, SpanComparator, ClassMentionComparator, int)   - 
Method in class edu.uchsc.ccp.util.nlp.annotation.comparison. EntityPartsAnnotationComparator 
 Overridden - note this ignores all extraneous input parameters and simple returns EntityPartsAnnotationComparator.compare(TextAnnotation ta1,
 TextAnnotation ta2)
   compare(TextAnnotation, TextAnnotation, SpanComparator, ClassMentionComparator)   - 
Method in class edu.uchsc.ccp.util.nlp.annotation.comparison. EntityPartsAnnotationComparator 
 Overridden - note this ignores all extraneous input parameters and simple returns EntityPartsAnnotationComparator.compare(TextAnnotation ta1,
 TextAnnotation ta2)
   compare(Span, Span)   - 
Method in class edu.uchsc.ccp.util.nlp.annotation.comparison. IgnoreSpanComparator 
 Ignores the spans and returns 0 always.
   compare(List<Span>, List<Span>)   - 
Method in class edu.uchsc.ccp.util.nlp.annotation.comparison. IgnoreSpanComparator 
 Ignores the span lists and returns 0 always.
   compare(Span, Span)   - 
Method in class edu.uchsc.ccp.util.nlp.annotation.comparison. SharedEndSpanComparator 
 Compare two spans to see if they share the same end.
   compare(List<Span>, List<Span>)   - 
Method in class edu.uchsc.ccp.util.nlp.annotation.comparison. SharedEndSpanComparator 
 Compare two lists of spans using the shared-end match criteria.
   compare(Span, Span)   - 
Method in class edu.uchsc.ccp.util.nlp.annotation.comparison. SharedStartOrEndSpanComparator 
 Compare two spans using the shares-span-start-or-end match criteria.
   compare(List<Span>, List<Span>)   - 
Method in class edu.uchsc.ccp.util.nlp.annotation.comparison. SharedStartOrEndSpanComparator 
 Compare two lists of spans using the shares-span-start-or-end match criteria.
   compare(Span, Span)   - 
Method in class edu.uchsc.ccp.util.nlp.annotation.comparison. SharedStartSpanComparator 
 Compare two spans using the shares-span-start match criteria.
   compare(List<Span>, List<Span>)   - 
Method in class edu.uchsc.ccp.util.nlp.annotation.comparison. SharedStartSpanComparator 
 Compare two lists of spans using the shares-span-start match criteria.
   compare(Span, Span)   - 
Method in class edu.uchsc.ccp.util.nlp.annotation.comparison. SloppySpanComparator 
 Compare two spans using the sloppy (overlap) match criteria.
   compare(List<Span>, List<Span>)   - 
Method in class edu.uchsc.ccp.util.nlp.annotation.comparison. SloppySpanComparator 
 Compare two span lists using the sloppy (overlap) match criteria.
   compare(Span, Span)   - 
Method in class edu.uchsc.ccp.util.nlp.annotation.comparison. SpanComparator 
 This method will be overridden by subclasses of SpanComparator
   compare(List<Span>, List<Span>)   - 
Method in class edu.uchsc.ccp.util.nlp.annotation.comparison. SpanComparator 
 This method will be overridden by subclasses of SpanComparator
   compare(Span, Span)   - 
Method in class edu.uchsc.ccp.util.nlp.annotation.comparison. StrictSpanComparator 
 Compare two spans using the strict (exact) match criteria.
   compare(List<Span>, List<Span>)   - 
Method in class edu.uchsc.ccp.util.nlp.annotation.comparison. StrictSpanComparator 
 Compare two lists of spans using the strict (exact) match criteria.
   compare(ClassMention, ClassMention, SpanComparator, int)   - 
Method in interface edu.uchsc.ccp.util.nlp.mention.comparison. ClassMentionComparator 
  
   compare(ComplexSlotMention, ComplexSlotMention, SpanComparator, int, int)   - 
Method in interface edu.uchsc.ccp.util.nlp.mention.comparison. ComplexSlotMentionComparator 
  
   compare(Mention, Mention, SpanComparator, int, int)   - 
Method in class edu.uchsc.ccp.util.nlp.mention.comparison. IdenticalClassMentionComparator 
 Compare two mentions.
   compare(ClassMention, ClassMention, SpanComparator, int)   - 
Method in class edu.uchsc.ccp.util.nlp.mention.comparison. IdenticalClassMentionComparator 
  
   compare(Mention, Mention, SpanComparator, int, int)   - 
Method in class edu.uchsc.ccp.util.nlp.mention.comparison. IdenticalComplexSlotMentionComparator 
 Compares two mentions.
   compare(ComplexSlotMention, ComplexSlotMention, SpanComparator, int, int)   - 
Method in class edu.uchsc.ccp.util.nlp.mention.comparison. IdenticalComplexSlotMentionComparator 
 Compares two complex slot mentions.
   compare(Mention, Mention, SpanComparator, int, int)   - 
Method in class edu.uchsc.ccp.util.nlp.mention.comparison. IdenticalSlotMentionComparator 
 Compare two mentions.
   compare(SlotMention, SlotMention, SpanComparator, int, int)   - 
Method in class edu.uchsc.ccp.util.nlp.mention.comparison. IdenticalSlotMentionComparator 
 Compare two SlotMentions.
   compare(Mention, Mention)   - 
Method in class edu.uchsc.ccp.util.nlp.mention.comparison. MentionComparator 
 The default use of compare() will compare all depth levels of the mention heirarchy, and will compare any linked TextAnnotations using the
 StrictSpanComparator.
   compare(Mention, Mention, SpanComparator, int, int)   - 
Method in class edu.uchsc.ccp.util.nlp.mention.comparison. MentionComparator 
  
   compare(SlotMention, SlotMention, SpanComparator, int, int)   - 
Method in interface edu.uchsc.ccp.util.nlp.mention.comparison. SlotMentionComparator 
  
   compare(Mention, Mention)   - 
Method in class edu.uchsc.ccp.util.nlp.mention. MentionDocumentLevelSortComparator 
  
   compare(Mention, Mention)   - 
Method in class edu.uchsc.ccp.util.nlp.mention. MentionSortComparator 
  
   compareTo(Object)   - 
Method in class edu.uchsc.ccp.uima.cc.summary.comparison. AnnotationGroupID 
  
   compareTo(Object)   - 
Method in class edu.uchsc.ccp.util.nlp.annotation. AnnotationSet 
  
   compareTo(Object)   - 
Method in class edu.uchsc.ccp.util.nlp.annotation. Annotator 
  
   compareTo(Object)   - 
Method in class edu.uchsc.ccp.util.nlp.annotation. Span 
  
   compareTo(Object)   - 
Method in class edu.uchsc.ccp.util.nlp.annotation. TextAnnotation 
  
   compareTo(Object)   - 
Method in class edu.uchsc.ccp.util.nlp. KeyValuePair 
 This method enables comparison between two KeyValuePairs.
   compareTo(Object)   - 
Method in class edu.uchsc.ccp.util.nlp.mention. ClassMention 
 The default compareTo uses the IdenticalClassMentionComparator()
   compareTo(Object)   - 
Method in class edu.uchsc.ccp.util.nlp.mention. ComplexSlotMention 
  
   compareTo(Object)   - 
Method in class edu.uchsc.ccp.util.nlp.mention. Mention 
 Must be implemented by subclasses of Mention
   compareTo(Object)   - 
Method in class edu.uchsc.ccp.util.nlp.mention. SlotMention 
 Compares two slot mentions by comparing their single line representations.
   ComparisonGroup   - Class in  edu.uchsc.ccp.uima.cc.summary.comparison      ComparisonGroup()   - 
Constructor for class edu.uchsc.ccp.uima.cc.summary.comparison. ComparisonGroup 
  
   COMPLEX_SLOT_MENTION_TYPE   - 
Static variable in interface edu.uchsc.ccp.util.nlp.mention. ComplexSlotMentionTypes 
 ComplexSlotMention Identifier - if this changes, Annotation DB must be adjusted accordingly
   ComplexSlotMention   - Class in  edu.uchsc.ccp.util.nlp.mention      ComplexSlotMention()   - 
Constructor for class edu.uchsc.ccp.util.nlp.mention. ComplexSlotMention 
  Deprecated.  
   ComplexSlotMention(String)   - 
Constructor for class edu.uchsc.ccp.util.nlp.mention. ComplexSlotMention 
  
   ComplexSlotMentionComparator   - Interface in  edu.uchsc.ccp.util.nlp.mention.comparison      ComplexSlotMentionTypes   - Interface in  edu.uchsc.ccp.util.nlp.mention      computeClassicDice(Set<String>, Set<String>)   - 
Static method in class edu.uchsc.ccp.util.nlp.stats. NLPStats 
 Computes the Dice Coefficient as described in Hersh et al.
   computeClassicDice(String, String)   - 
Static method in class edu.uchsc.ccp.util.nlp.stats. NLPStats 
 Computes the Dice Coefficient as described in Hersh et al.
   computeFMeasure(double, double)   - 
Static method in class edu.uchsc.ccp.util.nlp.stats. NLPStats 
 Given precision and recall, compute the F-measure
   computeFMeasure(int, int, int)   - 
Static method in class edu.uchsc.ccp.util.nlp.stats. NLPStats 
 Given counts of TPs, FPs, and FNs, compute the F-measure
   computePrecision(int, int)   - 
Static method in class edu.uchsc.ccp.util.nlp.stats. NLPStats 
 Given counts of TPs and FPs, compute the precision
   computeRecall(int, int)   - 
Static method in class edu.uchsc.ccp.util.nlp.stats. NLPStats 
 Given counts of TPs and FNs, compute the recall
   CorpusParser   - Class in  edu.uchsc.ccp.util.nlp.parser.corpus  This is an abstract class for a corpus parser.   CorpusParser()   - 
Constructor for class edu.uchsc.ccp.util.nlp.parser.corpus. CorpusParser 
  
   create(CCPTextAnnotation, String[], String, String)   - 
Static method in class edu.uchsc.ccp.uima.util.opendmap. AnnotationString 
 Create a new standard format string for a JCas annotation.
   createDocumentLevelAnnotations(List<TextAnnotation>)   - 
Method in class edu.uchsc.ccp.util.nlp.annotation.comparison. DocumentLevelAnnotationCreator 
 Given a list of TextAnnotations, return a list of corresponding document-level annotations
   createDocumentLevelAnnotations(List<TextAnnotation>, Set<Integer>)   - 
Method in class edu.uchsc.ccp.util.nlp.annotation.comparison. DocumentLevelAnnotationCreator 
  
   createTextAnnotation(String, int, int, int, String, String, Annotator, AnnotationSet, int)   - 
Static method in class edu.uchsc.ccp.util.nlp.parser.corpus. CorpusParser 
 Creates a new TextAnnotation
   createTokenMention(String, String, String, int)   - 
Static method in class edu.uchsc.ccp.util.nlp.annotation. Annotation_Util 
  
   CYSTEINE   - 
Static variable in interface edu.uchsc.ccp.util.nlp.mention. ClassMentionTypes 
  
 
 
    
 D  
 
   DEBUG   - 
Static variable in class edu.uchsc.ccp.uima.util. UIMA_Annotation_Util 
  
   DEBUG   - 
Static variable in class edu.uchsc.ccp.uima.util. UIMASyntacticAnnotation_Util 
  
   DefaultPrintOutput   - Class in  edu.uchsc.ccp.util.nlp.annotation.printer      DefaultPrintOutput()   - 
Constructor for class edu.uchsc.ccp.util.nlp.annotation.printer. DefaultPrintOutput 
  
   DELETION   - 
Static variable in interface edu.uchsc.ccp.util.nlp.mention. ClassMentionTypes 
  
   DELETION_DELETED_ELEMENT   - 
Static variable in interface edu.uchsc.ccp.util.nlp.mention. ComplexSlotMentionTypes 
  
   determineDocumentSection(int, int[], int[])   - 
Static method in class edu.uchsc.ccp.util.nlp. GenericTextUtils 
 Determines the document section identifier for a given span based on the document section IDs and span lengths provided.
   determineOverlapWithGENIA(String, String)   - 
Static method in class edu.uchsc.ccp.util.nlp.parser.corpus. Bio1CorpusParser 
 The documents in the Bio1 corpus overlap somewhat with those in the GENIA corpus.
   DISEASE   - 
Static variable in interface edu.uchsc.ccp.util.nlp.mention. ClassMentionTypes 
  
   DMAPJcasTokenizer   - Class in  edu.uchsc.ccp.uima.util.opendmap  Helper class to get all of the appropriate annotation structures
 out of the JCas and return them as DMAPToken structures
 for the OpenDMAP parser.   DMAPJcasTokenizer(Parser, JCas, AnnotationMap, boolean)   - 
Constructor for class edu.uchsc.ccp.uima.util.opendmap. DMAPJcasTokenizer 
 Create a new iterator over structures in the JCas.
   DMAPStanfordPhraseGraph   - Class in  edu.uchsc.ccp.uima.util.opendmap  This class packages up a Stanford Phrase Tree as a DMAP Property Function Handler.   DMAPStanfordPhraseGraph(JCas, boolean)   - 
Constructor for class edu.uchsc.ccp.uima.util.opendmap. DMAPStanfordPhraseGraph 
 Create a new DMAPStanfordPhraseGraph property function handler from
 the annotations the Stanford Parser has left in the UIMA CAS.
   DNA   - 
Static variable in interface edu.uchsc.ccp.util.nlp.mention. ClassMentionTypes 
  
   DOCUMENT_COLLECTION_ID   - 
Static variable in class edu.uchsc.ccp.util.nlp.parser.corpus. Bio1CorpusParser 
  
   DOCUMENT_COLLECTION_ID   - 
Static variable in class edu.uchsc.ccp.util.nlp.parser.corpus. BioIECorpusParser 
  
   DOCUMENT_COLLECTION_ID   - 
Static variable in class edu.uchsc.ccp.util.nlp.parser.corpus. GeniaCorpusParser 
  
   DOCUMENT_COLLECTION_ID   - 
Static variable in class edu.uchsc.ccp.util.nlp.parser.corpus. IProLinkCorpus1Parser 
  
   DOCUMENT_COLLECTION_ID   - 
Static variable in class edu.uchsc.ccp.util.nlp.parser.corpus. IProLinkCorpus2Parser 
  
   DOCUMENT_COLLECTION_ID   - 
Static variable in class edu.uchsc.ccp.util.nlp.parser.corpus. TexasCorpusParser 
  
   DOCUMENT_COLLECTION_ID   - 
Static variable in class edu.uchsc.ccp.util.nlp.parser.corpus. YapexCorpusParser 
  
   DOCUMENT_FIGURE_LEGEND   - 
Static variable in interface edu.uchsc.ccp.util.nlp.mention. ClassMentionTypes 
  
   DOCUMENT_SECTION   - 
Static variable in interface edu.uchsc.ccp.util.nlp.mention. ClassMentionTypes 
  
   DOCUMENT_SECTION_HEADING   - 
Static variable in interface edu.uchsc.ccp.util.nlp.mention. ClassMentionTypes 
  
   DOCUMENT_SECTION_NAME   - 
Static variable in interface edu.uchsc.ccp.util.nlp.mention. SlotMentionTypes 
 Document Section and SubSection slots
   DOCUMENT_SUBSECTION   - 
Static variable in interface edu.uchsc.ccp.util.nlp.mention. ClassMentionTypes 
  
   DOCUMENT_SUBSECTION_HEADING   - 
Static variable in interface edu.uchsc.ccp.util.nlp.mention. ClassMentionTypes 
  
   DOCUMENT_SUBSECTION_NAME   - 
Static variable in interface edu.uchsc.ccp.util.nlp.mention. SlotMentionTypes 
  
   DOCUMENT_TABLE_LEGEND   - 
Static variable in interface edu.uchsc.ccp.util.nlp.mention. ClassMentionTypes 
  
   DOCUMENT_TITLE   - 
Static variable in interface edu.uchsc.ccp.util.nlp.mention. ClassMentionTypes 
  
   DocumentLevelAnnotationCreator   - Class in  edu.uchsc.ccp.util.nlp.annotation.comparison  This class creates document-level annotations for a given type.   DocumentLevelAnnotationCreator(String)   - 
Constructor for class edu.uchsc.ccp.util.nlp.annotation.comparison. DocumentLevelAnnotationCreator 
 This DocumentLevelAnnotationCreator will generate document-level annotations for all annotations of type mentionType.
   DocumentLevelAnnotationCreator(String, Set<String>)   - 
Constructor for class edu.uchsc.ccp.util.nlp.annotation.comparison. DocumentLevelAnnotationCreator 
 This DocumentLevelAnnotationCreator will generate document-level annotations for only those annotations that have non-empty slots specified by
 the requiredSlots Set.
   DocumentLevelAnnotationCreator_AE   - Class in  edu.uchsc.ccp.uima.ae.util.converter  creates document-level annotations of the specified type.   DocumentLevelAnnotationCreator_AE()   - 
Constructor for class edu.uchsc.ccp.uima.ae.util.converter. DocumentLevelAnnotationCreator_AE 
  
   DocumentSection   - Class in  edu.uchsc.ccp.util.nlp.document      DocumentSection()   - 
Constructor for class edu.uchsc.ccp.util.nlp.document. DocumentSection 
  
   DocumentSectionTypes   - Interface in  edu.uchsc.ccp.util.nlp.document      DocumentUtil   - Class in  edu.uchsc.ccp.util.nlp.document      DocumentUtil()   - 
Constructor for class edu.uchsc.ccp.util.nlp.document. DocumentUtil 
  
 
 
    
 E  
 
   edu.uchsc.ccp.uima.ae.nlp.anaphora   - package edu.uchsc.ccp.uima.ae.nlp.anaphora     edu.uchsc.ccp.uima.ae.nlp.ei.gene   - package edu.uchsc.ccp.uima.ae.nlp.ei.gene     edu.uchsc.ccp.uima.ae.nlp.ei.mutation   - package edu.uchsc.ccp.uima.ae.nlp.ei.mutation     edu.uchsc.ccp.uima.ae.nlp.opendmap   - package edu.uchsc.ccp.uima.ae.nlp.opendmap     edu.uchsc.ccp.uima.ae.nlp.parser   - package edu.uchsc.ccp.uima.ae.nlp.parser     edu.uchsc.ccp.uima.ae.nlp.sentenceDetector   - package edu.uchsc.ccp.uima.ae.nlp.sentenceDetector     edu.uchsc.ccp.uima.ae.nlp.stemmer   - package edu.uchsc.ccp.uima.ae.nlp.stemmer     edu.uchsc.ccp.uima.ae.nlp.tokenizer   - package edu.uchsc.ccp.uima.ae.nlp.tokenizer     edu.uchsc.ccp.uima.ae.util   - package edu.uchsc.ccp.uima.ae.util     edu.uchsc.ccp.uima.ae.util.converter   - package edu.uchsc.ccp.uima.ae.util.converter     edu.uchsc.ccp.uima.ae.util.filter   - package edu.uchsc.ccp.uima.ae.util.filter     edu.uchsc.ccp.uima.annotation   - package edu.uchsc.ccp.uima.annotation     edu.uchsc.ccp.uima.annotation.syntactic   - package edu.uchsc.ccp.uima.annotation.syntactic     edu.uchsc.ccp.uima.cc.summary   - package edu.uchsc.ccp.uima.cc.summary     edu.uchsc.ccp.uima.cc.summary.comparison   - package edu.uchsc.ccp.uima.cc.summary.comparison     edu.uchsc.ccp.uima.cc.summary.opendmap   - package edu.uchsc.ccp.uima.cc.summary.opendmap     edu.uchsc.ccp.uima.mention   - package edu.uchsc.ccp.uima.mention     edu.uchsc.ccp.uima.util   - package edu.uchsc.ccp.uima.util     edu.uchsc.ccp.uima.util.opendmap   - package edu.uchsc.ccp.uima.util.opendmap     edu.uchsc.ccp.util   - package edu.uchsc.ccp.util     edu.uchsc.ccp.util.external.abgene   - package edu.uchsc.ccp.util.external.abgene     edu.uchsc.ccp.util.external.abner   - package edu.uchsc.ccp.util.external.abner     edu.uchsc.ccp.util.external.geniatagger   - package edu.uchsc.ccp.util.external.geniatagger     edu.uchsc.ccp.util.external.kex   - package edu.uchsc.ccp.util.external.kex     edu.uchsc.ccp.util.external.lingpipe   - package edu.uchsc.ccp.util.external.lingpipe     edu.uchsc.ccp.util.external.opennlp   - package edu.uchsc.ccp.util.external.opennlp     edu.uchsc.ccp.util.external.penn   - package edu.uchsc.ccp.util.external.penn     edu.uchsc.ccp.util.external.stanfordparser   - package edu.uchsc.ccp.util.external.stanfordparser     edu.uchsc.ccp.util.nlp   - package edu.uchsc.ccp.util.nlp     edu.uchsc.ccp.util.nlp.annotation   - package edu.uchsc.ccp.util.nlp.annotation     edu.uchsc.ccp.util.nlp.annotation.comparison   - package edu.uchsc.ccp.util.nlp.annotation.comparison     edu.uchsc.ccp.util.nlp.annotation.filter   - package edu.uchsc.ccp.util.nlp.annotation.filter     edu.uchsc.ccp.util.nlp.annotation.printer   - package edu.uchsc.ccp.util.nlp.annotation.printer     edu.uchsc.ccp.util.nlp.document   - package edu.uchsc.ccp.util.nlp.document     edu.uchsc.ccp.util.nlp.ei   - package edu.uchsc.ccp.util.nlp.ei     edu.uchsc.ccp.util.nlp.ei.xml   - package edu.uchsc.ccp.util.nlp.ei.xml     edu.uchsc.ccp.util.nlp.mention   - package edu.uchsc.ccp.util.nlp.mention     edu.uchsc.ccp.util.nlp.mention.comparison   - package edu.uchsc.ccp.util.nlp.mention.comparison     edu.uchsc.ccp.util.nlp.parser.corpus   - package edu.uchsc.ccp.util.nlp.parser.corpus     edu.uchsc.ccp.util.nlp.stats   - package edu.uchsc.ccp.util.nlp.stats     edu.uchsc.ccp.util.nlp.stemmer   - package edu.uchsc.ccp.util.nlp.stemmer     edu.uchsc.ccp.util.nlp.tokenizer   - package edu.uchsc.ccp.util.nlp.tokenizer     edu.uchsc.ccp.util.xml   - package edu.uchsc.ccp.util.xml     EIFileBasedSystem   - Interface in  edu.uchsc.ccp.util.nlp.ei      EISystem   - Interface in  edu.uchsc.ccp.util.nlp.ei      endsBefore(Span)   - 
Method in class edu.uchsc.ccp.util.nlp.annotation. Span 
 Returns true if this span ends before the inputted span, false otherwise.
   EntityPartsAnnotationComparator   - Class in  edu.uchsc.ccp.util.nlp.annotation.comparison  This class implements the functionality of the "Protein Name Parts" comparison metric described in Olson et al... 
 The usefulness of this kind of comparison is limited to simple entity mentions, therefore, the mention comparison will compare the entire mention
 hierarchy, however, the entity parts computation will only take place on the base annotation span. 
  
 For example, if for some reason you wanted to run the entity-parts metric on a group of transport annotations, where each transport mention has
 complex slot mentions (transported entities, source, destination, transport participants), then this method will 
 
  find equivalent transport annotations using the SloppySpanComparator
  if equivalent annotations are found, then the covered text for each annotation (and in this case, just for the transport annotation, not any
 of its slot fillers) will be tokenized on whitespace, and the entity-parts metric will be done using those tokens.   EntityPartsAnnotationComparator()   - 
Constructor for class edu.uchsc.ccp.util.nlp.annotation.comparison. EntityPartsAnnotationComparator 
  
   equalComplexSlotMentions(List<ComplexSlotMention>, List<ComplexSlotMention>, SpanComparator, ComplexSlotMentionComparator, int, int)   - 
Method in class edu.uchsc.ccp.util.nlp.mention.comparison. MentionComparator 
 This method compares two lists of complex slot mentions by iterating through them.
   equals(Object)   - 
Method in class edu.uchsc.ccp.uima.cc.summary.comparison. AnnotationGroupID 
  
   equals(Object)   - 
Method in class edu.uchsc.ccp.util.nlp.annotation. AnnotationSet 
  
   equals(Object)   - 
Method in class edu.uchsc.ccp.util.nlp.annotation. Annotator 
  
   equals(Object)   - 
Method in class edu.uchsc.ccp.util.nlp.annotation. Span 
 The default equals() method uses the StrictSpanComparator
   equals(Object)   - 
Method in class edu.uchsc.ccp.util.nlp.annotation. TextAnnotation 
 The default equals() method requires exact span match, as well as identical class mention match to return true.
   equals(Object)   - 
Method in class edu.uchsc.ccp.util.nlp.mention. ClassMention 
 The default equals() method uses the IdenticalClassMentionComparator.
   equals(Object)   - 
Method in class edu.uchsc.ccp.util.nlp.mention. ComplexSlotMention 
  
   equals(Object)   - 
Method in class edu.uchsc.ccp.util.nlp.mention. SlotMention 
 Two slot mentions are equal if their slot mention name is equal, and their slot values are equal
   equals(SlotMention, SlotMentionComparator, SpanComparator, int, int)   - 
Method in class edu.uchsc.ccp.util.nlp.mention. SlotMention 
  
   equalSlotMentions(List<SlotMention>, List<SlotMention>, SpanComparator, SlotMentionComparator, int, int)   - 
Method in class edu.uchsc.ccp.util.nlp.mention.comparison. MentionComparator 
 This method compares two lists of slot mentions by iterating through them.
   error(String)   - 
Method in class edu.uchsc.ccp.util.nlp.parser.corpus. CorpusParser 
  
   escapeCharacterForRegEx(String)   - 
Static method in class edu.uchsc.ccp.util.nlp. GenericTextUtils 
 Prepares a string to be used in a Regular Expression search.
   evalFunction(String, String, Reference)   - 
Method in class edu.uchsc.ccp.uima.util.opendmap. DMAPStanfordPhraseGraph 
 Evaluate a function.
   evaluateSentenceDetector(String, String)   - 
Static method in class edu.uchsc.ccp.util.external.lingpipe. LingPipe_Util 
 Evaluate the LingPipe Sentence detector Taken from:
 http://alias-i.com/lingpipe/demos/tutorial/sentences/src/SentenceModelEvaluator.java
   exampleDocText()   - 
Static method in class edu.uchsc.ccp.util.nlp. GenericTextUtils 
 Returns some sample text
   EXPRESSION   - 
Static variable in interface edu.uchsc.ccp.util.nlp.mention. ClassMentionTypes 
 Semantic predicates
   EXPRESSION_EXPRESSED_GENE_OR_GENE_PRODUCT   - 
Static variable in interface edu.uchsc.ccp.util.nlp.mention. SlotMentionTypes 
  
   EXPRESSION_LOCATION   - 
Static variable in interface edu.uchsc.ccp.util.nlp.mention. SlotMentionTypes 
  
   extractEntities(String, String)   - 
Static method in class edu.uchsc.ccp.util.external.lingpipe. LingPipe_Util 
 Use with Lingpipe_3.0.0
 
 
    
 F  
 
   featOkTst   - 
Static variable in class edu.uchsc.ccp.uima.annotation. CCPAnnotation_Type 
  
   featOkTst   - 
Static variable in class edu.uchsc.ccp.uima.annotation. CCPAnnotationSet_Type 
  
   featOkTst   - 
Static variable in class edu.uchsc.ccp.uima.annotation. CCPAnnotator_Type 
  
   featOkTst   - 
Static variable in class edu.uchsc.ccp.uima.annotation. CCPDocumentAnnotation_Type 
  
   featOkTst   - 
Static variable in class edu.uchsc.ccp.uima.annotation. CCPDocumentInformation_Type 
  
   featOkTst   - 
Static variable in class edu.uchsc.ccp.uima.annotation. CCPDocumentSection_Type 
  
   featOkTst   - 
Static variable in class edu.uchsc.ccp.uima.annotation. CCPDocumentSubSection_Type 
  
   featOkTst   - 
Static variable in class edu.uchsc.ccp.uima.annotation. CCPSemanticAnnotation_Type 
  
   featOkTst   - 
Static variable in class edu.uchsc.ccp.uima.annotation. CCPSpan_Type 
  
   featOkTst   - 
Static variable in class edu.uchsc.ccp.uima.annotation. CCPSyntacticAnnotation_Type 
  
   featOkTst   - 
Static variable in class edu.uchsc.ccp.uima.annotation. CCPTextAnnotation_Type 
  
   featOkTst   - 
Static variable in class edu.uchsc.ccp.uima.annotation.syntactic. CCPClauseAnnotation_Type 
  
   featOkTst   - 
Static variable in class edu.uchsc.ccp.uima.annotation.syntactic. CCPPhraseAnnotation_Type 
  
   featOkTst   - 
Static variable in class edu.uchsc.ccp.uima.annotation.syntactic. CCPSentenceAnnotation_Type 
  
   featOkTst   - 
Static variable in class edu.uchsc.ccp.uima.annotation.syntactic. CCPTokenAnnotation_Type 
  
   featOkTst   - 
Static variable in class edu.uchsc.ccp.uima.annotation.syntactic. CCPTypedDependency_Type 
  
   featOkTst   - 
Static variable in class edu.uchsc.ccp.uima.mention. CCPClassMention_Type 
  
   featOkTst   - 
Static variable in class edu.uchsc.ccp.uima.mention. CCPComplexSlotMention_Type 
  
   featOkTst   - 
Static variable in class edu.uchsc.ccp.uima.mention. CCPMention_Type 
  
   featOkTst   - 
Static variable in class edu.uchsc.ccp.uima.mention. CCPNonComplexSlotMention_Type 
  
   featOkTst   - 
Static variable in class edu.uchsc.ccp.uima.mention. CCPSlotMention_Type 
  
   FIGURE_ID   - 
Static variable in interface edu.uchsc.ccp.util.nlp.mention. SlotMentionTypes 
  
   findFirstNonHTMLCharacter(String)   - 
Static method in class edu.uchsc.ccp.util.nlp. GenericTextUtils 
  
   findLastNonHTMLCharacter(String)   - 
Static method in class edu.uchsc.ccp.util.nlp. GenericTextUtils 
  
   findSentences(String, String)   - 
Static method in class edu.uchsc.ccp.util.external.lingpipe. LingPipe_Util 
 Use SentenceModel to find sentence boundaries in text LingPipe relevant code used by this method was taken from:
 http://alias-i.com/lingpipe/demos/tutorial/sentences/src/SentenceChunkerDemo.java
   findTargetInText(String, String, int, String, String, Annotator, boolean)   - 
Static method in class edu.uchsc.ccp.util.nlp. GenericTextUtils 
 This method simple returns text annotations created from finding the target string within the original text string using regular expressions
 
 
    
 G  
 
   GENE   - 
Static variable in interface edu.uchsc.ccp.util.nlp.mention. ClassMentionTypes 
  
   GENE_GENERIC   - 
Static variable in interface edu.uchsc.ccp.util.nlp.mention. ClassMentionTypes 
  
   GENE_OR_GENE_PRODUCT   - 
Static variable in interface edu.uchsc.ccp.util.nlp.mention. ClassMentionTypes 
  
   GenericDocument   - Class in  edu.uchsc.ccp.util.nlp.document      GenericDocument(String)   - 
Constructor for class edu.uchsc.ccp.util.nlp.document. GenericDocument 
  
   GenericDocument()   - 
Constructor for class edu.uchsc.ccp.util.nlp.document. GenericDocument 
  
   GenericTextUtils   - Class in  edu.uchsc.ccp.util.nlp  This class encompasses a collection of utilities for processing and dealing with text.   GenericTextUtils()   - 
Constructor for class edu.uchsc.ccp.util.nlp. GenericTextUtils 
  
   GENIA_atom   - 
Static variable in interface edu.uchsc.ccp.util.nlp.mention. ClassMentionTypes 
  
   GENIA_body_part   - 
Static variable in interface edu.uchsc.ccp.util.nlp.mention. ClassMentionTypes 
  
   GENIA_carbohydrate   - 
Static variable in interface edu.uchsc.ccp.util.nlp.mention. ClassMentionTypes 
  
   GENIA_cell_component   - 
Static variable in interface edu.uchsc.ccp.util.nlp.mention. ClassMentionTypes 
  
   GENIA_DNA_domain_or_region   - 
Static variable in interface edu.uchsc.ccp.util.nlp.mention. ClassMentionTypes 
  
   GENIA_DNA_family_or_group   - 
Static variable in interface edu.uchsc.ccp.util.nlp.mention. ClassMentionTypes 
  
   GENIA_DNA_NA   - 
Static variable in interface edu.uchsc.ccp.util.nlp.mention. ClassMentionTypes 
  
   GENIA_DNA_substructure   - 
Static variable in interface edu.uchsc.ccp.util.nlp.mention. ClassMentionTypes 
  
   GENIA_inorganic   - 
Static variable in interface edu.uchsc.ccp.util.nlp.mention. ClassMentionTypes 
  
   GENIA_lipid   - 
Static variable in interface edu.uchsc.ccp.util.nlp.mention. ClassMentionTypes 
  
   GENIA_mono_cell   - 
Static variable in interface edu.uchsc.ccp.util.nlp.mention. ClassMentionTypes 
  
   GENIA_multi_cell   - 
Static variable in interface edu.uchsc.ccp.util.nlp.mention. ClassMentionTypes 
  
   GENIA_nucleotide   - 
Static variable in interface edu.uchsc.ccp.util.nlp.mention. ClassMentionTypes 
  
   GENIA_other_artificial_source   - 
Static variable in interface edu.uchsc.ccp.util.nlp.mention. ClassMentionTypes 
 For GENIA tagger
   GENIA_other_name   - 
Static variable in interface edu.uchsc.ccp.util.nlp.mention. ClassMentionTypes 
  
   GENIA_other_organic_compound   - 
Static variable in interface edu.uchsc.ccp.util.nlp.mention. ClassMentionTypes 
  
   GENIA_peptide   - 
Static variable in interface edu.uchsc.ccp.util.nlp.mention. ClassMentionTypes 
  
   GENIA_polynucleotide   - 
Static variable in interface edu.uchsc.ccp.util.nlp.mention. ClassMentionTypes 
  
   GENIA_protein_domain_or_region   - 
Static variable in interface edu.uchsc.ccp.util.nlp.mention. ClassMentionTypes 
  
   GENIA_protein_family_or_group   - 
Static variable in interface edu.uchsc.ccp.util.nlp.mention. ClassMentionTypes 
  
   GENIA_protein_NA   - 
Static variable in interface edu.uchsc.ccp.util.nlp.mention. ClassMentionTypes 
  
   GENIA_protein_substructure   - 
Static variable in interface edu.uchsc.ccp.util.nlp.mention. ClassMentionTypes 
  
   GENIA_protein_subunit   - 
Static variable in interface edu.uchsc.ccp.util.nlp.mention. ClassMentionTypes 
  
   GENIA_RNA_domain_or_region   - 
Static variable in interface edu.uchsc.ccp.util.nlp.mention. ClassMentionTypes 
  
   GENIA_RNA_family_or_group   - 
Static variable in interface edu.uchsc.ccp.util.nlp.mention. ClassMentionTypes 
  
   GENIA_RNA_NA   - 
Static variable in interface edu.uchsc.ccp.util.nlp.mention. ClassMentionTypes 
  
   GENIA_RNA_substructure   - 
Static variable in interface edu.uchsc.ccp.util.nlp.mention. ClassMentionTypes 
  
   GeniaCorpusParser   - Class in  edu.uchsc.ccp.util.nlp.parser.corpus  A simple parser for the GENIA corpus which utilizes the LingPipe utilities in order to extract entities and sentences from the corpus.   GeniaCorpusParser(String)   - 
Constructor for class edu.uchsc.ccp.util.nlp.parser.corpus. GeniaCorpusParser 
  
   GeniaTaggerOutputFileLoader_AE   - Class in  edu.uchsc.ccp.uima.ae.nlp.tokenizer  This class loads in a file which contains GENIA tagger output.   GeniaTaggerOutputFileLoader_AE()   - 
Constructor for class edu.uchsc.ccp.uima.ae.nlp.tokenizer. GeniaTaggerOutputFileLoader_AE 
  
   GeniaTaggerOutputFileParser   - Class in  edu.uchsc.ccp.util.external.geniatagger      GeniaTaggerOutputFileParser(String)   - 
Constructor for class edu.uchsc.ccp.util.external.geniatagger. GeniaTaggerOutputFileParser 
  
   getAbstractText()   - 
Method in class edu.uchsc.ccp.util.nlp.document. GenericDocument 
  Deprecated.  
   getAffiliation()   - 
Method in class edu.uchsc.ccp.uima.annotation. CCPAnnotator 
 getter for affiliation - gets The affiliation of the annotator.
   getAffiliation(int)   - 
Method in class edu.uchsc.ccp.uima.annotation. CCPAnnotator_Type 
  
   getAffiliation()   - 
Method in class edu.uchsc.ccp.util.nlp.annotation. Annotator 
  
   getAggregateSpan()   - 
Method in class edu.uchsc.ccp.util.nlp.annotation. TextAnnotation 
 Returns the total span of this annotation (i.e.
   getAnnotationGroupList()   - 
Method in class edu.uchsc.ccp.uima.cc.summary.comparison. ComparisonGroup 
  
   getAnnotationGroupMap()   - 
Method in class edu.uchsc.ccp.uima.cc.summary.comparison. ComparatorConfigurator 
  
   getAnnotationID()   - 
Method in class edu.uchsc.ccp.uima.annotation. CCPTextAnnotation 
 getter for annotationID - gets The annotation ID provides a means for identifying a particular annotation.
   getAnnotationID(int)   - 
Method in class edu.uchsc.ccp.uima.annotation. CCPTextAnnotation_Type 
  
   getAnnotationID()   - 
Method in class edu.uchsc.ccp.util.nlp.annotation. TextAnnotation 
 Get the annotation ID
   getAnnotations()   - 
Method in class edu.uchsc.ccp.util.nlp.document. GenericDocument 
  
   getAnnotationsEncompassingSpan(Span, JCas)   - 
Static method in class edu.uchsc.ccp.uima.util. UIMA_Util 
  
   getAnnotationsEncompassingSpan(Span, JCas, int)   - 
Static method in class edu.uchsc.ccp.uima.util. UIMA_Util 
 return all annotations that contain the input span
   getAnnotationSetDescription()   - 
Method in class edu.uchsc.ccp.uima.annotation. CCPAnnotationSet 
 getter for annotationSetDescription - gets A textual description of an annotation set.
   getAnnotationSetDescription(int)   - 
Method in class edu.uchsc.ccp.uima.annotation. CCPAnnotationSet_Type 
  
   getAnnotationSetDescription()   - 
Method in class edu.uchsc.ccp.util.nlp.annotation. AnnotationSet 
  
   getAnnotationSetID()   - 
Method in class edu.uchsc.ccp.uima.annotation. CCPAnnotationSet 
 getter for annotationSetID - gets An integer uniquely identifying a particular annotation set.
   getAnnotationSetID(int)   - 
Method in class edu.uchsc.ccp.uima.annotation. CCPAnnotationSet_Type 
  
   getAnnotationSetID()   - 
Method in class edu.uchsc.ccp.uima.cc.summary.comparison. AnnotationGroup 
  
   getAnnotationSetID()   - 
Method in class edu.uchsc.ccp.util.nlp.annotation. AnnotationSet 
  
   getAnnotationSetIDs()   - 
Method in class edu.uchsc.ccp.util.nlp.annotation. TextAnnotation 
 Return a set containing the AnnotationSet IDs for which this TextAnnotation is a member.
   getAnnotationSetName()   - 
Method in class edu.uchsc.ccp.uima.annotation. CCPAnnotationSet 
 getter for annotationSetName - gets The name of the annotation set.
   getAnnotationSetName(int)   - 
Method in class edu.uchsc.ccp.uima.annotation. CCPAnnotationSet_Type 
  
   getAnnotationSetName()   - 
Method in class edu.uchsc.ccp.util.nlp.annotation. AnnotationSet 
  
   getAnnotationSets()   - 
Method in class edu.uchsc.ccp.uima.annotation. CCPTextAnnotation 
 getter for annotationSets - gets Annotation Sets provide an arbitrary means of categorizing and clustering annotations into groups.
   getAnnotationSets(int)   - 
Method in class edu.uchsc.ccp.uima.annotation. CCPTextAnnotation 
 indexed getter for annotationSets - gets an indexed value - Annotation Sets provide an arbitrary means of categorizing and clustering annotations into groups.
   getAnnotationSets(int)   - 
Method in class edu.uchsc.ccp.uima.annotation. CCPTextAnnotation_Type 
  
   getAnnotationSets(int, int)   - 
Method in class edu.uchsc.ccp.uima.annotation. CCPTextAnnotation_Type 
  
   getAnnotationSets()   - 
Method in class edu.uchsc.ccp.util.nlp.annotation. TextAnnotation 
 Get the annotation sets
   getAnnotationsInBetweenSpans(Span, Span, JCas)   - 
Static method in class edu.uchsc.ccp.uima.util. UIMA_Util 
 return annotations in between spans
   getAnnotationSpanEnd()   - 
Method in class edu.uchsc.ccp.util.nlp.annotation. TextAnnotation 
 Return the end index of the last span (spans are stored in order)
   getAnnotationSpanStart()   - 
Method in class edu.uchsc.ccp.util.nlp.annotation. TextAnnotation 
 Return the start index of the first span (spans are stored in order)
   getAnnotationsWithinSpan(Span, JCas)   - 
Static method in class edu.uchsc.ccp.uima.util. UIMA_Util 
  
   getAnnotationsWithinSpan(Span, JCas, int)   - 
Static method in class edu.uchsc.ccp.uima.util. UIMA_Util 
 return all annotations within the exact same span as the input span
   getAnnotationsWithSameStart(int, JCas)   - 
Static method in class edu.uchsc.ccp.uima.util. UIMA_Util 
 return all annotations that start with the input startIndex
   getAnnotationTypeList()   - 
Method in class edu.uchsc.ccp.uima.cc.summary.comparison. AnnotationGroup 
  
   getAnnotator()   - 
Method in class edu.uchsc.ccp.uima.annotation. CCPTextAnnotation 
 getter for annotator - gets The annotator was responsible for generating this annotation.
   getAnnotator(int)   - 
Method in class edu.uchsc.ccp.uima.annotation. CCPTextAnnotation_Type 
  
   getAnnotator()   - 
Method in class edu.uchsc.ccp.util.nlp.annotation. TextAnnotation 
 Set the annotator
   getAnnotatorID()   - 
Method in class edu.uchsc.ccp.uima.annotation. CCPAnnotator 
 getter for annotatorID - gets This Integer should be a unique ID for a particular annotator.
   getAnnotatorID(int)   - 
Method in class edu.uchsc.ccp.uima.annotation. CCPAnnotator_Type 
  
   getAnnotatorID()   - 
Method in class edu.uchsc.ccp.uima.cc.summary.comparison. AnnotationGroup 
  
   getAnnotatorID()   - 
Static method in class edu.uchsc.ccp.util.external.lingpipe. LingPipe_Util 
  
   getAnnotatorID()   - 
Method in class edu.uchsc.ccp.util.nlp.annotation. Annotator 
  
   getAnnotatorID()   - 
Method in class edu.uchsc.ccp.util.nlp.annotation. TextAnnotation 
 This method returns the corresponding annotator ID
   getCcpTextAnnotations()   - 
Method in class edu.uchsc.ccp.uima.mention. CCPClassMention 
 getter for ccpTextAnnotations - gets Just as CCPTextAnnotations are linked to a CCPClassMention, it is sometimes useful to be able to follow a CCPClassMention back to its corresponding CCPTextAnnotations, therefore, this FSArray contains links to the CCPTextAnotation(s) for this class.
   getCcpTextAnnotations(int)   - 
Method in class edu.uchsc.ccp.uima.mention. CCPClassMention 
 indexed getter for ccpTextAnnotations - gets an indexed value - Just as CCPTextAnnotations are linked to a CCPClassMention, it is sometimes useful to be able to follow a CCPClassMention back to its corresponding CCPTextAnnotations, therefore, this FSArray contains links to the CCPTextAnotation(s) for this class.
   getCcpTextAnnotations(int)   - 
Method in class edu.uchsc.ccp.uima.mention. CCPClassMention_Type 
  
   getCcpTextAnnotations(int, int)   - 
Method in class edu.uchsc.ccp.uima.mention. CCPClassMention_Type 
  
   getClassificationType()   - 
Method in class edu.uchsc.ccp.uima.annotation. CCPDocumentInformation 
 getter for classificationType - gets This String provides a means for classifying a particular document.
   getClassificationType(int)   - 
Method in class edu.uchsc.ccp.uima.annotation. CCPDocumentInformation_Type 
  
   getClassMention()   - 
Method in class edu.uchsc.ccp.uima.annotation. CCPTextAnnotation 
 getter for classMention - gets The CCP ClassMention indicates the type (or class) for this annotation.
   getClassMention(int)   - 
Method in class edu.uchsc.ccp.uima.annotation. CCPTextAnnotation_Type 
  
   getClassMention()   - 
Method in class edu.uchsc.ccp.util.nlp.annotation. TextAnnotation 
  
   getClassMentions()   - 
Method in class edu.uchsc.ccp.uima.mention. CCPComplexSlotMention 
 getter for classMentions - gets The class mentions which are the slot fillers for this complex slot.
   getClassMentions(int)   - 
Method in class edu.uchsc.ccp.uima.mention. CCPComplexSlotMention 
 indexed getter for classMentions - gets an indexed value - The class mentions which are the slot fillers for this complex slot.
   getClassMentions(int)   - 
Method in class edu.uchsc.ccp.uima.mention. CCPComplexSlotMention_Type 
  
   getClassMentions(int, int)   - 
Method in class edu.uchsc.ccp.uima.mention. CCPComplexSlotMention_Type 
  
   getClassMentions()   - 
Method in class edu.uchsc.ccp.util.nlp.mention. ComplexSlotMention 
 Get the class mentions that fill this complex slot
   getClauseType()   - 
Method in class edu.uchsc.ccp.uima.annotation.syntactic. CCPClauseAnnotation 
 getter for clauseType - gets The clause type symbol is stored here.
   getClauseType(int)   - 
Method in class edu.uchsc.ccp.uima.annotation.syntactic. CCPClauseAnnotation_Type 
  
   getComparisonGroupList()   - 
Method in class edu.uchsc.ccp.uima.cc.summary.comparison. ComparatorConfigurator 
  
   getComplexSlotMentionByName(String)   - 
Method in class edu.uchsc.ccp.util.nlp.mention. ClassMention 
  Deprecated.  
   getComplexSlotMentions()   - 
Method in class edu.uchsc.ccp.util.nlp.mention. ClassMention 
  
   getComplexSlotMentionsByName(String)   - 
Method in class edu.uchsc.ccp.util.nlp.mention. ClassMention 
 Return all complex slot mentions for a given name.
   getCoveredText()   - 
Method in class edu.uchsc.ccp.util.nlp.annotation. TextAnnotation 
 Return the text that is "covered" by this annotation
   getDependentTokenNum()   - 
Method in class edu.uchsc.ccp.uima.annotation.syntactic. CCPTypedDependency 
 getter for dependentTokenNum - gets
   getDependentTokenNum(int)   - 
Method in class edu.uchsc.ccp.uima.annotation.syntactic. CCPTypedDependency_Type 
  
   getDependentTokenNum()   - 
Method in class edu.uchsc.ccp.util.external.stanfordparser. TypedDependencyCls 
  
   getDescription()   - 
Method in class edu.uchsc.ccp.uima.cc.summary.comparison. ComparisonGroup 
  
   getDocumentCollectionID()   - 
Method in class edu.uchsc.ccp.uima.annotation. CCPDocumentInformation 
 getter for documentCollectionID - gets The document collection ID is an Integer that uniquely identifies a particular document collection.
   getDocumentCollectionID(int)   - 
Method in class edu.uchsc.ccp.uima.annotation. CCPDocumentInformation_Type 
  
   getDocumentCollectionID()   - 
Method in class edu.uchsc.ccp.util.nlp.annotation. TextAnnotation 
 Get the document collection ID
   getDocumentCollectionID()   - 
Method in class edu.uchsc.ccp.util.nlp.document. GenericDocument 
  
   getDocumentID()   - 
Method in class edu.uchsc.ccp.uima.annotation. CCPDocumentInformation 
 getter for documentID - gets The document ID is a String representing a unique identifier for a particular document within a particular document collection.
   getDocumentID(int)   - 
Method in class edu.uchsc.ccp.uima.annotation. CCPDocumentInformation_Type 
  
   getDocumentID(JCas)   - 
Static method in class edu.uchsc.ccp.uima.util. UIMA_Util 
  
   getDocumentID()   - 
Method in class edu.uchsc.ccp.util.nlp.annotation. TextAnnotation 
 Get the document ID
   getDocumentID()   - 
Method in class edu.uchsc.ccp.util.nlp.document. GenericDocument 
  
   getDocumentLevelSingleLineRepresentation(boolean)   - 
Method in class edu.uchsc.ccp.util.nlp.annotation. TextAnnotation 
 Same a single line representation, however no span, covered text, or annotation meta info, is shown..
   getDocumentLevelSingleLineRepresentation()   - 
Method in class edu.uchsc.ccp.util.nlp.mention. ClassMention 
 similar to getSingleLineRepresentation, however this is a document-level representation, so no meta data are included (span info, covered text, etc.)
   getDocumentLevelSingleLineRepresentation()   - 
Method in class edu.uchsc.ccp.util.nlp.mention. ComplexSlotMention 
  
   getDocumentLevelSingleLineRepresentation()   - 
Method in class edu.uchsc.ccp.util.nlp.mention. Mention 
  
   getDocumentLevelSingleLineRepresentation()   - 
Method in class edu.uchsc.ccp.util.nlp.mention. SlotMention 
  
   getDocuments()   - 
Static method in class edu.uchsc.ccp.util.nlp.parser.corpus. BioIECorpusParser 
  
   getDocuments()   - 
Static method in class edu.uchsc.ccp.util.nlp.parser.corpus. IProLinkParser 
  
   getDocuments()   - 
Static method in class edu.uchsc.ccp.util.nlp.parser.corpus. TexasCorpusParser 
  
   getDocumentSectionID()   - 
Method in class edu.uchsc.ccp.uima.annotation. CCPTextAnnotation 
 getter for documentSectionID - gets The document section ID is optionally used to log what section of a document this annotation is from.
   getDocumentSectionID(int)   - 
Method in class edu.uchsc.ccp.uima.annotation. CCPTextAnnotation_Type 
  
   getDocumentSectionID()   - 
Method in class edu.uchsc.ccp.util.nlp.annotation. TextAnnotation 
 Get the document section ID
   getDocumentSectionID()   - 
Method in class edu.uchsc.ccp.util.nlp.document. DocumentSection 
  
   getDocumentSections()   - 
Method in class edu.uchsc.ccp.util.nlp.document. GenericDocument 
  
   getDocumentSize()   - 
Method in class edu.uchsc.ccp.uima.annotation. CCPDocumentInformation 
 getter for documentSize - gets The size of a document is logged as the number of characters it contains.
   getDocumentSize(int)   - 
Method in class edu.uchsc.ccp.uima.annotation. CCPDocumentInformation_Type 
  
   getDocumentText()   - 
Method in class edu.uchsc.ccp.util.nlp.document. GenericDocument 
  
   getEntities(String, int, String)   - 
Method in class edu.uchsc.ccp.util.external.abner. Abner_Util 
 Retrieve the entities extracted by ABNER from text as instances of  edu.uchsc.ccp.util.nlp.annotation.TextAnnotation .
   getEntitiesFromFile(String)   - 
Method in class edu.uchsc.ccp.util.external.abgene. AbGeneEISystem 
 Works as it would when using AbGene from the command line.
   getEntitiesFromFile(String)   - 
Method in class edu.uchsc.ccp.util.external.kex. KeXEISystem 
 Works as it would when using KeX from the command line.
   getEntitiesFromFile(String)   - 
Method in interface edu.uchsc.ccp.util.nlp.ei. EIFileBasedSystem 
  
   getEntitiesFromString(String, String)   - 
Method in class edu.uchsc.ccp.util.external.abgene. AbGeneEISystem 
 Take a String as input, create a temporary input file, then run AbGene just as you would from the command line.
   getEntitiesFromString(String, String)   - 
Method in class edu.uchsc.ccp.util.external.kex. KeXEISystem 
 Take a String as input, create a temporary input file, then run KeX just as you would from the command line.
   getEntitiesFromString(String, String)   - 
Method in class edu.uchsc.ccp.util.external.penn. PennBioTaggerEISystem 
 Find entities in a particular String of text.
   getEntitiesFromString(String, String)   - 
Method in interface edu.uchsc.ccp.util.nlp.ei. EISystem 
  
   getFalseNegativeCount()   - 
Method in class edu.uchsc.ccp.util.nlp.stats. PRFResult 
  
   getFalsePositiveCount()   - 
Method in class edu.uchsc.ccp.util.nlp.stats. PRFResult 
  
   getFirstName()   - 
Method in class edu.uchsc.ccp.uima.annotation. CCPAnnotator 
 getter for firstName - gets The first name of the annotator.
   getFirstName(int)   - 
Method in class edu.uchsc.ccp.uima.annotation. CCPAnnotator_Type 
  
   getFirstName()   - 
Method in class edu.uchsc.ccp.util.nlp.annotation. Annotator 
  
   getFirstSlotMention()   - 
Method in class edu.uchsc.ccp.util.nlp.mention. ClassMention 
 Return the first slot mention
   getFirstSlotValue(CCPNonComplexSlotMention)   - 
Static method in class edu.uchsc.ccp.uima.util. UIMA_Util 
  
   getFirstSlotValue()   - 
Method in class edu.uchsc.ccp.util.nlp.mention. SlotMention 
 Return the first slot value
   getFmeasure()   - 
Method in class edu.uchsc.ccp.util.nlp.stats. PRFResult 
  
   getFNAnnotations()   - 
Method in class edu.uchsc.ccp.util.nlp.stats. PRFResult 
 Retrieve the annotations responsible for the false negative counts
   getFPAnnotations()   - 
Method in class edu.uchsc.ccp.util.nlp.stats. PRFResult 
 Retrieve the annotations responsible for the false positive counts
   getFSGenerator()   - 
Method in class edu.uchsc.ccp.uima.annotation. CCPAnnotation_Type 
  
   getFSGenerator()   - 
Method in class edu.uchsc.ccp.uima.annotation. CCPAnnotationSet_Type 
  
   getFSGenerator()   - 
Method in class edu.uchsc.ccp.uima.annotation. CCPAnnotator_Type 
  
   getFSGenerator()   - 
Method in class edu.uchsc.ccp.uima.annotation. CCPDocumentAnnotation_Type 
  
   getFSGenerator()   - 
Method in class edu.uchsc.ccp.uima.annotation. CCPDocumentInformation_Type 
  
   getFSGenerator()   - 
Method in class edu.uchsc.ccp.uima.annotation. CCPDocumentSection_Type 
  
   getFSGenerator()   - 
Method in class edu.uchsc.ccp.uima.annotation. CCPDocumentSubSection_Type 
  
   getFSGenerator()   - 
Method in class edu.uchsc.ccp.uima.annotation. CCPSemanticAnnotation_Type 
  
   getFSGenerator()   - 
Method in class edu.uchsc.ccp.uima.annotation. CCPSpan_Type 
  
   getFSGenerator()   - 
Method in class edu.uchsc.ccp.uima.annotation. CCPSyntacticAnnotation_Type 
  
   getFSGenerator()   - 
Method in class edu.uchsc.ccp.uima.annotation. CCPTextAnnotation_Type 
  
   getFSGenerator()   - 
Method in class edu.uchsc.ccp.uima.annotation.syntactic. CCPClauseAnnotation_Type 
  
   getFSGenerator()   - 
Method in class edu.uchsc.ccp.uima.annotation.syntactic. CCPPhraseAnnotation_Type 
  
   getFSGenerator()   - 
Method in class edu.uchsc.ccp.uima.annotation.syntactic. CCPSentenceAnnotation_Type 
  
   getFSGenerator()   - 
Method in class edu.uchsc.ccp.uima.annotation.syntactic. CCPTokenAnnotation_Type 
  
   getFSGenerator()   - 
Method in class edu.uchsc.ccp.uima.annotation.syntactic. CCPTypedDependency_Type 
  
   getFSGenerator()   - 
Method in class edu.uchsc.ccp.uima.mention. CCPClassMention_Type 
  
   getFSGenerator()   - 
Method in class edu.uchsc.ccp.uima.mention. CCPComplexSlotMention_Type 
  
   getFSGenerator()   - 
Method in class edu.uchsc.ccp.uima.mention. CCPMention_Type 
  
   getFSGenerator()   - 
Method in class edu.uchsc.ccp.uima.mention. CCPNonComplexSlotMention_Type 
  
   getFSGenerator()   - 
Method in class edu.uchsc.ccp.uima.mention. CCPSlotMention_Type 
  
   getGeniaEntitiesFromParse()   - 
Static method in class edu.uchsc.ccp.util.external.lingpipe. LingPipe_Util 
 Returns a mapping between sentence text and entity sets from the previously parsed Genia input text.
   getGeniaSentencesFromParse()   - 
Static method in class edu.uchsc.ccp.util.external.lingpipe. LingPipe_Util 
 Returns a list of sentences that were extracted from the previously parsed Genia input text
   getGovernerTokenNum()   - 
Method in class edu.uchsc.ccp.util.external.stanfordparser. TypedDependencyCls 
  
   getGovernorTokenNum()   - 
Method in class edu.uchsc.ccp.uima.annotation.syntactic. CCPTypedDependency 
 getter for governorTokenNum - gets
   getGovernorTokenNum(int)   - 
Method in class edu.uchsc.ccp.uima.annotation.syntactic. CCPTypedDependency_Type 
  
   getGroupID()   - 
Method in class edu.uchsc.ccp.uima.cc.summary.comparison. AnnotationGroup 
  
   getHashKey()   - 
Method in class edu.uchsc.ccp.util.nlp.annotation. TextAnnotation 
  Deprecated.  
   getHashKey()   - 
Method in class edu.uchsc.ccp.util.nlp.mention. ClassMention 
  
   getHashKey()   - 
Method in class edu.uchsc.ccp.util.nlp.mention. ComplexSlotMention 
  
   getHashKey()   - 
Method in class edu.uchsc.ccp.util.nlp.mention. SlotMention 
  Deprecated.  
   getId()   - 
Method in class edu.uchsc.ccp.uima.cc.summary.comparison. AnnotationGroupID 
  
   getInputMentionReferenceType(String)   - 
Method in class edu.uchsc.ccp.uima.util.opendmap. AnnotationMap 
 Get the type of DMAP Protege Frame ti use for the input token corresponding
 to this type of CAS annotation mention.
   getInputMentionSlotReferenceType(String, String)   - 
Method in class edu.uchsc.ccp.uima.util.opendmap. AnnotationMap 
 Get the slot name to use in the DMAP reference built to represent this CAS
 annotation mention slot.
   getKey()   - 
Method in class edu.uchsc.ccp.util.nlp. KeyValuePair 
 Returns the key
   getLastName()   - 
Method in class edu.uchsc.ccp.uima.annotation. CCPAnnotator 
 getter for lastName - gets The last name of the annotator.
   getLastName(int)   - 
Method in class edu.uchsc.ccp.uima.annotation. CCPAnnotator_Type 
  
   getLastName()   - 
Method in class edu.uchsc.ccp.util.nlp.annotation. Annotator 
  
   getLemma()   - 
Method in class edu.uchsc.ccp.uima.annotation.syntactic. CCPTokenAnnotation 
 getter for lemma - gets The token lemma.
   getLemma(int)   - 
Method in class edu.uchsc.ccp.uima.annotation.syntactic. CCPTokenAnnotation_Type 
  
   getListOfBioIEPMIDs(String)   - 
Static method in class edu.uchsc.ccp.util.nlp.parser.corpus. BioIECorpusParser 
 output a list of the pmids in the BioIE collection
   getMentionName()   - 
Method in class edu.uchsc.ccp.uima.mention. CCPMention 
 getter for mentionName - gets The name of this mention.
   getMentionName(int)   - 
Method in class edu.uchsc.ccp.uima.mention. CCPMention_Type 
  
   getMentionName()   - 
Method in class edu.uchsc.ccp.util.nlp.mention. Mention 
  
   getMultipleSlotMentionsByName(CCPTextAnnotation, String)   - 
Static method in class edu.uchsc.ccp.uima.util. UIMA_Util 
  
   getMultipleSlotMentionsByName(CCPClassMention, String)   - 
Static method in class edu.uchsc.ccp.uima.util. UIMA_Util 
  
   getNextDocumentID()   - 
Method in class edu.uchsc.ccp.util.nlp.parser.corpus. GeniaCorpusParser 
 Returns the document ID of the document that has just been parsed
   getNextDocumentSentences()   - 
Method in class edu.uchsc.ccp.util.nlp.parser.corpus. GeniaCorpusParser 
 Returns a list of sentences from the document that has just been parsed.
   getNextDocumentSentences2EntitiesMap()   - 
Method in class edu.uchsc.ccp.util.nlp.parser.corpus. GeniaCorpusParser 
 Returns a mapping from sentences to a list of entities.
   getNumberOfSpans()   - 
Method in class edu.uchsc.ccp.uima.annotation. CCPTextAnnotation 
 getter for numberOfSpans - gets The number of spans comprising this annotation.
   getNumberOfSpans(int)   - 
Method in class edu.uchsc.ccp.uima.annotation. CCPTextAnnotation_Type 
  
   getOtherDocumentIDs()   - 
Method in class edu.uchsc.ccp.util.nlp.document. GenericDocument 
 Retrieve any other identifiers associated with this document
   getOutputFilename()   - 
Method in class edu.uchsc.ccp.util.external.abgene. AbGeneEISystem 
 Return the outputFileName and path
   getOutputFilename()   - 
Method in class edu.uchsc.ccp.util.external.kex. KeXEISystem 
 Return the outputFileName and path
   getOutputFilename()   - 
Method in interface edu.uchsc.ccp.util.nlp.ei. EIFileBasedSystem 
  
   getOutputReferenceMentionType(Reference)   - 
Method in class edu.uchsc.ccp.uima.util.opendmap. AnnotationMap 
 Get the type of CAS annotation mention that should be created to
 represent this DMAP reference.
   getOutputReferenceMentionType(String)   - 
Method in class edu.uchsc.ccp.uima.util.opendmap. AnnotationMap 
 Get the type of CAS annotation mention that should be created to
 represent this DMAP reference type.
   getOutputReferenceRoot(Reference)   - 
Method in class edu.uchsc.ccp.uima.util.opendmap. AnnotationMap 
 Get the slot filler designated as the root for this reference.
   getOutputReferenceRootSlot(String)   - 
Method in class edu.uchsc.ccp.uima.util.opendmap. AnnotationMap 
 Get the name of the slot holding the root reference for references of
 this DMAP type.
   getOutputReferenceSlotMentionType(Reference, DMAPItem)   - 
Method in class edu.uchsc.ccp.uima.util.opendmap. AnnotationMap 
 Get the name to use for this reference slot in the CAS annotation mention
 being written back to the CAS.
   getOverlappingAnnotations(CCPTextAnnotation, JCas, int)   - 
Static method in class edu.uchsc.ccp.uima.util. UIMA_Util 
  
   getPartOfSpeech()   - 
Method in class edu.uchsc.ccp.uima.annotation.syntactic. CCPTokenAnnotation 
 getter for partOfSpeech - gets The part of speech symbol is stored here.
   getPartOfSpeech(int)   - 
Method in class edu.uchsc.ccp.uima.annotation.syntactic. CCPTokenAnnotation_Type 
  
   getPennTreebankTagLabelLookup()   - 
Static method in class edu.uchsc.ccp.util.nlp. GenericTextUtils 
 Returns a HashMap containing the linguistic annotation tags used by the Penn Treebank, and their corresponding clause, phrase, or token labels. 
 Key = Tag, e.g.
   getPhraseType()   - 
Method in class edu.uchsc.ccp.uima.annotation.syntactic. CCPPhraseAnnotation 
 getter for phraseType - gets The phrase type symbol (e.g.
   getPhraseType(int)   - 
Method in class edu.uchsc.ccp.uima.annotation.syntactic. CCPPhraseAnnotation_Type 
  
   getPrecedingAnnotations(int, int, JCas)   - 
Static method in class edu.uchsc.ccp.uima.util. UIMA_Util 
  
   getPrecision()   - 
Method in class edu.uchsc.ccp.util.nlp.stats. PRFResult 
  
   getRecall()   - 
Method in class edu.uchsc.ccp.util.nlp.stats. PRFResult 
  
   getRecognizedReferences(Parser, Collection<String>, boolean)   - 
Static method in class edu.uchsc.ccp.uima.util.opendmap. ReferenceProcessor 
 Get the desired references ordered by score.
   getReferencesOfInterest()   - 
Method in class edu.uchsc.ccp.uima.util.opendmap. AnnotationMap 
 Get all of the types of the references of interest.
   getRelation()   - 
Method in class edu.uchsc.ccp.uima.annotation.syntactic. CCPTypedDependency 
 getter for relation - gets
   getRelation(int)   - 
Method in class edu.uchsc.ccp.uima.annotation.syntactic. CCPTypedDependency_Type 
  
   getRelation()   - 
Method in class edu.uchsc.ccp.util.external.stanfordparser. TypedDependencyCls 
  
   getSecondaryDocumentIDs()   - 
Method in class edu.uchsc.ccp.uima.annotation. CCPDocumentInformation 
 getter for secondaryDocumentIDs - gets This StringArray is used for secondary document ID storage.
   getSecondaryDocumentIDs(int)   - 
Method in class edu.uchsc.ccp.uima.annotation. CCPDocumentInformation 
 indexed getter for secondaryDocumentIDs - gets an indexed value - This StringArray is used for secondary document ID storage.
   getSecondaryDocumentIDs(int)   - 
Method in class edu.uchsc.ccp.uima.annotation. CCPDocumentInformation_Type 
  
   getSecondaryDocumentIDs(int, int)   - 
Method in class edu.uchsc.ccp.uima.annotation. CCPDocumentInformation_Type 
  
   getSecondaryDocumentIDs()   - 
Method in class edu.uchsc.ccp.util.nlp.document. GenericDocument 
  Deprecated.  
   getSectionEndIndex()   - 
Method in class edu.uchsc.ccp.util.nlp.document. DocumentSection 
  
   getSectionID()   - 
Method in class edu.uchsc.ccp.uima.annotation. CCPDocumentSection 
 getter for sectionID - gets The section ID for this document section, e.g.
   getSectionID(int)   - 
Method in class edu.uchsc.ccp.uima.annotation. CCPDocumentSection_Type 
  
   getSectionIDNameHash()   - 
Static method in class edu.uchsc.ccp.util.nlp.document. DocumentUtil 
  
   getSectionLength()   - 
Method in class edu.uchsc.ccp.uima.annotation. CCPDocumentSection 
 getter for sectionLength - gets The length of this document section (in characters).
   getSectionLength(int)   - 
Method in class edu.uchsc.ccp.uima.annotation. CCPDocumentSection_Type 
  
   getSectionName()   - 
Method in class edu.uchsc.ccp.uima.annotation. CCPDocumentSection 
 getter for sectionName - gets The name of this document section, e.g.
   getSectionName(int)   - 
Method in class edu.uchsc.ccp.uima.annotation. CCPDocumentSection_Type 
  
   getSectionName()   - 
Method in class edu.uchsc.ccp.uima.annotation. CCPDocumentSubSection 
 getter for sectionName - gets The name of the document subsection.
   getSectionName(int)   - 
Method in class edu.uchsc.ccp.uima.annotation. CCPDocumentSubSection_Type 
  
   getSectionNameIDHash()   - 
Static method in class edu.uchsc.ccp.util.nlp.document. DocumentUtil 
  
   getSectionStartIndex()   - 
Method in class edu.uchsc.ccp.util.nlp.document. DocumentSection 
  
   getSetFromString(String)   - 
Static method in class edu.uchsc.ccp.util.nlp. GenericTextUtils 
 This method performs the reverse of the Set.toString() method.
   getSingleLineRepresentation(boolean, boolean)   - 
Method in class edu.uchsc.ccp.util.nlp.annotation. TextAnnotation 
 return a String which represents this annotation, including all class mentions and any associated annotations
 
 NOTE: ANY CHANGES HERE MUST BE MIRRORED IN Knowtator_Util.getSingleLineRepresentationOfAnnotation()
 
 and probably UIMA_Util
   getSingleLineRepresentation()   - 
Method in class edu.uchsc.ccp.util.nlp.mention. ClassMention 
 outputs a single line representation of this class mention.
   getSingleLineRepresentation()   - 
Method in class edu.uchsc.ccp.util.nlp.mention. ComplexSlotMention 
 Return a string representation of this ComplexSlotMention in a single line. 
 Representation: " [mentionName]: [single-line-rep of ta linked to class mention]"
   getSingleLineRepresentation()   - 
Method in class edu.uchsc.ccp.util.nlp.mention. Mention 
  
   getSingleLineRepresentation()   - 
Method in class edu.uchsc.ccp.util.nlp.mention. SlotMention 
 Return a string representation of this SlotMention in a single line. 
 Representation: " [mentionName]:value1:value2:value3..."
   getSingleLineRepresentationOfCCPClassMention(CCPClassMention)   - 
Method in class edu.uchsc.ccp.uima.util. UIMA_Util 
  
   getSingleLineRepresentationOfCCPComplexSlotMention(CCPComplexSlotMention)   - 
Method in class edu.uchsc.ccp.uima.util. UIMA_Util 
  
   getSingleLineRepresentationOfCCPNonComplexSlotMention(CCPNonComplexSlotMention)   - 
Method in class edu.uchsc.ccp.uima.util. UIMA_Util 
  
   getSingleLineRepresentationOfCCPTextAnnotation(CCPTextAnnotation, boolean)   - 
Method in class edu.uchsc.ccp.uima.util. UIMA_Util 
  
   getSlotMentionByName(CCPTextAnnotation, String)   - 
Static method in class edu.uchsc.ccp.uima.util. UIMA_Util 
  
   getSlotMentionByName(CCPClassMention, String)   - 
Static method in class edu.uchsc.ccp.uima.util. UIMA_Util 
  
   getSlotMentionByName(String)   - 
Method in class edu.uchsc.ccp.util.nlp.mention. ClassMention 
  Deprecated.  
   getSlotMentionNames()   - 
Method in class edu.uchsc.ccp.util.nlp.mention. ClassMention 
 Returns the unique names of all slotmentions
   getSlotMentions()   - 
Method in class edu.uchsc.ccp.uima.mention. CCPClassMention 
 getter for slotMentions - gets A class mention optionally has slot mentions which represent attributes of that class.
   getSlotMentions(int)   - 
Method in class edu.uchsc.ccp.uima.mention. CCPClassMention 
 indexed getter for slotMentions - gets an indexed value - A class mention optionally has slot mentions which represent attributes of that class.
   getSlotMentions(int)   - 
Method in class edu.uchsc.ccp.uima.mention. CCPClassMention_Type 
  
   getSlotMentions(int, int)   - 
Method in class edu.uchsc.ccp.uima.mention. CCPClassMention_Type 
  
   getSlotMentions()   - 
Method in class edu.uchsc.ccp.util.nlp.mention. ClassMention 
  
   getSlotMentionsByName(String)   - 
Method in class edu.uchsc.ccp.util.nlp.mention. ClassMention 
 Return all slot mentions for a given name.
   getSlotValues()   - 
Method in class edu.uchsc.ccp.uima.mention. CCPNonComplexSlotMention 
 getter for slotValues - gets Slot fillers (values) for this non-complex slot mention.
   getSlotValues(int)   - 
Method in class edu.uchsc.ccp.uima.mention. CCPNonComplexSlotMention 
 indexed getter for slotValues - gets an indexed value - Slot fillers (values) for this non-complex slot mention.
   getSlotValues(int)   - 
Method in class edu.uchsc.ccp.uima.mention. CCPNonComplexSlotMention_Type 
  
   getSlotValues(int, int)   - 
Method in class edu.uchsc.ccp.uima.mention. CCPNonComplexSlotMention_Type 
  
   getSlotValues()   - 
Method in class edu.uchsc.ccp.util.nlp.mention. SlotMention 
 Get the slot values
   getSlotValuesByName(TextAnnotation, String)   - 
Static method in class edu.uchsc.ccp.util.nlp.annotation. Annotation_Util 
  
   getSpanEnd()   - 
Method in class edu.uchsc.ccp.uima.annotation. CCPSpan 
 getter for spanEnd - gets The character offset for the end of the span.
   getSpanEnd(int)   - 
Method in class edu.uchsc.ccp.uima.annotation. CCPSpan_Type 
  
   getSpanEnd()   - 
Method in class edu.uchsc.ccp.util.nlp.annotation. Span 
  
   getSpans()   - 
Method in class edu.uchsc.ccp.uima.annotation. CCPTextAnnotation 
 getter for spans - gets This FSArray stores the CCPSpans which comprise this annotation.
   getSpans(int)   - 
Method in class edu.uchsc.ccp.uima.annotation. CCPTextAnnotation 
 indexed getter for spans - gets an indexed value - This FSArray stores the CCPSpans which comprise this annotation.
   getSpans(int)   - 
Method in class edu.uchsc.ccp.uima.annotation. CCPTextAnnotation_Type 
  
   getSpans(int, int)   - 
Method in class edu.uchsc.ccp.uima.annotation. CCPTextAnnotation_Type 
  
   getSpans()   - 
Method in class edu.uchsc.ccp.util.nlp.annotation. TextAnnotation 
 Get the ordered list of spans for this TextAnnotation
   getSpanStart()   - 
Method in class edu.uchsc.ccp.uima.annotation. CCPSpan 
 getter for spanStart - gets The character offset for the start of the span.
   getSpanStart(int)   - 
Method in class edu.uchsc.ccp.uima.annotation. CCPSpan_Type 
  
   getSpanStart()   - 
Method in class edu.uchsc.ccp.util.nlp.annotation. Span 
  
   getStem()   - 
Method in class edu.uchsc.ccp.uima.annotation.syntactic. CCPTokenAnnotation 
 getter for stem - gets The token stem.
   getStem(int)   - 
Method in class edu.uchsc.ccp.uima.annotation.syntactic. CCPTokenAnnotation_Type 
  
   getStorageLine()   - 
Method in class edu.uchsc.ccp.util.nlp.annotation. AnnotationSet 
  
   getStorageLine()   - 
Method in class edu.uchsc.ccp.util.nlp.annotation. Annotator 
  
   getSubSections()   - 
Method in class edu.uchsc.ccp.uima.annotation. CCPDocumentSection 
 getter for subSections - gets Allows for direct linking to CCPDocumentSubSections residing in this document section.
   getSubSections(int)   - 
Method in class edu.uchsc.ccp.uima.annotation. CCPDocumentSection 
 indexed getter for subSections - gets an indexed value - Allows for direct linking to CCPDocumentSubSections residing in this document section.
   getSubSections(int)   - 
Method in class edu.uchsc.ccp.uima.annotation. CCPDocumentSection_Type 
  
   getSubSections(int, int)   - 
Method in class edu.uchsc.ccp.uima.annotation. CCPDocumentSection_Type 
  
   getTextAnnotations()   - 
Method in class edu.uchsc.ccp.util.nlp.mention. ClassMention 
  
   getTextAnnotations()   - 
Static method in class edu.uchsc.ccp.util.nlp.parser.corpus. BioIECorpusParser 
  
   getTextAnnotations()   - 
Static method in class edu.uchsc.ccp.util.nlp.parser.corpus. IProLinkParser 
  
   getTextAnnotations()   - 
Static method in class edu.uchsc.ccp.util.nlp.parser.corpus. TexasCorpusParser 
  
   getTextAnnotationsFromJCas(JCas)   - 
Method in class edu.uchsc.ccp.uima.util. UIMA_Util 
  
   getTitle()   - 
Method in class edu.uchsc.ccp.util.nlp.stats. PRFResult 
  
   getTitleText()   - 
Method in class edu.uchsc.ccp.util.nlp.document. GenericDocument 
  Deprecated.  
   getTokenNumber()   - 
Method in class edu.uchsc.ccp.uima.annotation.syntactic. CCPTokenAnnotation 
 getter for tokenNumber - gets The token number indicates the placement of the token within the document text.
   getTokenNumber(int)   - 
Method in class edu.uchsc.ccp.uima.annotation.syntactic. CCPTokenAnnotation_Type 
  
   getTokensAfter(int, int, String)   - 
Static method in class edu.uchsc.ccp.util.nlp. GenericTextUtils 
  
   getTokensBefore(int, int, String)   - 
Static method in class edu.uchsc.ccp.util.nlp. GenericTextUtils 
  
   getTokensFromString(String, String)   - 
Method in class edu.uchsc.ccp.util.external.penn. PennBioTokenizer_Util 
 return a list of TextAnnotations.
   getTokensFromString(String, String)   - 
Method in interface edu.uchsc.ccp.util.nlp.tokenizer. Tokenizer 
 Given a  String  of input text, and a documentID, return tokens in the form of  TextAnnotation  objects.
   getTPAnnotations()   - 
Method in class edu.uchsc.ccp.util.nlp.stats. PRFResult 
 Retrieve the annotations responsible for the true positive counts
   getTruePositiveCount()   - 
Method in class edu.uchsc.ccp.util.nlp.stats. PRFResult 
  
   getTypedDependencies()   - 
Method in class edu.uchsc.ccp.uima.annotation.syntactic. CCPTokenAnnotation 
 getter for typedDependencies - gets If this token is known to be involved in any typed dependency structures, then they are linked through this FSArray.
   getTypedDependencies(int)   - 
Method in class edu.uchsc.ccp.uima.annotation.syntactic. CCPTokenAnnotation 
 indexed getter for typedDependencies - gets an indexed value - If this token is known to be involved in any typed dependency structures, then they are linked through this FSArray.
   getTypedDependencies(int)   - 
Method in class edu.uchsc.ccp.uima.annotation.syntactic. CCPTokenAnnotation_Type 
  
   getTypedDependencies(int, int)   - 
Method in class edu.uchsc.ccp.uima.annotation.syntactic. CCPTokenAnnotation_Type 
  
   getTypeIndexID()   - 
Method in class edu.uchsc.ccp.uima.annotation. CCPAnnotation 
  
   getTypeIndexID()   - 
Method in class edu.uchsc.ccp.uima.annotation. CCPAnnotationSet 
  
   getTypeIndexID()   - 
Method in class edu.uchsc.ccp.uima.annotation. CCPAnnotator 
  
   getTypeIndexID()   - 
Method in class edu.uchsc.ccp.uima.annotation. CCPDocumentAnnotation 
  
   getTypeIndexID()   - 
Method in class edu.uchsc.ccp.uima.annotation. CCPDocumentInformation 
  
   getTypeIndexID()   - 
Method in class edu.uchsc.ccp.uima.annotation. CCPDocumentSection 
  
   getTypeIndexID()   - 
Method in class edu.uchsc.ccp.uima.annotation. CCPDocumentSubSection 
  
   getTypeIndexID()   - 
Method in class edu.uchsc.ccp.uima.annotation. CCPSemanticAnnotation 
  
   getTypeIndexID()   - 
Method in class edu.uchsc.ccp.uima.annotation. CCPSpan 
  
   getTypeIndexID()   - 
Method in class edu.uchsc.ccp.uima.annotation. CCPSyntacticAnnotation 
  
   getTypeIndexID()   - 
Method in class edu.uchsc.ccp.uima.annotation. CCPTextAnnotation 
  
   getTypeIndexID()   - 
Method in class edu.uchsc.ccp.uima.annotation.syntactic. CCPClauseAnnotation 
  
   getTypeIndexID()   - 
Method in class edu.uchsc.ccp.uima.annotation.syntactic. CCPPhraseAnnotation 
  
   getTypeIndexID()   - 
Method in class edu.uchsc.ccp.uima.annotation.syntactic. CCPSentenceAnnotation 
  
   getTypeIndexID()   - 
Method in class edu.uchsc.ccp.uima.annotation.syntactic. CCPTokenAnnotation 
  
   getTypeIndexID()   - 
Method in class edu.uchsc.ccp.uima.annotation.syntactic. CCPTypedDependency 
  
   getTypeIndexID()   - 
Method in class edu.uchsc.ccp.uima.mention. CCPClassMention 
  
   getTypeIndexID()   - 
Method in class edu.uchsc.ccp.uima.mention. CCPComplexSlotMention 
  
   getTypeIndexID()   - 
Method in class edu.uchsc.ccp.uima.mention. CCPMention 
  
   getTypeIndexID()   - 
Method in class edu.uchsc.ccp.uima.mention. CCPNonComplexSlotMention 
  
   getTypeIndexID()   - 
Method in class edu.uchsc.ccp.uima.mention. CCPSlotMention 
  
   getValue()   - 
Method in class edu.uchsc.ccp.util.nlp. KeyValuePair 
 Returns the value
   GLUTAMATE   - 
Static variable in interface edu.uchsc.ccp.util.nlp.mention. ClassMentionTypes 
  
   GLUTAMINE   - 
Static variable in interface edu.uchsc.ccp.util.nlp.mention. ClassMentionTypes 
  
   GLYCINE   - 
Static variable in interface edu.uchsc.ccp.util.nlp.mention. ClassMentionTypes 
  
 
 
    
 H  
 
   handles(String)   - 
Method in class edu.uchsc.ccp.uima.util.opendmap. DMAPStanfordPhraseGraph 
 Check whether this property function handler wants to handle the specified function.
   hasEquivalentMentionNames(Mention, Mention)   - 
Method in class edu.uchsc.ccp.util.nlp.mention.comparison. IdenticalMentionComparator 
 Compare the mention names for the two mentions.
   hasEquivalentMentionNames(Mention, Mention)   - 
Method in class edu.uchsc.ccp.util.nlp.mention.comparison. MentionComparator 
  
   hashCode()   - 
Method in class edu.uchsc.ccp.uima.cc.summary.comparison. AnnotationGroupID 
  
   hashCode()   - 
Method in class edu.uchsc.ccp.util.nlp.annotation. AnnotationSet 
  
   hashCode()   - 
Method in class edu.uchsc.ccp.util.nlp.annotation. Annotator 
  
   hashCode()   - 
Method in class edu.uchsc.ccp.util.nlp.annotation. Span 
  
   hashCode()   - 
Method in class edu.uchsc.ccp.util.nlp.annotation. TextAnnotation 
  
   hashCode()   - 
Method in class edu.uchsc.ccp.util.nlp.mention. ComplexSlotMention 
  
   hashCode()   - 
Method in class edu.uchsc.ccp.util.nlp.mention. SlotMention 
  
   hasInputMentionsOfInterest()   - 
Method in class edu.uchsc.ccp.uima.util.opendmap. AnnotationMap 
 Check whether any CAS annotations other than TokenAnnotations are to be 
 used as input tokens (typically named entities).
   hasNext()   - 
Method in class edu.uchsc.ccp.uima.util.opendmap. DMAPJcasTokenizer 
 Check if there are any more tokens to process
   hasNext(String)   - 
Method in class edu.uchsc.ccp.util.external.geniatagger. GeniaTaggerOutputFileParser 
  
   hasNext()   - 
Method in class edu.uchsc.ccp.util.nlp.parser.corpus. Bio1CorpusParser 
  
   hasNext()   - 
Method in class edu.uchsc.ccp.util.nlp.parser.corpus. BioIECorpusParser 
  
   hasNext()   - 
Method in class edu.uchsc.ccp.util.nlp.parser.corpus. CorpusParser 
  
   hasNext()   - 
Method in class edu.uchsc.ccp.util.nlp.parser.corpus. GeniaCorpusParser 
  
   hasNext()   - 
Method in class edu.uchsc.ccp.util.nlp.parser.corpus. IProLinkCorpus1Parser 
  
   hasNext()   - 
Method in class edu.uchsc.ccp.util.nlp.parser.corpus. IProLinkCorpus2Parser 
  
   hasNext()   - 
Method in class edu.uchsc.ccp.util.nlp.parser.corpus. TexasCorpusParser 
  
   hasNext()   - 
Method in class edu.uchsc.ccp.util.nlp.parser.corpus. YapexCorpusParser 
  
   hasNextDocument()   - 
Method in class edu.uchsc.ccp.util.nlp.parser.corpus. IProLinkCorpus1Parser 
 corpus1.xml has tags: protein, acronym, and compound-protein
   hasNextDocument()   - 
Method in class edu.uchsc.ccp.util.nlp.parser.corpus. IProLinkCorpus2Parser 
 corpus1.xml has tags: protein, acronym, and compound-protein
   hasOutputReferencesOfInterest()   - 
Method in class edu.uchsc.ccp.uima.util.opendmap. AnnotationMap 
 Check whether any DMAP output references are to be written back into the CAS.
   HISTIDINE   - 
Static variable in interface edu.uchsc.ccp.util.nlp.mention. ClassMentionTypes 
  
   HTML_PARAGRAPH   - 
Static variable in interface edu.uchsc.ccp.util.nlp.mention. ClassMentionTypes 
 Document level Class Mentions
   HTML_PARAGRAPH_END   - 
Static variable in interface edu.uchsc.ccp.util.nlp.mention. ClassMentionTypes 
  
   HTML_PARAGRAPH_START   - 
Static variable in interface edu.uchsc.ccp.util.nlp.mention. ClassMentionTypes 
  
   HTML_PARAGRAPH_TAG   - 
Static variable in interface edu.uchsc.ccp.util.nlp.mention. ClassMentionTypes 
  
   HW_ABSTRACT   - 
Static variable in interface edu.uchsc.ccp.util.nlp.document. DocumentSectionTypes 
  
   HW_TITLE   - 
Static variable in interface edu.uchsc.ccp.util.nlp.document. DocumentSectionTypes 
  
 
 
    
 I  
 
   IdenticalClassMentionComparator   - Class in  edu.uchsc.ccp.util.nlp.mention.comparison  This class implements a comparator which compares ClassMentions.   IdenticalClassMentionComparator()   - 
Constructor for class edu.uchsc.ccp.util.nlp.mention.comparison. IdenticalClassMentionComparator 
  
   IdenticalComplexSlotMentionComparator   - Class in  edu.uchsc.ccp.util.nlp.mention.comparison      IdenticalComplexSlotMentionComparator()   - 
Constructor for class edu.uchsc.ccp.util.nlp.mention.comparison. IdenticalComplexSlotMentionComparator 
  
   IdenticalMentionComparator   - Class in  edu.uchsc.ccp.util.nlp.mention.comparison  The IdenticalMentionComparator enforces a strict comparison of mention names.   IdenticalMentionComparator()   - 
Constructor for class edu.uchsc.ccp.util.nlp.mention.comparison. IdenticalMentionComparator 
  
   IdenticalSlotMentionComparator   - Class in  edu.uchsc.ccp.util.nlp.mention.comparison      IdenticalSlotMentionComparator()   - 
Constructor for class edu.uchsc.ccp.util.nlp.mention.comparison. IdenticalSlotMentionComparator 
  
   IgnoreSpanComparator   - Class in  edu.uchsc.ccp.util.nlp.annotation.comparison  Use this span comparator when you do not care to compare spans.   IgnoreSpanComparator()   - 
Constructor for class edu.uchsc.ccp.util.nlp.annotation.comparison. IgnoreSpanComparator 
  
   initialize(AnnotatorContext)   - 
Method in class edu.uchsc.ccp.uima.ae.nlp.ei.gene. AbGene_AE 
  
   initialize(AnnotatorContext)   - 
Method in class edu.uchsc.ccp.uima.ae.nlp.ei.gene. ABNER_AE 
  
   initialize(AnnotatorContext)   - 
Method in class edu.uchsc.ccp.uima.ae.nlp.ei.gene. KeX_AE 
  
   initialize(AnnotatorContext)   - 
Method in class edu.uchsc.ccp.uima.ae.nlp.ei.gene. LingPipeEntityTagger_AE 
  
   initialize(AnnotatorContext)   - 
Method in class edu.uchsc.ccp.uima.ae.nlp.ei.gene. PennBioTagger_AE 
  
   initialize(AnnotatorContext)   - 
Method in class edu.uchsc.ccp.uima.ae.nlp.ei.mutation. MutationFinder_AE 
  
   initialize(AnnotatorContext)   - 
Method in class edu.uchsc.ccp.uima.ae.nlp.opendmap. CCPOpenDMAP_AE 
 Initialize the GeneRIF Annotator.
   initialize(AnnotatorContext)   - 
Method in class edu.uchsc.ccp.uima.ae.nlp.opendmap. OpenDmapAnnotator 
 Initialize the OpenDMAP parser and load the Protege
 project and the pattern files.
   initialize(AnnotatorContext)   - 
Method in class edu.uchsc.ccp.uima.ae.nlp.parser. StanfordParser_AE 
 Initializes the StanfordParser_AE by reading in the input parameters from the descriptor file and initializing a new StanfordParserUtil
   initialize(AnnotatorContext)   - 
Method in class edu.uchsc.ccp.uima.ae.nlp.sentenceDetector. KeXSentenceDetector_AE 
  
   initialize(AnnotatorContext)   - 
Method in class edu.uchsc.ccp.uima.ae.nlp.sentenceDetector. OpenNLPSentenceDetector_AE 
  
   initialize(AnnotatorContext)   - 
Method in class edu.uchsc.ccp.uima.ae.nlp.stemmer. PorterStemmer_AE 
 Initializes the PorterStemmerAnnotator by initializing a new PorterStemmer
   initialize(AnnotatorContext)   - 
Method in class edu.uchsc.ccp.uima.ae.nlp.tokenizer. GeniaTaggerOutputFileLoader_AE 
  
   initialize(AnnotatorContext)   - 
Method in class edu.uchsc.ccp.uima.ae.nlp.tokenizer. LingPipePOSTagger_AE 
 Initializes the StanfordParser_AE by reading in the input parameters from the descriptor file and initializing a new StanfordParserUtil
   initialize(AnnotatorContext)   - 
Method in class edu.uchsc.ccp.uima.ae.nlp.tokenizer. PennBioTokenizer_AE 
 Initializes the StanfordParser_AE by reading in the input parameters from the descriptor file and initializing a new
 StanfordParserUtil
   initialize(AnnotatorContext)   - 
Method in class edu.uchsc.ccp.uima.ae.util. AnnotationFromFileLoader_AE 
  
   initialize(AnnotatorContext)   - 
Method in class edu.uchsc.ccp.uima.ae.util.converter. AnnotationSetOverrider_AE 
  
   initialize(AnnotatorContext)   - 
Method in class edu.uchsc.ccp.uima.ae.util.converter. AnnotatorConverter_AE 
  
   initialize(AnnotatorContext)   - 
Method in class edu.uchsc.ccp.uima.ae.util.converter. AnnotatorOverrider_AE 
  
   initialize(AnnotatorContext)   - 
Method in class edu.uchsc.ccp.uima.ae.util.converter. ClassMentionConverter_AE 
  
   initialize(AnnotatorContext)   - 
Method in class edu.uchsc.ccp.uima.ae.util.converter. DocumentLevelAnnotationCreator_AE 
  
   initialize(AnnotatorContext)   - 
Method in class edu.uchsc.ccp.uima.ae.util.filter. AnnotationConsensusFilter_AE 
  
   initialize(AnnotatorContext)   - 
Method in class edu.uchsc.ccp.uima.ae.util.filter. AnnotationSetFilter_AE 
  
   initialize(AnnotatorContext)   - 
Method in class edu.uchsc.ccp.uima.ae.util.filter. AnnotatorFilter_AE 
  
   initialize(AnnotatorContext)   - 
Method in class edu.uchsc.ccp.uima.ae.util.filter. ClassMentionRemovalFilter_AE 
  
   initialize(AnnotatorContext)   - 
Method in class edu.uchsc.ccp.uima.ae.util.filter. ClassMentionTypeFilter_AE 
  
   initialize(AnnotatorContext)   - 
Method in class edu.uchsc.ccp.uima.ae.util.filter. OverlappingAnnotationResolver_AE 
  
   initialize(AnnotatorContext)   - 
Method in class edu.uchsc.ccp.uima.ae.util.filter. SlotRemovalFilter_AE 
  
   initialize()   - 
Method in class edu.uchsc.ccp.uima.cc.summary. AnnotationPrinter 
 Initializes the CAS Consumer by reading in parameters from the descriptor file.
   initialize()   - 
Method in class edu.uchsc.ccp.uima.cc.summary.comparison. AnnotationComparator_CC 
 Initializes this CAS Consumer with the parameters specified in the descriptor.
   initialize()   - 
Method in class edu.uchsc.ccp.uima.cc.summary.opendmap. TestPrinter 
 The standard UIMA initialize method for this processor.
   initializeEntityTaggingSystem(String, String)   - 
Static method in class edu.uchsc.ccp.util.external.lingpipe. LingPipe_Util 
 Use with Lingpipe_3.0.0
   initializePOSTaggingSystem(String)   - 
Static method in class edu.uchsc.ccp.util.external.lingpipe. LingPipe_Util 
 Ready the system for POS tagging.
   INSERTION   - 
Static variable in interface edu.uchsc.ccp.util.nlp.mention. ClassMentionTypes 
  
   INSERTION_INSERTED_ELEMENT   - 
Static variable in interface edu.uchsc.ccp.util.nlp.mention. ComplexSlotMentionTypes 
  
   INSERTION_INSERTION_START   - 
Static variable in interface edu.uchsc.ccp.util.nlp.mention. ComplexSlotMentionTypes 
  
   InvalidSpanException   - Exception in  edu.uchsc.ccp.util.nlp.annotation      InvalidSpanException(String)   - 
Constructor for exception edu.uchsc.ccp.util.nlp.annotation. InvalidSpanException 
  
   InvalidTypedDependencyException   - Exception in  edu.uchsc.ccp.util.external.stanfordparser      InvalidTypedDependencyException()   - 
Constructor for exception edu.uchsc.ccp.util.external.stanfordparser. InvalidTypedDependencyException 
  
   IProLinkCorpus1Parser   - Class in  edu.uchsc.ccp.util.nlp.parser.corpus  A parser for the iProLink corpus1.xml file.   IProLinkCorpus1Parser(String)   - 
Constructor for class edu.uchsc.ccp.util.nlp.parser.corpus. IProLinkCorpus1Parser 
  
   IProLinkCorpus2Parser   - Class in  edu.uchsc.ccp.util.nlp.parser.corpus  A parser for the iProLink corpus2.xml file.   IProLinkCorpus2Parser(String)   - 
Constructor for class edu.uchsc.ccp.util.nlp.parser.corpus. IProLinkCorpus2Parser 
  
   IProLinkParser   - Class in  edu.uchsc.ccp.util.nlp.parser.corpus  This class contains methods to parse the 2 IProLink XML corpora files.   IProLinkParser()   - 
Constructor for class edu.uchsc.ccp.util.nlp.parser.corpus. IProLinkParser 
  
   isGoldStandard()   - 
Method in class edu.uchsc.ccp.uima.cc.summary.comparison. ComparisonGroup 
  
   isInputMentionOfInterest(String)   - 
Method in class edu.uchsc.ccp.uima.util.opendmap. AnnotationMap 
 Check whether a specific CAS annotation mention is to become an input
 token.
   isInputMentionSlotIncluded(String, String)   - 
Method in class edu.uchsc.ccp.uima.util.opendmap. AnnotationMap 
 Check whether a particular slot of a CAS annotation mention should be included
 in the DMAP Protege Frame used for the input token.
   isMemberOfAnnotationSet(int)   - 
Method in class edu.uchsc.ccp.util.nlp.annotation. TextAnnotation 
 Return true if this TextAnnotation is a member of the inputted annotation set, false otherwise.
   isNumber(String)   - 
Static method in class edu.uchsc.ccp.util.nlp. GenericTextUtils 
  
   ISOLEUCINE   - 
Static variable in interface edu.uchsc.ccp.util.nlp.mention. ClassMentionTypes 
  
   isOutputReferenceOfInterest(String)   - 
Method in class edu.uchsc.ccp.uima.util.opendmap. AnnotationMap 
 Check whether a specific DMAP output reference type should be written
 back into the CAS.
   isOutputReferenceOfInterest(Reference)   - 
Method in class edu.uchsc.ccp.uima.util.opendmap. AnnotationMap 
 Check whether a specific DMAP output reference should be written
 back into the CAS.
   isOutputReferenceSlotExcluded(String, String)   - 
Method in class edu.uchsc.ccp.uima.util.opendmap. AnnotationMap 
 Check whether an output reference slot should be included in the CAS
 annotation mention being written back to the CAS.
   isOutputReferenceSlotOfInterest(Reference, DMAPItem)   - 
Method in class edu.uchsc.ccp.uima.util.opendmap. AnnotationMap 
 Check whether this reference slot should be included in the
 annotation mention written back to the CAS.
   isStopWord(String)   - 
Static method in class edu.uchsc.ccp.util.nlp. GenericTextUtils 
  
   isValid()   - 
Method in class edu.uchsc.ccp.uima.cc.summary.comparison. AnnotationGroup 
  
   isValid()   - 
Method in class edu.uchsc.ccp.uima.cc.summary.comparison. ComparisonGroup 
  
 
 
    
 K  
 
   keepLongestAnnotation(Map<Integer, CCPTextAnnotation>, Map<Integer, Boolean>, List<CCPTextAnnotation>, JCas)   - 
Method in class edu.uchsc.ccp.uima.ae.util.filter. OverlappingAnnotationResolver_AE 
 keeps the longest annotation for a group of overlapping annotations
   keepLongestAnnotation(List<TextAnnotation>, Map<Integer, TextAnnotation>, Map<Integer, Boolean>, List<TextAnnotation>)   - 
Method in class edu.uchsc.ccp.util.nlp.annotation.filter. OverlappingAnnotationResolver 
 keeps the longest annotation for a group of overlapping annotations
   keepLongestCommonSubstring   - 
Variable in class edu.uchsc.ccp.uima.ae.util.filter. OverlappingAnnotationResolver_AE 
  
   keepLongestCommonSubstring(Map<Integer, CCPTextAnnotation>, Map<Integer, Boolean>, List<CCPTextAnnotation>, JCas)   - 
Method in class edu.uchsc.ccp.uima.ae.util.filter. OverlappingAnnotationResolver_AE 
 keeps the longest common substring for a group of overlapping annotations
   KeX_AE   - Class in  edu.uchsc.ccp.uima.ae.nlp.ei.gene      KeX_AE()   - 
Constructor for class edu.uchsc.ccp.uima.ae.nlp.ei.gene. KeX_AE 
  
   KeXEISystem   - Class in  edu.uchsc.ccp.util.external.kex      KeXEISystem(String)   - 
Constructor for class edu.uchsc.ccp.util.external.kex. KeXEISystem 
 Initialize a new KeX EI System.
   KeXSentenceDetector_AE   - Class in  edu.uchsc.ccp.uima.ae.nlp.sentenceDetector      KeXSentenceDetector_AE()   - 
Constructor for class edu.uchsc.ccp.uima.ae.nlp.sentenceDetector. KeXSentenceDetector_AE 
  
   KeyValuePair   - Class in  edu.uchsc.ccp.util.nlp  This class is a utility class used for interacting with HashMaps, and potentially and application requiring Key/Value pairings.   KeyValuePair(String, int)   - 
Constructor for class edu.uchsc.ccp.util.nlp. KeyValuePair 
 Construct a new KeyValuePair with the given input arguments.
 
 
    
 L  
 
   length()   - 
Method in class edu.uchsc.ccp.util.nlp.annotation. Span 
 Return the length of the span
   length()   - 
Method in class edu.uchsc.ccp.util.nlp.annotation. TextAnnotation 
 Return the length of this TextAnnotation.
   LEUCINE   - 
Static variable in interface edu.uchsc.ccp.util.nlp.mention. ClassMentionTypes 
  
   LingPipe_Util   - Class in  edu.uchsc.ccp.util.external.lingpipe      LingPipe_Util()   - 
Constructor for class edu.uchsc.ccp.util.external.lingpipe. LingPipe_Util 
  
   LingPipeEntityTagger_AE   - Class in  edu.uchsc.ccp.uima.ae.nlp.ei.gene      LingPipeEntityTagger_AE()   - 
Constructor for class edu.uchsc.ccp.uima.ae.nlp.ei.gene. LingPipeEntityTagger_AE 
  
   LingPipePOSTagger_AE   - Class in  edu.uchsc.ccp.uima.ae.nlp.tokenizer  This class uses the LingPipe POS tagger to create CCPTokenAnnotations.   LingPipePOSTagger_AE()   - 
Constructor for class edu.uchsc.ccp.uima.ae.nlp.tokenizer. LingPipePOSTagger_AE 
  
   LingPipeSentenceDetector_AE   - Class in  edu.uchsc.ccp.uima.ae.nlp.sentenceDetector      LingPipeSentenceDetector_AE()   - 
Constructor for class edu.uchsc.ccp.uima.ae.nlp.sentenceDetector. LingPipeSentenceDetector_AE 
  
   LYSINE   - 
Static variable in interface edu.uchsc.ccp.util.nlp.mention. ClassMentionTypes 
  
 
 
    
 M  
 
   MACROMOLECULE   - 
Static variable in interface edu.uchsc.ccp.util.nlp.mention. ClassMentionTypes 
  
   main(String[])   - 
Static method in class edu.uchsc.ccp.util.external.opennlp. OpenNLPSentenceSegmenter 
  
   main(String[])   - 
Static method in class edu.uchsc.ccp.util.external.stanfordparser. StanfordParser_Util 
  
   main(String[])   - 
Static method in class edu.uchsc.ccp.util.nlp.parser.corpus. Bio1CorpusParser 
 To determine overlap in documents with GENIA: 
 args[0] = parse or compare; if parse, the Bio1 corpus is parsed.
   main(String[])   - 
Static method in class edu.uchsc.ccp.util.nlp.parser.corpus. BioIECorpusParser 
 args[0] = BioIE corpus directory (where wordfreak files reside)
   main(String[])   - 
Static method in class edu.uchsc.ccp.util.nlp.parser.corpus. IProLinkParser 
 args[0] = iProLink corpus1 file 
 args[1] = iProLink corpus2 file 
   main(String[])   - 
Static method in class edu.uchsc.ccp.util.nlp.parser.corpus. TexasCorpusParser 
 args[0] = texas corpus directory
   main(String[])   - 
Static method in class edu.uchsc.ccp.util. RunExecutable 
 This method used for testing purposes only.
   makeXMLReader()   - 
Static method in class edu.uchsc.ccp.util.nlp. GenericTextUtils 
 Taken from: http://userpage.fu-berlin.de/~ram/pub/pub_jf47hxhHHt/java_sax_parser_en
   MALIGNANCY   - 
Static variable in interface edu.uchsc.ccp.util.nlp.mention. ClassMentionTypes 
 For Penn Bio Tagger
   matches(Span, Span)   - 
Method in class edu.uchsc.ccp.util.nlp.annotation.comparison. SpanComparator 
 Returns 0 if the spans are equal, -1 if span1 starts before span2, 1 if span1 starts after span2.
   matches(List<Span>, List<Span>)   - 
Method in class edu.uchsc.ccp.util.nlp.annotation.comparison. SpanComparator 
 Compares a list of spans.
   MEDLEE_ID   - 
Static variable in interface edu.uchsc.ccp.util.nlp.mention. SlotMentionTypes 
 for MedLEE
   Mention   - Class in  edu.uchsc.ccp.util.nlp.mention      Mention()   - 
Constructor for class edu.uchsc.ccp.util.nlp.mention. Mention 
  Deprecated.  
   Mention(String)   - 
Constructor for class edu.uchsc.ccp.util.nlp.mention. Mention 
  
   MentionComparator   - Class in  edu.uchsc.ccp.util.nlp.mention.comparison  This is an abstract class for mention comparators.   MentionComparator()   - 
Constructor for class edu.uchsc.ccp.util.nlp.mention.comparison. MentionComparator 
  
   MentionDocumentLevelSortComparator   - Class in  edu.uchsc.ccp.util.nlp.mention  Allows lists of mentions to be sorted by Collections.sort() so that the getSingleLineRepresentation() methods output the same string no matter what
 order the mentions were added   MentionDocumentLevelSortComparator()   - 
Constructor for class edu.uchsc.ccp.util.nlp.mention. MentionDocumentLevelSortComparator 
  
   MentionSortComparator   - Class in  edu.uchsc.ccp.util.nlp.mention  Allows lists of mentions to be sorted by Collections.sort() so that the getSingleLineRepresentation() methods output the same string no matter what
 order the mentions were added   MentionSortComparator()   - 
Constructor for class edu.uchsc.ccp.util.nlp.mention. MentionSortComparator 
  
   MESH_ID   - 
Static variable in interface edu.uchsc.ccp.util.nlp.mention. SlotMentionTypes 
 for Olga's concept recognizers
   metaDataCompare(TextAnnotation, TextAnnotation)   - 
Method in class edu.uchsc.ccp.util.nlp.annotation.comparison. AnnotationComparator 
 Compare the meta data of two annotations (document ID, document collection ID, and span(s)).
   METHIONINE   - 
Static variable in interface edu.uchsc.ccp.util.nlp.mention. ClassMentionTypes 
  
   MULTIPLIER   - 
Variable in class edu.uchsc.ccp.util.nlp.mention.comparison. MentionComparator 
 So that differences found in mentions can be distinguished between differences found in spans, the mention comparators should return -3, 0, or
 3, therefore the MULTIPLIER variable is used to scale the compare() output.
   MUTATION_EVENT   - 
Static variable in interface edu.uchsc.ccp.util.nlp.mention. ClassMentionTypes 
 for Mutation project
   MutationFinder_AE   - Class in  edu.uchsc.ccp.uima.ae.nlp.ei.mutation      MutationFinder_AE()   - 
Constructor for class edu.uchsc.ccp.uima.ae.nlp.ei.mutation. MutationFinder_AE 
  
 
 
    
 N  
 
   NCBI_TAX_ID   - 
Static variable in interface edu.uchsc.ccp.util.nlp.mention. SlotMentionTypes 
 For Species Classification
   next()   - 
Method in class edu.uchsc.ccp.uima.util.opendmap. DMAPJcasTokenizer 
 Grab the next token and return it.
   next()   - 
Method in class edu.uchsc.ccp.util.external.geniatagger. GeniaTaggerOutputFileParser 
  
   next()   - 
Method in class edu.uchsc.ccp.util.nlp.parser.corpus. Bio1CorpusParser 
  
   next()   - 
Method in class edu.uchsc.ccp.util.nlp.parser.corpus. BioIECorpusParser 
  
   next()   - 
Method in class edu.uchsc.ccp.util.nlp.parser.corpus. CorpusParser 
  
   next()   - 
Method in class edu.uchsc.ccp.util.nlp.parser.corpus. GeniaCorpusParser 
  
   next()   - 
Method in class edu.uchsc.ccp.util.nlp.parser.corpus. IProLinkCorpus1Parser 
  
   next()   - 
Method in class edu.uchsc.ccp.util.nlp.parser.corpus. IProLinkCorpus2Parser 
  
   next()   - 
Method in class edu.uchsc.ccp.util.nlp.parser.corpus. TexasCorpusParser 
  
   next()   - 
Method in class edu.uchsc.ccp.util.nlp.parser.corpus. YapexCorpusParser 
  
   NLPStats   - Class in  edu.uchsc.ccp.util.nlp.stats  This class implements some commonly used statistics in the field of NLP, namely precision, recall, F-measure, and the Dice coefficient.   NLPStats()   - 
Constructor for class edu.uchsc.ccp.util.nlp.stats. NLPStats 
  
   NOMINALIZATION   - 
Static variable in interface edu.uchsc.ccp.util.nlp.mention. ClassMentionTypes 
 For Nominalization project
   NOMINALIZATION_ACTIVATION   - 
Static variable in interface edu.uchsc.ccp.util.nlp.mention. ClassMentionTypes 
  
   NOMINALIZATION_DEVELOPMENT   - 
Static variable in interface edu.uchsc.ccp.util.nlp.mention. ClassMentionTypes 
  
   NOMINALIZATION_EXPRESSION   - 
Static variable in interface edu.uchsc.ccp.util.nlp.mention. ClassMentionTypes 
  
   NOMINALIZATION_FUNCTION   - 
Static variable in interface edu.uchsc.ccp.util.nlp.mention. ClassMentionTypes 
  
   NOMINALIZATION_GROWTH   - 
Static variable in interface edu.uchsc.ccp.util.nlp.mention. ClassMentionTypes 
  
   NOMINALIZATION_INHIBITION   - 
Static variable in interface edu.uchsc.ccp.util.nlp.mention. ClassMentionTypes 
  
   NOMINALIZATION_INTERACTION   - 
Static variable in interface edu.uchsc.ccp.util.nlp.mention. ClassMentionTypes 
  
   NOMINALIZATION_MUTATION   - 
Static variable in interface edu.uchsc.ccp.util.nlp.mention. ClassMentionTypes 
  
   NOMINALIZATION_PHOSPHORYLATION   - 
Static variable in interface edu.uchsc.ccp.util.nlp.mention. ClassMentionTypes 
  
   NOMINALIZATION_REGULATION   - 
Static variable in interface edu.uchsc.ccp.util.nlp.mention. ClassMentionTypes 
  
   NOMINALIZATION_RESPONSE   - 
Static variable in interface edu.uchsc.ccp.util.nlp.mention. ClassMentionTypes 
  
   NOMINALIZATION_TRANSCRIPTION   - 
Static variable in interface edu.uchsc.ccp.util.nlp.mention. ClassMentionTypes 
  
   normalizeSyntacticAnnotations(JCas)   - 
Static method in class edu.uchsc.ccp.uima.util. UIMASyntacticAnnotation_Util 
  
   NUCLEIC_ACID   - 
Static variable in interface edu.uchsc.ccp.util.nlp.mention. ClassMentionTypes 
  
 
 
    
 O  
 
   OpenDmapAnnotator   - Class in  edu.uchsc.ccp.uima.ae.nlp.opendmap  This class is a wrapper for OpenDMAP to help fit it into the
 UIMA framework.   OpenDmapAnnotator()   - 
Constructor for class edu.uchsc.ccp.uima.ae.nlp.opendmap. OpenDmapAnnotator 
 Create a new, empty OpenDMAP Annotator.
   OpenNLPSentenceDetector_AE   - Class in  edu.uchsc.ccp.uima.ae.nlp.sentenceDetector      OpenNLPSentenceDetector_AE()   - 
Constructor for class edu.uchsc.ccp.uima.ae.nlp.sentenceDetector. OpenNLPSentenceDetector_AE 
  
   OpenNLPSentenceSegmenter   - Class in  edu.uchsc.ccp.util.external.opennlp      OpenNLPSentenceSegmenter(String)   - 
Constructor for class edu.uchsc.ccp.util.external.opennlp. OpenNLPSentenceSegmenter 
  
   openPrintStream(String)   - 
Static method in class edu.uchsc.ccp.util.nlp. GenericTextUtils 
 Opens a PrintStream for printing to the given output file location
   openPrintStream(String, boolean)   - 
Static method in class edu.uchsc.ccp.util.nlp. GenericTextUtils 
 Opens a PrintStream for printing to the given output file location
   openReader(String)   - 
Static method in class edu.uchsc.ccp.util.nlp. GenericTextUtils 
 Opens a BufferedReader for reading from a text file
   ORGAN   - 
Static variable in interface edu.uchsc.ccp.util.nlp.mention. ClassMentionTypes 
 for Olga's concept recognizers
   ORGANISM   - 
Static variable in interface edu.uchsc.ccp.util.nlp.mention. ClassMentionTypes 
  
   OTHER_ID   - 
Static variable in interface edu.uchsc.ccp.util.nlp.document. DocumentSectionTypes 
  
   OTHER_NAME   - 
Static variable in interface edu.uchsc.ccp.util.nlp.document. DocumentSectionTypes 
  
   outputAllMedlineIDs(String)   - 
Static method in class edu.uchsc.ccp.util.nlp.parser.corpus. GeniaCorpusParser 
  
   OverlappingAnnotationResolver   - Class in  edu.uchsc.ccp.util.nlp.annotation.filter  This class mirrors the functionality of the class by the same name in the edu.uchsc.ccp.uima package   OverlappingAnnotationResolver(String)   - 
Constructor for class edu.uchsc.ccp.util.nlp.annotation.filter. OverlappingAnnotationResolver 
  
   OverlappingAnnotationResolver_AE   - Class in  edu.uchsc.ccp.uima.ae.util.filter  Default behavior is to keep the longest annotation for any that overlap.   OverlappingAnnotationResolver_AE()   - 
Constructor for class edu.uchsc.ccp.uima.ae.util.filter. OverlappingAnnotationResolver_AE 
  
   overlaps(CCPTextAnnotation, CCPTextAnnotation)   - 
Static method in class edu.uchsc.ccp.uima.util. UIMA_Annotation_Util 
  
   overlaps(Span, Span)   - 
Method in class edu.uchsc.ccp.util.nlp.annotation.comparison. SpanComparator 
 Returns 0 if span1 and span2 overlap.
   overlaps(List<Span>, List<Span>)   - 
Method in class edu.uchsc.ccp.util.nlp.annotation.comparison. SpanComparator 
 Returns 0 if the span lists overlap.
   overlaps(Span)   - 
Method in class edu.uchsc.ccp.util.nlp.annotation. Span 
 The overlaps() method uses the SloppySpanComparator
   overlaps(TextAnnotation)   - 
Method in class edu.uchsc.ccp.util.nlp.annotation. TextAnnotation 
 returns true if the total span of this TextAnnotation overlaps with the total span of the input TextAnnotation
 
 
    
 P  
 
   PARAM_ABGENE_BASE_DIRECTORY   - 
Static variable in class edu.uchsc.ccp.uima.ae.nlp.ei.gene. AbGene_AE 
 Name of configuration parameter that must be set to the path of the directory TnT will use for storing files generated in the gene
 annotation process.
   PARAM_ABGENE_BASE_DIRECTORY   - 
Static variable in class edu.uchsc.ccp.uima.ae.nlp.ei.gene. KeX_AE 
 Name of configuration parameter that must be set to the path of the directory TnT will use for storing files generated in the gene
 annotation process.
   PARAM_AFFILIATION   - 
Static variable in class edu.uchsc.ccp.uima.ae.util.converter. AnnotatorConverter_AE 
  
   PARAM_AFFILIATION   - 
Static variable in class edu.uchsc.ccp.uima.ae.util.converter. AnnotatorOverrider_AE 
  
   PARAM_ANNOTATION_FILE   - 
Variable in class edu.uchsc.ccp.uima.ae.util. AnnotationFromFileLoader_AE 
  
   PARAM_ANNOTATION_OUTPUT_FILE   - 
Static variable in class edu.uchsc.ccp.uima.cc.summary.comparison. AnnotationComparator_CC 
 if filled, TP, FP, and FN annotations will be printed to file
   PARAM_ANNOTATION_SETS_TO_IGNORE   - 
Variable in class edu.uchsc.ccp.uima.ae.util.converter. AnnotationSetOverrider_AE 
  
   PARAM_ANNOTATION_SETS_TO_IGNORE   - 
Variable in class edu.uchsc.ccp.uima.ae.util.filter. AnnotationConsensusFilter_AE 
  
   PARAM_ANNOTATION_SETS_TO_IGNORE   - 
Variable in class edu.uchsc.ccp.uima.ae.util.filter. OverlappingAnnotationResolver_AE 
  
   PARAM_ANNOTATION_TYPE_OF_INTEREST   - 
Variable in class edu.uchsc.ccp.uima.ae.util.filter. AnnotationConsensusFilter_AE 
 The annotation type to look at when making consensus selections
   PARAM_ANNOTATION_TYPE_OF_INTEREST   - 
Variable in class edu.uchsc.ccp.uima.ae.util.filter. OverlappingAnnotationResolver_AE 
 The annotation type to resolve overlaps
   PARAM_ANNOTATION_TYPE_TO_CONVERT   - 
Static variable in class edu.uchsc.ccp.uima.ae.util.converter. DocumentLevelAnnotationCreator_AE 
  
   PARAM_ANNOTATIONSET_IDS_TO_KEEP_LIST   - 
Static variable in class edu.uchsc.ccp.uima.ae.util.filter. AnnotationSetFilter_AE 
  
   PARAM_ANNOTATIONSET_VALIDATOR_FILE   - 
Static variable in class edu.uchsc.ccp.uima.ae.nlp.parser. StanfordParser_AE 
  
   PARAM_ANNOTATOR_ID   - 
Static variable in class edu.uchsc.ccp.uima.ae.util.converter. AnnotatorConverter_AE 
  
   PARAM_ANNOTATOR_ID   - 
Static variable in class edu.uchsc.ccp.uima.ae.util.converter. AnnotatorOverrider_AE 
  
   PARAM_ANNOTATOR_IDS_TO_CONVERT   - 
Static variable in class edu.uchsc.ccp.uima.ae.util.converter. AnnotatorConverter_AE 
  
   PARAM_ANNOTATOR_IDS_TO_KEEP_LIST   - 
Static variable in class edu.uchsc.ccp.uima.ae.util.filter. AnnotatorFilter_AE 
  
   PARAM_ANNOTATOR_VALIDATOR_FILE   - 
Static variable in class edu.uchsc.ccp.uima.ae.nlp.parser. StanfordParser_AE 
  
   PARAM_ANNOTATORS_TO_IGNORE   - 
Static variable in class edu.uchsc.ccp.uima.ae.util.converter. DocumentLevelAnnotationCreator_AE 
  
   PARAM_ATTACH_TYPED_DEPENDENCIES   - 
Static variable in class edu.uchsc.ccp.uima.ae.nlp.parser. StanfordParser_AE 
  
   PARAM_CLASS_MENTION_TYPES_TO_KEEP_LIST   - 
Static variable in class edu.uchsc.ccp.uima.ae.util.filter. ClassMentionTypeFilter_AE 
  
   PARAM_CLASS_MENTION_TYPES_TO_REMOVE_LIST   - 
Static variable in class edu.uchsc.ccp.uima.ae.util.filter. ClassMentionRemovalFilter_AE 
  
   PARAM_CONFIG_FILE   - 
Static variable in class edu.uchsc.ccp.uima.cc.summary.comparison. AnnotationComparator_CC 
  
   PARAM_CONSENSUS_THRESHOLD   - 
Static variable in class edu.uchsc.ccp.uima.ae.util.filter. AnnotationConsensusFilter_AE 
  
   PARAM_FIRST_NAME   - 
Static variable in class edu.uchsc.ccp.uima.ae.util.converter. AnnotatorConverter_AE 
  
   PARAM_FIRST_NAME   - 
Static variable in class edu.uchsc.ccp.uima.ae.util.converter. AnnotatorOverrider_AE 
  
   PARAM_GENIA_TAGGER_OUTPUT_FILE   - 
Variable in class edu.uchsc.ccp.uima.ae.nlp.tokenizer. GeniaTaggerOutputFileLoader_AE 
  
   PARAM_KEEP_LONGEST_COMMON_SUBSTRING   - 
Variable in class edu.uchsc.ccp.uima.ae.util.filter. OverlappingAnnotationResolver_AE 
  
   PARAM_KEXBASEDIRECTORY   - 
Static variable in class edu.uchsc.ccp.uima.ae.nlp.sentenceDetector. KeXSentenceDetector_AE 
  
   PARAM_LAST_NAME   - 
Static variable in class edu.uchsc.ccp.uima.ae.util.converter. AnnotatorConverter_AE 
  
   PARAM_LAST_NAME   - 
Static variable in class edu.uchsc.ccp.uima.ae.util.converter. AnnotatorOverrider_AE 
  
   PARAM_LINGPIPE_ENTITY_MODEL_FILE   - 
Static variable in class edu.uchsc.ccp.uima.ae.nlp.ei.gene. LingPipeEntityTagger_AE 
  
   PARAM_LINGPIPE_ENTITY_TYPE   - 
Static variable in class edu.uchsc.ccp.uima.ae.nlp.ei.gene. LingPipeEntityTagger_AE 
  
   PARAM_LINGPIPE_MODEL_FILE   - 
Static variable in class edu.uchsc.ccp.uima.ae.nlp.tokenizer. LingPipePOSTagger_AE 
  
   PARAM_MENTION_TYPES_TO_CONVERT   - 
Static variable in class edu.uchsc.ccp.uima.ae.util.converter. ClassMentionConverter_AE 
  
   PARAM_MODEL_FILE   - 
Static variable in class edu.uchsc.ccp.uima.ae.nlp.sentenceDetector. OpenNLPSentenceDetector_AE 
  
   PARAM_OUTPUT_INTO_MULTIPLE_FILES   - 
Variable in class edu.uchsc.ccp.uima.cc.summary. AnnotationPrinter 
  
   PARAM_OUTPUTFILENAME   - 
Variable in class edu.uchsc.ccp.uima.cc.summary. AnnotationPrinter 
 The path to the output file must be included in the descriptor under "OutputFileName"
   PARAM_OUTPUTTOFILE   - 
Variable in class edu.uchsc.ccp.uima.cc.summary. AnnotationPrinter 
 A boolean flag represented by "OutputToFile" in the descriptor file.
   PARAM_REGEX_FILE   - 
Static variable in class edu.uchsc.ccp.uima.ae.nlp.ei.mutation. MutationFinder_AE 
  
   PARAM_REQUIRED_SLOTS   - 
Static variable in class edu.uchsc.ccp.uima.ae.util.converter. DocumentLevelAnnotationCreator_AE 
  
   PARAM_SERIALIZED_GRAMMAR_FILE   - 
Static variable in class edu.uchsc.ccp.uima.ae.nlp.parser. StanfordParser_AE 
 The location of the serialized grammar file to be used by the Stanford Parser must be included in the descriptor file under
 "SerializedGrammarFile"
   PARAM_SETDESCRIPTION   - 
Static variable in class edu.uchsc.ccp.uima.ae.util.converter. AnnotationSetOverrider_AE 
  
   PARAM_SETID   - 
Static variable in class edu.uchsc.ccp.uima.ae.util.converter. AnnotationSetOverrider_AE 
  
   PARAM_SETNAME   - 
Static variable in class edu.uchsc.ccp.uima.ae.util.converter. AnnotationSetOverrider_AE 
  
   PARAM_SLOTS_TO_REMOVE_LIST   - 
Static variable in class edu.uchsc.ccp.uima.ae.util.filter. SlotRemovalFilter_AE 
  
   PARAM_TAGGER_MODEL_FILE   - 
Static variable in class edu.uchsc.ccp.uima.ae.nlp.ei.gene. PennBioTagger_AE 
  
   PARAM_TAGGER_TYPE   - 
Static variable in class edu.uchsc.ccp.uima.ae.nlp.ei.gene. PennBioTagger_AE 
  
   PARAM_TARGET_MENTION   - 
Static variable in class edu.uchsc.ccp.uima.ae.util.converter. ClassMentionConverter_AE 
  
   PARAM_TOKENIZER_MODEL_FILE   - 
Static variable in class edu.uchsc.ccp.uima.ae.nlp.ei.gene. PennBioTagger_AE 
  
   PARAM_TOKENIZER_MODEL_FILE   - 
Static variable in class edu.uchsc.ccp.uima.ae.nlp.tokenizer. PennBioTokenizer_AE 
 The location of the serialized grammar file to be used by the Stanford Parser must be included in the descriptor file under
 "SerializedGrammarFile"
   PARAM_USE_ALT_EVAL_FORMAT   - 
Variable in class edu.uchsc.ccp.uima.cc.summary. AnnotationPrinter 
  
   PARAM_USE_ANNOTATION_TO_FILE_FORMAT   - 
Variable in class edu.uchsc.ccp.uima.cc.summary. AnnotationPrinter 
  
   PARAM_USE_BIOCREATIVE_MODEL   - 
Static variable in class edu.uchsc.ccp.uima.ae.nlp.ei.gene. ABNER_AE 
  
   PARAM_USE_BIOCREATIVEFORMAT   - 
Variable in class edu.uchsc.ccp.uima.cc.summary. AnnotationPrinter 
  
   PARAM_USE_BIOCREATIVENORMFORMAT   - 
Variable in class edu.uchsc.ccp.uima.cc.summary. AnnotationPrinter 
  
   PARAM_USE_BRILLFORMAT   - 
Variable in class edu.uchsc.ccp.uima.cc.summary. AnnotationPrinter 
 A boolean flag represented by "UseTnTFormat" in the descriptor file.
   PARAM_USE_EMBEDDEDXMLFORMAT   - 
Variable in class edu.uchsc.ccp.uima.cc.summary. AnnotationPrinter 
  
   PARAM_USE_IDENTICAL_MENTION_COMPARATOR   - 
Static variable in class edu.uchsc.ccp.uima.cc.summary.comparison. AnnotationComparator_CC 
  
   PARAM_USE_IGNORE_SPAN_COMPARATOR   - 
Static variable in class edu.uchsc.ccp.uima.cc.summary.comparison. AnnotationComparator_CC 
  
   PARAM_USE_MUTATION_OUTPUT_FORMAT   - 
Variable in class edu.uchsc.ccp.uima.cc.summary. AnnotationPrinter 
  
   PARAM_USE_ONELINE_FORMAT   - 
Variable in class edu.uchsc.ccp.uima.cc.summary. AnnotationPrinter 
  
   PARAM_USE_OVERLAP_SPAN_COMPARATOR   - 
Static variable in class edu.uchsc.ccp.uima.cc.summary.comparison. AnnotationComparator_CC 
  
   PARAM_USE_SHARED_END_SPAN_COMPARATOR   - 
Static variable in class edu.uchsc.ccp.uima.cc.summary.comparison. AnnotationComparator_CC 
  
   PARAM_USE_SHARED_START_OR_END_SPAN_COMPARATOR   - 
Static variable in class edu.uchsc.ccp.uima.cc.summary.comparison. AnnotationComparator_CC 
  
   PARAM_USE_SHARED_START_SPAN_COMPARATOR   - 
Static variable in class edu.uchsc.ccp.uima.cc.summary.comparison. AnnotationComparator_CC 
  
   PARAM_USE_SIMPLE_COLLECTION_READER_FORMAT   - 
Variable in class edu.uchsc.ccp.uima.cc.summary. AnnotationPrinter 
  
   PARAM_USE_STRICT_SPAN_COMPARATOR   - 
Static variable in class edu.uchsc.ccp.uima.cc.summary.comparison. AnnotationComparator_CC 
  
   PARAM_USE_THING_WITH_ENTREZ_ID_FORMAT   - 
Variable in class edu.uchsc.ccp.uima.cc.summary. AnnotationPrinter 
  
   PARAM_USE_WORDFREAKFORMAT   - 
Variable in class edu.uchsc.ccp.uima.cc.summary. AnnotationPrinter 
 A boolean flag represented by "UseWordFreakFormat" in the descriptor file.
   parse(String)   - 
Method in class edu.uchsc.ccp.util.external.stanfordparser. StanfordParser_Util 
 Call this method to parse an inputted string of text.
   parseBio1Corpus(BufferedReader)   - 
Static method in class edu.uchsc.ccp.util.nlp.parser.corpus. Bio1CorpusParser 
 This method parses the corpus in one pass...
   parseBioIE_AnnotationFile(BufferedReader, String, String, String)   - 
Static method in class edu.uchsc.ccp.util.nlp.parser.corpus. BioIECorpusParser 
  
   parseBioIE_SourceFile(BufferedReader, int)   - 
Static method in class edu.uchsc.ccp.util.nlp.parser.corpus. BioIECorpusParser 
 returns a String Array of size 2 
 String[0] = title 
 String[1] = abstract 
 String[2] = documentID 
   parseEntitiesFromSparseUntokenizedXML(String, String, String, String, int, String, Annotator, AnnotationSet)   - 
Static method in class edu.uchsc.ccp.util.nlp.ei.xml. XMLEntityExtractor 
 Use when trying to get entity spans from xml that has not been tokenized (you therefore do not need the original text)
 NOTE: Due to the primitive nature of this code, be sure to remove all xml tags except for the one you are looking for.
   parseEntitiesFromSparseUntokenizedXML(String, String, String, String, int, String, Annotator, AnnotationSet, boolean)   - 
Static method in class edu.uchsc.ccp.util.nlp.ei.xml. XMLEntityExtractor 
  
   parseEntitiesFromSparseXML(String, String, String, String, String, Annotator)   - 
Static method in class edu.uchsc.ccp.util.nlp.ei.xml. XMLEntityExtractor 
  
   parseGeniaText(String)   - 
Static method in class edu.uchsc.ccp.util.external.lingpipe. LingPipe_Util 
 parse input text that is in the GENIA (XML-embedded entity) format
   parseIProLinkCorpus1(BufferedReader)   - 
Static method in class edu.uchsc.ccp.util.nlp.parser.corpus. IProLinkParser 
 corpus1.xml has tags: protein, acronym, and compound-protein
   parseIProLinkCorpus2(BufferedReader)   - 
Static method in class edu.uchsc.ccp.util.nlp.parser.corpus. IProLinkParser 
 corpus2.xml has tags: protein and long-form
   parsePartiallyTaggedText(String, String)   - 
Method in class edu.uchsc.ccp.util.external.stanfordparser. StanfordParser_Util 
 Not currently functional
   parseTexasCorpusDirectory(String)   - 
Static method in class edu.uchsc.ccp.util.nlp.parser.corpus. TexasCorpusParser 
 Parse the corpus and store all annotations and documents into the annotation database
   parseTexasCorpusFile(String, BufferedReader)   - 
Static method in class edu.uchsc.ccp.util.nlp.parser.corpus. TexasCorpusParser 
 This method parses the Texas corpus.
   parseTypedDependencyClsString(String)   - 
Static method in class edu.uchsc.ccp.util.external.stanfordparser. TypedDependencyCls_Util 
 parses a string taking the form of "gov:# dep:# rel:relation" and returns a TypedDependency
   PART_OF   - 
Static variable in interface edu.uchsc.ccp.util.nlp.mention. SlotMentionTypes 
  
   PDG_ACTION   - 
Static variable in interface edu.uchsc.ccp.util.nlp.mention. ClassMentionTypes 
  
   PennBioTagger_AE   - Class in  edu.uchsc.ccp.uima.ae.nlp.ei.gene      PennBioTagger_AE()   - 
Constructor for class edu.uchsc.ccp.uima.ae.nlp.ei.gene. PennBioTagger_AE 
  
   PennBioTaggerEISystem   - Class in  edu.uchsc.ccp.util.external.penn      PennBioTaggerEISystem(String, String, String)   - 
Constructor for class edu.uchsc.ccp.util.external.penn. PennBioTaggerEISystem 
 Initialize a new tagger.
   PennBioTokenizer_AE   - Class in  edu.uchsc.ccp.uima.ae.nlp.tokenizer  This class incorporates the StanfordParser into the UIMA analysis engine framework via the StanfordParserUtil.   PennBioTokenizer_AE()   - 
Constructor for class edu.uchsc.ccp.uima.ae.nlp.tokenizer. PennBioTokenizer_AE 
  
   PennBioTokenizer_Util   - Class in  edu.uchsc.ccp.util.external.penn      PennBioTokenizer_Util(String)   - 
Constructor for class edu.uchsc.ccp.util.external.penn. PennBioTokenizer_Util 
  
   PHENYLALANINE   - 
Static variable in interface edu.uchsc.ccp.util.nlp.mention. ClassMentionTypes 
  
   PHRASE   - 
Static variable in interface edu.uchsc.ccp.util.nlp.mention. ClassMentionTypes 
  
   PHRASE_LEVEL   - 
Static variable in interface edu.uchsc.ccp.util.nlp.mention. SlotMentionTypes 
  
   PHRASE_NUMBER   - 
Static variable in interface edu.uchsc.ccp.util.nlp.mention. SlotMentionTypes 
  
   PHRASE_TYPE   - 
Static variable in interface edu.uchsc.ccp.util.nlp.mention. SlotMentionTypes 
  
   POLYPEPTIDE_SEQUENCE   - 
Static variable in interface edu.uchsc.ccp.util.nlp.mention. ClassMentionTypes 
  
   POLYPEPTIDE_SUBSEQUENCE   - 
Static variable in interface edu.uchsc.ccp.util.nlp.mention. ClassMentionTypes 
  
   PorterStemmer_AE   - Class in  edu.uchsc.ccp.uima.ae.nlp.stemmer  This class incorporates the Porter stemming algorithm into a UIMA analysis engine framework.   PorterStemmer_AE()   - 
Constructor for class edu.uchsc.ccp.uima.ae.nlp.stemmer. PorterStemmer_AE 
  
   PorterStemmer_Util   - Class in  edu.uchsc.ccp.util.nlp.stemmer  Extends the original Porter Stemmer implementation by Martin Porter (See  PorterStemmer ) to accept  String  input.   PorterStemmer_Util()   - 
Constructor for class edu.uchsc.ccp.util.nlp.stemmer. PorterStemmer_Util 
  
   PRFResult   - Class in  edu.uchsc.ccp.util.nlp.stats  This class is a data structure for cataloging the results of an NLP experiment.   PRFResult(int, int, int)   - 
Constructor for class edu.uchsc.ccp.util.nlp.stats. PRFResult 
 Initialize a new PRFResult by inputting counts for true positives, false positives, and false negatives.
   PRFResult(int, int, int, String)   - 
Constructor for class edu.uchsc.ccp.util.nlp.stats. PRFResult 
 Initialize a new PRFResult by inputting counts for true positives, false positives, and false negatives.
   print(PrintStream)   - 
Method in class edu.uchsc.ccp.uima.cc.summary.comparison. AnnotationGroup 
  
   print(PrintStream)   - 
Method in class edu.uchsc.ccp.uima.cc.summary.comparison. ComparisonGroup 
  
   print(PrintStream, int)   - 
Method in class edu.uchsc.ccp.uima.util.opendmap. AnnotationMap 
 Print out the contents of this annotation map in a human-readable format.
   print(PrintStream)   - 
Method in class edu.uchsc.ccp.uima.util.opendmap. DMAPStanfordPhraseGraph 
 Print a human readable version of this phrase graph.
   printAnnotation(PrintStream)   - 
Method in class edu.uchsc.ccp.util.nlp.annotation. TextAnnotation 
 Output a representation of this TextAnnotation to the input PrintStream
   printAnnotationOnOneLine(PrintStream)   - 
Method in class edu.uchsc.ccp.util.nlp.annotation. TextAnnotation 
 simple output on one line..
   printAnnotations(PrintStream)   - 
Method in class edu.uchsc.ccp.util.nlp.stats. PRFResult 
 Output to the given  PrintStream  the annotations responsible for the TP, FP, and FN statistics.
   printCCPAnnotationSet(CCPAnnotationSet, PrintStream)   - 
Static method in class edu.uchsc.ccp.uima.util. UIMA_Util 
  
   printCCPAnnotator(CCPAnnotator, PrintStream)   - 
Static method in class edu.uchsc.ccp.uima.util. UIMA_Util 
  
   printCCPClassMention(CCPClassMention, PrintStream, int)   - 
Static method in class edu.uchsc.ccp.uima.util. UIMA_Util 
  
   printCCPComplexSlotMention(CCPComplexSlotMention, PrintStream, int)   - 
Static method in class edu.uchsc.ccp.uima.util. UIMA_Util 
  
   printCCPNonComplexSlotMention(CCPNonComplexSlotMention, PrintStream, int)   - 
Static method in class edu.uchsc.ccp.uima.util. UIMA_Util 
  
   printCCPTextAnnotation(CCPTextAnnotation, PrintStream)   - 
Static method in class edu.uchsc.ccp.uima.util. UIMA_Util 
  
   printDocument(GenericDocument, PrintStream)   - 
Method in interface edu.uchsc.ccp.util.nlp.annotation.printer. AnnotationPrinterUtil 
  
   printDocument(GenericDocument)   - 
Method in interface edu.uchsc.ccp.util.nlp.annotation.printer. AnnotationPrinterUtil 
  
   printDocument(GenericDocument)   - 
Method in class edu.uchsc.ccp.util.nlp.annotation.printer. AnnotationToFileOutput 
  
   printDocument(GenericDocument, PrintStream)   - 
Method in class edu.uchsc.ccp.util.nlp.annotation.printer. AnnotationToFileOutput 
  
   printDocument(GenericDocument)   - 
Method in class edu.uchsc.ccp.util.nlp.annotation.printer. DefaultPrintOutput 
  
   printDocument(GenericDocument, PrintStream)   - 
Method in class edu.uchsc.ccp.util.nlp.annotation.printer. DefaultPrintOutput 
  
   printDocument(GenericDocument)   - 
Method in class edu.uchsc.ccp.util.nlp.annotation.printer. SimpleCollectionReaderFormatOutput 
  
   printDocument(GenericDocument, PrintStream)   - 
Method in class edu.uchsc.ccp.util.nlp.annotation.printer. SimpleCollectionReaderFormatOutput 
  
   printDocument(PrintStream)   - 
Method in class edu.uchsc.ccp.util.nlp.document. GenericDocument 
  
   printDocumentCollection(List<GenericDocument>, PrintStream)   - 
Method in interface edu.uchsc.ccp.util.nlp.annotation.printer. AnnotationPrinterUtil 
  
   printDocumentCollection(List<GenericDocument>)   - 
Method in interface edu.uchsc.ccp.util.nlp.annotation.printer. AnnotationPrinterUtil 
  
   printDocumentCollection(List<GenericDocument>, PrintStream)   - 
Method in class edu.uchsc.ccp.util.nlp.annotation.printer. AnnotationToFileOutput 
  
   printDocumentCollection(List<GenericDocument>)   - 
Method in class edu.uchsc.ccp.util.nlp.annotation.printer. AnnotationToFileOutput 
  
   printDocumentCollection(List<GenericDocument>)   - 
Method in class edu.uchsc.ccp.util.nlp.annotation.printer. DefaultPrintOutput 
  
   printDocumentCollection(List<GenericDocument>, PrintStream)   - 
Method in class edu.uchsc.ccp.util.nlp.annotation.printer. DefaultPrintOutput 
  
   printDocumentCollection(List<GenericDocument>)   - 
Method in class edu.uchsc.ccp.util.nlp.annotation.printer. SimpleCollectionReaderFormatOutput 
  
   printDocumentCollection(List<GenericDocument>, PrintStream)   - 
Method in class edu.uchsc.ccp.util.nlp.annotation.printer. SimpleCollectionReaderFormatOutput 
  
   printMention(ClassMention, PrintStream, int)   - 
Static method in class edu.uchsc.ccp.util.nlp.mention. ClassMention 
  
   printMention(ComplexSlotMention, PrintStream, int)   - 
Static method in class edu.uchsc.ccp.util.nlp.mention. ComplexSlotMention 
  
   printMention(SlotMention, PrintStream, int)   - 
Static method in class edu.uchsc.ccp.util.nlp.mention. SlotMention 
 Output a description of this slot mention
   printStats(PrintStream)   - 
Method in class edu.uchsc.ccp.util.nlp.stats. PRFResult 
 Output to the given  PrintStream  the statistics recorded by this PRFResult
   printStorageLine(PrintStream)   - 
Method in class edu.uchsc.ccp.util.nlp.annotation. AnnotationSet 
  
   printStorageLine(PrintStream)   - 
Method in class edu.uchsc.ccp.util.nlp.annotation. Annotator 
  
   process(JCas, ResultSpecification)   - 
Method in class edu.uchsc.ccp.uima.ae.nlp.anaphora. ProteinAnaphoraResolver 
  
   process(JCas, ResultSpecification)   - 
Method in class edu.uchsc.ccp.uima.ae.nlp.ei.gene. AbGene_AE 
  
   process(JCas, ResultSpecification)   - 
Method in class edu.uchsc.ccp.uima.ae.nlp.ei.gene. ABNER_AE 
 This method employs the ABNER entity identification system to search the document text for Gene, RNA, DNA, Cell Line, and Cell Type
 mentions.
   process(JCas, ResultSpecification)   - 
Method in class edu.uchsc.ccp.uima.ae.nlp.ei.gene. KeX_AE 
  
   process(JCas, ResultSpecification)   - 
Method in class edu.uchsc.ccp.uima.ae.nlp.ei.gene. LingPipeEntityTagger_AE 
  
   process(JCas, ResultSpecification)   - 
Method in class edu.uchsc.ccp.uima.ae.nlp.ei.gene. PennBioTagger_AE 
  
   process(JCas, ResultSpecification)   - 
Method in class edu.uchsc.ccp.uima.ae.nlp.ei.mutation. MutationFinder_AE 
  
   process(Parser, JCas, ResultSpecification)   - 
Method in class edu.uchsc.ccp.uima.ae.nlp.opendmap. CCPOpenDMAP_AE 
 Process the JCas using the annotation map to help extract input tokens.
   process(JCas, ResultSpecification)   - 
Method in class edu.uchsc.ccp.uima.ae.nlp.opendmap. OpenDmapAnnotator 
 When the UIMA pipeline signals it is time to run this
 annotator, call user code to get tokens from the jcas,
 call the parser, and then write annotations back to the
 jcas.
   process(Parser, JCas, ResultSpecification)   - 
Method in class edu.uchsc.ccp.uima.ae.nlp.opendmap. OpenDmapAnnotator 
 Process the JCas as part of a UIMA pipeline.
   process(JCas, ResultSpecification)   - 
Method in class edu.uchsc.ccp.uima.ae.nlp.parser. StanfordParser_AE 
 This method processes the document text, and incorporates into the CAS TokenAnnotations, PhraseAnnotations, and ClauseAnnotations based on the
 output of the Stanford Parser
   process(JCas, ResultSpecification)   - 
Method in class edu.uchsc.ccp.uima.ae.nlp.sentenceDetector. KeXSentenceDetector_AE 
  
   process(JCas, ResultSpecification)   - 
Method in class edu.uchsc.ccp.uima.ae.nlp.sentenceDetector. LingPipeSentenceDetector_AE 
  
   process(JCas, ResultSpecification)   - 
Method in class edu.uchsc.ccp.uima.ae.nlp.sentenceDetector. OpenNLPSentenceDetector_AE 
  
   process(JCas, ResultSpecification)   - 
Method in class edu.uchsc.ccp.uima.ae.nlp.stemmer. PorterStemmer_AE 
 For each TokenAnnotation, this method will determine the stem of the token, and fill in the Stem field for the TokenAnnotation.
   process(JCas, ResultSpecification)   - 
Method in class edu.uchsc.ccp.uima.ae.nlp.tokenizer. GeniaTaggerOutputFileLoader_AE 
  
   process(JCas, ResultSpecification)   - 
Method in class edu.uchsc.ccp.uima.ae.nlp.tokenizer. LingPipePOSTagger_AE 
 This method processes the document text, and incorporates into the CAS TokenAnnotations based on the output of the LingPipe POS Tagger
   process(JCas, ResultSpecification)   - 
Method in class edu.uchsc.ccp.uima.ae.nlp.tokenizer. PennBioTokenizer_AE 
 This method processes the document text, and incorporates into the CAS TokenAnnotations, PhraseAnnotations, and ClauseAnnotations
 based on the output of the Stanford Parser
   process(JCas, ResultSpecification)   - 
Method in class edu.uchsc.ccp.uima.ae.util. AnnotationFromFileLoader_AE 
  
   process(JCas, ResultSpecification)   - 
Method in class edu.uchsc.ccp.uima.ae.util.converter. AnnotationSetOverrider_AE 
 cycle through all annotations and set the annotation set
   process(JCas, ResultSpecification)   - 
Method in class edu.uchsc.ccp.uima.ae.util.converter. AnnotatorConverter_AE 
 cycle through all annotations and set the annotation set
   process(JCas, ResultSpecification)   - 
Method in class edu.uchsc.ccp.uima.ae.util.converter. AnnotatorOverrider_AE 
 cycle through all annotations and set the annotation set
   process(JCas, ResultSpecification)   - 
Method in class edu.uchsc.ccp.uima.ae.util.converter. ClassMentionConverter_AE 
 cycle through all annotations and change the mention if appropriate
   process(JCas, ResultSpecification)   - 
Method in class edu.uchsc.ccp.uima.ae.util.converter. DocumentLevelAnnotationCreator_AE 
 cycle through all annotations and convert the appropriate ones to document-level annotations
   process(JCas, ResultSpecification)   - 
Method in class edu.uchsc.ccp.uima.ae.util.filter. AnnotationConsensusFilter_AE 
  
   process(JCas, ResultSpecification)   - 
Method in class edu.uchsc.ccp.uima.ae.util.filter. AnnotationSetFilter_AE 
 cycle through all annotations and set the annotation set
   process(JCas, ResultSpecification)   - 
Method in class edu.uchsc.ccp.uima.ae.util.filter. AnnotatorFilter_AE 
 cycle through all annotations and set the annotation set
   process(JCas, ResultSpecification)   - 
Method in class edu.uchsc.ccp.uima.ae.util.filter. ClassMentionRemovalFilter_AE 
 cycle through all annotations and set the annotation set
   process(JCas, ResultSpecification)   - 
Method in class edu.uchsc.ccp.uima.ae.util.filter. ClassMentionTypeFilter_AE 
 cycle through all annotations and set the annotation set
   process(JCas, ResultSpecification)   - 
Method in class edu.uchsc.ccp.uima.ae.util.filter. OverlappingAnnotationResolver_AE 
 Default behavior is to keep the longest of any overlapping annotation
   process(JCas, ResultSpecification)   - 
Method in class edu.uchsc.ccp.uima.ae.util.filter. SlotRemovalFilter_AE 
 cycle through all annotations and remove any slots that have been inputted by the user
   processAnnotations(List<TextAnnotation>)   - 
Method in class edu.uchsc.ccp.util.nlp.annotation.filter. OverlappingAnnotationResolver 
  
   processBioIECorpusDirectory(String)   - 
Static method in class edu.uchsc.ccp.util.nlp.parser.corpus. BioIECorpusParser 
  
   processCas(CAS)   - 
Method in class edu.uchsc.ccp.uima.cc.summary. AnnotationPrinter 
 Prints out the CAS annotations in the format specified by the descriptor file.
   processCas(CAS)   - 
Method in class edu.uchsc.ccp.uima.cc.summary.comparison. AnnotationComparator_CC 
  
   processCas(CAS)   - 
Method in class edu.uchsc.ccp.uima.cc.summary.opendmap. TestPrinter 
 The standard UIMA process method.
   PROCESSED_TEXT_SLOT   - 
Static variable in interface edu.uchsc.ccp.util.nlp.mention. SlotMentionTypes 
 Document-level Slot Mentions
   PROLINE   - 
Static variable in interface edu.uchsc.ccp.util.nlp.mention. ClassMentionTypes 
  
   PROTEIN   - 
Static variable in interface edu.uchsc.ccp.util.nlp.mention. ClassMentionTypes 
 Semantic Class Mentions
   PROTEIN_ACRONYM   - 
Static variable in interface edu.uchsc.ccp.util.nlp.mention. ClassMentionTypes 
  
   PROTEIN_COMPLEX   - 
Static variable in interface edu.uchsc.ccp.util.nlp.mention. ClassMentionTypes 
  
   PROTEIN_COMPOUND   - 
Static variable in interface edu.uchsc.ccp.util.nlp.mention. ClassMentionTypes 
  
   PROTEIN_ENTREZGENEID   - 
Static variable in interface edu.uchsc.ccp.util.nlp.mention. SlotMentionTypes 
 Semantic Slot Mentions
   PROTEIN_LONG_FORM   - 
Static variable in interface edu.uchsc.ccp.util.nlp.mention. ClassMentionTypes 
  
   ProteinAnaphoraResolver   - Class in  edu.uchsc.ccp.uima.ae.nlp.anaphora  This class is based on a simple anaphora algorithm that Z came up with.   ProteinAnaphoraResolver()   - 
Constructor for class edu.uchsc.ccp.uima.ae.nlp.anaphora. ProteinAnaphoraResolver 
  
   putTextAnnotationsIntoJCas(JCas, List<TextAnnotation>)   - 
Method in class edu.uchsc.ccp.uima.util. UIMA_Util 
  
 
 
    
 R  
 
   recordFunction(String, String, Reference)   - 
Method in class edu.uchsc.ccp.uima.util.opendmap. DMAPStanfordPhraseGraph 
 Record a property function constraint for later evaluation.
   ReferenceProcessor   - Class in  edu.uchsc.ccp.uima.util.opendmap  Get the the desired references that DMAP has recognized.   ReferenceProcessor()   - 
Constructor for class edu.uchsc.ccp.uima.util.opendmap. ReferenceProcessor 
  
   remove()   - 
Method in class edu.uchsc.ccp.util.external.geniatagger. GeniaTaggerOutputFileParser 
  
   remove()   - 
Method in class edu.uchsc.ccp.util.nlp.parser.corpus. CorpusParser 
  
   remove()   - 
Method in class edu.uchsc.ccp.util.nlp.parser.corpus. GeniaCorpusParser 
 The remove operation is not currently supported.
   removeLeadingAndTrailingBlankLines(Annotation)   - 
Static method in class edu.uchsc.ccp.uima.util. UIMA_Annotation_Util 
  
   removeStopWords(String)   - 
Static method in class edu.uchsc.ccp.util.nlp. GenericTextUtils 
 Removes stop words from the input text string.
   replaceXMLspecialCharacters(String)   - 
Static method in class edu.uchsc.ccp.util.nlp. GenericTextUtils 
 Replaces XML special characters with their ASCII equivalents & --> &, etc.
   reset()   - 
Method in class edu.uchsc.ccp.util.external.kex. KeXEISystem 
 Deletes the previous output file if it exists.
   reset()   - 
Static method in class edu.uchsc.ccp.util.nlp.parser.corpus. BioIECorpusParser 
  
   RNA   - 
Static variable in interface edu.uchsc.ccp.util.nlp.mention. ClassMentionTypes 
  
   run(String, boolean)   - 
Static method in class edu.uchsc.ccp.util. RunExecutable 
 Run a command line executable.
   RunExecutable   - Class in  edu.uchsc.ccp.util  This class is a simple utility for calling command line executables.   RunExecutable()   - 
Constructor for class edu.uchsc.ccp.util. RunExecutable 
  
   runPOSTagger(String, String)   - 
Static method in class edu.uchsc.ccp.util.external.lingpipe. LingPipe_Util 
 Much of this code was taken from: http://alias-i.com/lingpipe/demos/tutorial/posTags/src/RunMedPost.java NOTE:
 initializeTaggingSystem() must be called before this method.
 
 
    
 S  
 
   segmentIntoSentences(String, String)   - 
Method in class edu.uchsc.ccp.util.external.opennlp. OpenNLPSentenceSegmenter 
  
   SENTENCE   - 
Static variable in interface edu.uchsc.ccp.util.nlp.mention. ClassMentionTypes 
  
   SERINE   - 
Static variable in interface edu.uchsc.ccp.util.nlp.mention. ClassMentionTypes 
  
   setAbstractText(String)   - 
Method in class edu.uchsc.ccp.util.nlp.document. GenericDocument 
  Deprecated.  
   setAffiliation(String)   - 
Method in class edu.uchsc.ccp.uima.annotation. CCPAnnotator 
 setter for affiliation - sets The affiliation of the annotator.
   setAffiliation(int, String)   - 
Method in class edu.uchsc.ccp.uima.annotation. CCPAnnotator_Type 
  
   setAffiliation(String)   - 
Method in class edu.uchsc.ccp.util.nlp.annotation. Annotator 
  
   setAnnotationGroupList(List<AnnotationGroupID>)   - 
Method in class edu.uchsc.ccp.uima.cc.summary.comparison. ComparisonGroup 
  
   setAnnotationID(int)   - 
Method in class edu.uchsc.ccp.uima.annotation. CCPTextAnnotation 
 setter for annotationID - sets The annotation ID provides a means for identifying a particular annotation.
   setAnnotationID(int, int)   - 
Method in class edu.uchsc.ccp.uima.annotation. CCPTextAnnotation_Type 
  
   setAnnotationID(int)   - 
Method in class edu.uchsc.ccp.util.nlp.annotation. TextAnnotation 
 Set the annotation ID
   setAnnotations(List<TextAnnotation>)   - 
Method in class edu.uchsc.ccp.util.nlp.document. GenericDocument 
  
   setAnnotationSetDescription(String)   - 
Method in class edu.uchsc.ccp.uima.annotation. CCPAnnotationSet 
 setter for annotationSetDescription - sets A textual description of an annotation set.
   setAnnotationSetDescription(int, String)   - 
Method in class edu.uchsc.ccp.uima.annotation. CCPAnnotationSet_Type 
  
   setAnnotationSetDescription(String)   - 
Method in class edu.uchsc.ccp.util.nlp.annotation. AnnotationSet 
  
   setAnnotationSetID(int)   - 
Method in class edu.uchsc.ccp.uima.annotation. CCPAnnotationSet 
 setter for annotationSetID - sets An integer uniquely identifying a particular annotation set.
   setAnnotationSetID(int, int)   - 
Method in class edu.uchsc.ccp.uima.annotation. CCPAnnotationSet_Type 
  
   setAnnotationSetID(Integer)   - 
Method in class edu.uchsc.ccp.uima.cc.summary.comparison. AnnotationGroup 
  
   setAnnotationSetID(Integer)   - 
Method in class edu.uchsc.ccp.util.nlp.annotation. AnnotationSet 
  
   setAnnotationSetName(String)   - 
Method in class edu.uchsc.ccp.uima.annotation. CCPAnnotationSet 
 setter for annotationSetName - sets The name of the annotation set.
   setAnnotationSetName(int, String)   - 
Method in class edu.uchsc.ccp.uima.annotation. CCPAnnotationSet_Type 
  
   setAnnotationSetName(String)   - 
Method in class edu.uchsc.ccp.util.nlp.annotation. AnnotationSet 
  
   setAnnotationSets(FSArray)   - 
Method in class edu.uchsc.ccp.uima.annotation. CCPTextAnnotation 
 setter for annotationSets - sets Annotation Sets provide an arbitrary means of categorizing and clustering annotations into groups.
   setAnnotationSets(int, CCPAnnotationSet)   - 
Method in class edu.uchsc.ccp.uima.annotation. CCPTextAnnotation 
 indexed setter for annotationSets - sets an indexed value - Annotation Sets provide an arbitrary means of categorizing and clustering annotations into groups.
   setAnnotationSets(int, int)   - 
Method in class edu.uchsc.ccp.uima.annotation. CCPTextAnnotation_Type 
  
   setAnnotationSets(int, int, int)   - 
Method in class edu.uchsc.ccp.uima.annotation. CCPTextAnnotation_Type 
  
   setAnnotationSets(Set<AnnotationSet>)   - 
Method in class edu.uchsc.ccp.util.nlp.annotation. TextAnnotation 
 Set the AnnotationSet membership for this TextAnnotation
   setAnnotationSpanEnd(int)   - 
Method in class edu.uchsc.ccp.util.nlp.annotation. TextAnnotation 
 Set the end index for the last annotation span
   setAnnotationSpanStart(int)   - 
Method in class edu.uchsc.ccp.util.nlp.annotation. TextAnnotation 
 Sets the start index for the first annotation span
   setAnnotationTypeList(List<String>)   - 
Method in class edu.uchsc.ccp.uima.cc.summary.comparison. AnnotationGroup 
  
   setAnnotator(CCPAnnotator)   - 
Method in class edu.uchsc.ccp.uima.annotation. CCPTextAnnotation 
 setter for annotator - sets The annotator was responsible for generating this annotation.
   setAnnotator(int, int)   - 
Method in class edu.uchsc.ccp.uima.annotation. CCPTextAnnotation_Type 
  
   setAnnotator(Annotator)   - 
Method in class edu.uchsc.ccp.util.nlp.annotation. TextAnnotation 
 Get the annotator
   setAnnotatorID(int)   - 
Method in class edu.uchsc.ccp.uima.annotation. CCPAnnotator 
 setter for annotatorID - sets This Integer should be a unique ID for a particular annotator.
   setAnnotatorID(int, int)   - 
Method in class edu.uchsc.ccp.uima.annotation. CCPAnnotator_Type 
  
   setAnnotatorID(Integer)   - 
Method in class edu.uchsc.ccp.uima.cc.summary.comparison. AnnotationGroup 
  
   setAnnotatorID(Integer)   - 
Method in class edu.uchsc.ccp.util.nlp.annotation. Annotator 
  
   setAnnotatorID(int)   - 
Method in class edu.uchsc.ccp.util.nlp.annotation. TextAnnotation 
  Deprecated.  
   setCCPPClassMentionForCCPTextAnnotation(CCPTextAnnotation, CCPClassMention)   - 
Static method in class edu.uchsc.ccp.uima.util. UIMA_Util 
 sets the class mention for the CCPTextAnnotation, and also links the CCPTextAnnotation with the CCPClassMention
   setCcpTextAnnotations(FSArray)   - 
Method in class edu.uchsc.ccp.uima.mention. CCPClassMention 
 setter for ccpTextAnnotations - sets Just as CCPTextAnnotations are linked to a CCPClassMention, it is sometimes useful to be able to follow a CCPClassMention back to its corresponding CCPTextAnnotations, therefore, this FSArray contains links to the CCPTextAnotation(s) for this class.
   setCcpTextAnnotations(int, CCPTextAnnotation)   - 
Method in class edu.uchsc.ccp.uima.mention. CCPClassMention 
 indexed setter for ccpTextAnnotations - sets an indexed value - Just as CCPTextAnnotations are linked to a CCPClassMention, it is sometimes useful to be able to follow a CCPClassMention back to its corresponding CCPTextAnnotations, therefore, this FSArray contains links to the CCPTextAnotation(s) for this class.
   setCcpTextAnnotations(int, int)   - 
Method in class edu.uchsc.ccp.uima.mention. CCPClassMention_Type 
  
   setCcpTextAnnotations(int, int, int)   - 
Method in class edu.uchsc.ccp.uima.mention. CCPClassMention_Type 
  
   setCCPTextAnnotationSpan(CCPTextAnnotation, int, int)   - 
Static method in class edu.uchsc.ccp.uima.util. UIMA_Util 
 this method resets the span for an annotation..
   setClassificationType(String)   - 
Method in class edu.uchsc.ccp.uima.annotation. CCPDocumentInformation 
 setter for classificationType - sets This String provides a means for classifying a particular document.
   setClassificationType(int, String)   - 
Method in class edu.uchsc.ccp.uima.annotation. CCPDocumentInformation_Type 
  
   setClassMention(CCPClassMention)   - 
Method in class edu.uchsc.ccp.uima.annotation. CCPTextAnnotation 
 setter for classMention - sets The CCP ClassMention indicates the type (or class) for this annotation.
   setClassMention(int, int)   - 
Method in class edu.uchsc.ccp.uima.annotation. CCPTextAnnotation_Type 
  
   setClassMention(ClassMention)   - 
Method in class edu.uchsc.ccp.util.nlp.annotation. TextAnnotation 
 When setting the classmention, the a reference to the text annotation is automatically created inside the classMention object.
   setClassMentions(FSArray)   - 
Method in class edu.uchsc.ccp.uima.mention. CCPComplexSlotMention 
 setter for classMentions - sets The class mentions which are the slot fillers for this complex slot.
   setClassMentions(int, CCPClassMention)   - 
Method in class edu.uchsc.ccp.uima.mention. CCPComplexSlotMention 
 indexed setter for classMentions - sets an indexed value - The class mentions which are the slot fillers for this complex slot.
   setClassMentions(int, int)   - 
Method in class edu.uchsc.ccp.uima.mention. CCPComplexSlotMention_Type 
  
   setClassMentions(int, int, int)   - 
Method in class edu.uchsc.ccp.uima.mention. CCPComplexSlotMention_Type 
  
   setClassMentions(List<ClassMention>)   - 
Method in class edu.uchsc.ccp.util.nlp.mention. ComplexSlotMention 
 Set the class mentions that fill this complex slot
   setClauseType(String)   - 
Method in class edu.uchsc.ccp.uima.annotation.syntactic. CCPClauseAnnotation 
 setter for clauseType - sets The clause type symbol is stored here.
   setClauseType(int, String)   - 
Method in class edu.uchsc.ccp.uima.annotation.syntactic. CCPClauseAnnotation_Type 
  
   setComplexSlotMentions(List<ComplexSlotMention>)   - 
Method in class edu.uchsc.ccp.util.nlp.mention. ClassMention 
  
   setCoveredText(String)   - 
Method in class edu.uchsc.ccp.util.nlp.annotation. TextAnnotation 
 Set the text that is "covered" by this annotation
   setDependentTokenNum(int)   - 
Method in class edu.uchsc.ccp.uima.annotation.syntactic. CCPTypedDependency 
 setter for dependentTokenNum - sets
   setDependentTokenNum(int, int)   - 
Method in class edu.uchsc.ccp.uima.annotation.syntactic. CCPTypedDependency_Type 
  
   setDependentTokenNum(int)   - 
Method in class edu.uchsc.ccp.util.external.stanfordparser. TypedDependencyCls 
  
   setDescription(String)   - 
Method in class edu.uchsc.ccp.uima.cc.summary.comparison. ComparisonGroup 
  
   setDocumentCollectionID(int)   - 
Method in class edu.uchsc.ccp.uima.annotation. CCPDocumentInformation 
 setter for documentCollectionID - sets The document collection ID is an Integer that uniquely identifies a particular document collection.
   setDocumentCollectionID(int, int)   - 
Method in class edu.uchsc.ccp.uima.annotation. CCPDocumentInformation_Type 
  
   setDocumentCollectionID(int)   - 
Method in class edu.uchsc.ccp.util.nlp.annotation. TextAnnotation 
 Set the document collection ID
   setDocumentCollectionID(int)   - 
Method in class edu.uchsc.ccp.util.nlp.document. GenericDocument 
  
   setDocumentID(String)   - 
Method in class edu.uchsc.ccp.uima.annotation. CCPDocumentInformation 
 setter for documentID - sets The document ID is a String representing a unique identifier for a particular document within a particular document collection.
   setDocumentID(int, String)   - 
Method in class edu.uchsc.ccp.uima.annotation. CCPDocumentInformation_Type 
  
   setDocumentID(String)   - 
Method in class edu.uchsc.ccp.util.nlp.annotation. TextAnnotation 
 Set the document ID
   setDocumentID(String)   - 
Method in class edu.uchsc.ccp.util.nlp.document. GenericDocument 
  
   setDocumentSectionID(int)   - 
Method in class edu.uchsc.ccp.uima.annotation. CCPTextAnnotation 
 setter for documentSectionID - sets The document section ID is optionally used to log what section of a document this annotation is from.
   setDocumentSectionID(int, int)   - 
Method in class edu.uchsc.ccp.uima.annotation. CCPTextAnnotation_Type 
  
   setDocumentSectionID(int)   - 
Method in class edu.uchsc.ccp.util.nlp.annotation. TextAnnotation 
 Set the document section ID
   setDocumentSectionID(int)   - 
Method in class edu.uchsc.ccp.util.nlp.document. DocumentSection 
  
   setDocumentSections(List<DocumentSection>)   - 
Method in class edu.uchsc.ccp.util.nlp.document. GenericDocument 
  
   setDocumentSize(int)   - 
Method in class edu.uchsc.ccp.uima.annotation. CCPDocumentInformation 
 setter for documentSize - sets The size of a document is logged as the number of characters it contains.
   setDocumentSize(int, int)   - 
Method in class edu.uchsc.ccp.uima.annotation. CCPDocumentInformation_Type 
  
   setDocumentText(String)   - 
Method in class edu.uchsc.ccp.util.nlp.document. GenericDocument 
  
   setFalseNegativeCount(int)   - 
Method in class edu.uchsc.ccp.util.nlp.stats. PRFResult 
  
   setFalsePositiveCount(int)   - 
Method in class edu.uchsc.ccp.util.nlp.stats. PRFResult 
  
   setFirstName(String)   - 
Method in class edu.uchsc.ccp.uima.annotation. CCPAnnotator 
 setter for firstName - sets The first name of the annotator.
   setFirstName(int, String)   - 
Method in class edu.uchsc.ccp.uima.annotation. CCPAnnotator_Type 
  
   setFirstName(String)   - 
Method in class edu.uchsc.ccp.util.nlp.annotation. Annotator 
  
   setFirstSlotValue(Object)   - 
Method in class edu.uchsc.ccp.util.nlp.mention. SlotMention 
 Set the first slot value.
   setFNAnnotations(List<TextAnnotation>)   - 
Method in class edu.uchsc.ccp.util.nlp.stats. PRFResult 
 Set the false negative  TextAnnotation  list
   setFPAnnotations(List<TextAnnotation>)   - 
Method in class edu.uchsc.ccp.util.nlp.stats. PRFResult 
 Set the false positive  TextAnnotation  list
   setGoldStandard(boolean)   - 
Method in class edu.uchsc.ccp.uima.cc.summary.comparison. ComparisonGroup 
  
   setGovernerTokenNum(int)   - 
Method in class edu.uchsc.ccp.util.external.stanfordparser. TypedDependencyCls 
  
   setGovernorTokenNum(int)   - 
Method in class edu.uchsc.ccp.uima.annotation.syntactic. CCPTypedDependency 
 setter for governorTokenNum - sets
   setGovernorTokenNum(int, int)   - 
Method in class edu.uchsc.ccp.uima.annotation.syntactic. CCPTypedDependency_Type 
  
   setGroupID(AnnotationGroupID)   - 
Method in class edu.uchsc.ccp.uima.cc.summary.comparison. AnnotationGroup 
  
   setId(String)   - 
Method in class edu.uchsc.ccp.uima.cc.summary.comparison. AnnotationGroupID 
  
   setInputMentionReferenceType(String, String)   - 
Method in class edu.uchsc.ccp.uima.util.opendmap. AnnotationMap 
 Set the type of DMAP Protege Frame to use for the input token corresponding
 to this type of CAS annotation mention.
   setInputMentionSlotReferenceType(String, String, String)   - 
Method in class edu.uchsc.ccp.uima.util.opendmap. AnnotationMap 
 Set whether or not a CAS annotation mention slot should be included in
 DMAP input token references.
   setKey(String)   - 
Method in class edu.uchsc.ccp.util.nlp. KeyValuePair 
 Sets the key
   setLastName(String)   - 
Method in class edu.uchsc.ccp.uima.annotation. CCPAnnotator 
 setter for lastName - sets The last name of the annotator.
   setLastName(int, String)   - 
Method in class edu.uchsc.ccp.uima.annotation. CCPAnnotator_Type 
  
   setLastName(String)   - 
Method in class edu.uchsc.ccp.util.nlp.annotation. Annotator 
  
   setLemma(String)   - 
Method in class edu.uchsc.ccp.uima.annotation.syntactic. CCPTokenAnnotation 
 setter for lemma - sets The token lemma.
   setLemma(int, String)   - 
Method in class edu.uchsc.ccp.uima.annotation.syntactic. CCPTokenAnnotation_Type 
  
   setMentionName(String)   - 
Method in class edu.uchsc.ccp.uima.mention. CCPMention 
 setter for mentionName - sets The name of this mention.
   setMentionName(int, String)   - 
Method in class edu.uchsc.ccp.uima.mention. CCPMention_Type 
  
   setMentionName(String)   - 
Method in class edu.uchsc.ccp.util.nlp.mention. Mention 
  Deprecated.  
   setNumberOfSpans(int)   - 
Method in class edu.uchsc.ccp.uima.annotation. CCPTextAnnotation 
 setter for numberOfSpans - sets The number of spans comprising this annotation.
   setNumberOfSpans(int, int)   - 
Method in class edu.uchsc.ccp.uima.annotation. CCPTextAnnotation_Type 
  
   setOutputReferenceMentionType(String, String)   - 
Method in class edu.uchsc.ccp.uima.util.opendmap. AnnotationMap 
 Set the type of CAS annotation mention that should be created to
 represent this DMAP reference type.
   setOutputReferenceRootSlot(String, String)   - 
Method in class edu.uchsc.ccp.uima.util.opendmap. AnnotationMap 
 Set the name of the slot holding the root reference for references of
 this DMAP type.
   setOutputReferenceSlotExcluded(String, String, boolean)   - 
Method in class edu.uchsc.ccp.uima.util.opendmap. AnnotationMap 
 Set whether an output reference slot should be included in the CAS
 annotation mention being written back to the CAS.
   setPartOfSpeech(String)   - 
Method in class edu.uchsc.ccp.uima.annotation.syntactic. CCPTokenAnnotation 
 setter for partOfSpeech - sets The part of speech symbol is stored here.
   setPartOfSpeech(int, String)   - 
Method in class edu.uchsc.ccp.uima.annotation.syntactic. CCPTokenAnnotation_Type 
  
   setPhraseType(String)   - 
Method in class edu.uchsc.ccp.uima.annotation.syntactic. CCPPhraseAnnotation 
 setter for phraseType - sets The phrase type symbol (e.g.
   setPhraseType(int, String)   - 
Method in class edu.uchsc.ccp.uima.annotation.syntactic. CCPPhraseAnnotation_Type 
  
   setProtegeProjectFilename(String)   - 
Method in class edu.uchsc.ccp.uima.ae.nlp.opendmap. OpenDmapAnnotator 
 Set the Protege Project file to be loaded into the
 OpenDMAP parser when this annotator is initialized.
   setRelation(String)   - 
Method in class edu.uchsc.ccp.uima.annotation.syntactic. CCPTypedDependency 
 setter for relation - sets
   setRelation(int, String)   - 
Method in class edu.uchsc.ccp.uima.annotation.syntactic. CCPTypedDependency_Type 
  
   setRelation(String)   - 
Method in class edu.uchsc.ccp.util.external.stanfordparser. TypedDependencyCls 
  
   setSecondaryDocumentIDs(StringArray)   - 
Method in class edu.uchsc.ccp.uima.annotation. CCPDocumentInformation 
 setter for secondaryDocumentIDs - sets This StringArray is used for secondary document ID storage.
   setSecondaryDocumentIDs(int, String)   - 
Method in class edu.uchsc.ccp.uima.annotation. CCPDocumentInformation 
 indexed setter for secondaryDocumentIDs - sets an indexed value - This StringArray is used for secondary document ID storage.
   setSecondaryDocumentIDs(int, int)   - 
Method in class edu.uchsc.ccp.uima.annotation. CCPDocumentInformation_Type 
  
   setSecondaryDocumentIDs(int, int, String)   - 
Method in class edu.uchsc.ccp.uima.annotation. CCPDocumentInformation_Type 
  
   setSecondaryDocumentIDs(List<String>)   - 
Method in class edu.uchsc.ccp.util.nlp.document. GenericDocument 
  Deprecated.  
   setSectionEndIndex(int)   - 
Method in class edu.uchsc.ccp.util.nlp.document. DocumentSection 
  
   setSectionID(int)   - 
Method in class edu.uchsc.ccp.uima.annotation. CCPDocumentSection 
 setter for sectionID - sets The section ID for this document section, e.g.
   setSectionID(int, int)   - 
Method in class edu.uchsc.ccp.uima.annotation. CCPDocumentSection_Type 
  
   setSectionLength(int)   - 
Method in class edu.uchsc.ccp.uima.annotation. CCPDocumentSection 
 setter for sectionLength - sets The length of this document section (in characters).
   setSectionLength(int, int)   - 
Method in class edu.uchsc.ccp.uima.annotation. CCPDocumentSection_Type 
  
   setSectionName(String)   - 
Method in class edu.uchsc.ccp.uima.annotation. CCPDocumentSection 
 setter for sectionName - sets The name of this document section, e.g.
   setSectionName(int, String)   - 
Method in class edu.uchsc.ccp.uima.annotation. CCPDocumentSection_Type 
  
   setSectionName(String)   - 
Method in class edu.uchsc.ccp.uima.annotation. CCPDocumentSubSection 
 setter for sectionName - sets The name of the document subsection.
   setSectionName(int, String)   - 
Method in class edu.uchsc.ccp.uima.annotation. CCPDocumentSubSection_Type 
  
   setSectionStartIndex(int)   - 
Method in class edu.uchsc.ccp.util.nlp.document. DocumentSection 
  
   setSlotMentions(FSArray)   - 
Method in class edu.uchsc.ccp.uima.mention. CCPClassMention 
 setter for slotMentions - sets A class mention optionally has slot mentions which represent attributes of that class.
   setSlotMentions(int, CCPSlotMention)   - 
Method in class edu.uchsc.ccp.uima.mention. CCPClassMention 
 indexed setter for slotMentions - sets an indexed value - A class mention optionally has slot mentions which represent attributes of that class.
   setSlotMentions(int, int)   - 
Method in class edu.uchsc.ccp.uima.mention. CCPClassMention_Type 
  
   setSlotMentions(int, int, int)   - 
Method in class edu.uchsc.ccp.uima.mention. CCPClassMention_Type 
  
   setSlotMentions(List<SlotMention>)   - 
Method in class edu.uchsc.ccp.util.nlp.mention. ClassMention 
  
   setSlotValues(StringArray)   - 
Method in class edu.uchsc.ccp.uima.mention. CCPNonComplexSlotMention 
 setter for slotValues - sets Slot fillers (values) for this non-complex slot mention.
   setSlotValues(int, String)   - 
Method in class edu.uchsc.ccp.uima.mention. CCPNonComplexSlotMention 
 indexed setter for slotValues - sets an indexed value - Slot fillers (values) for this non-complex slot mention.
   setSlotValues(int, int)   - 
Method in class edu.uchsc.ccp.uima.mention. CCPNonComplexSlotMention_Type 
  
   setSlotValues(int, int, String)   - 
Method in class edu.uchsc.ccp.uima.mention. CCPNonComplexSlotMention_Type 
  
   setSlotValues(List<Object>)   - 
Method in class edu.uchsc.ccp.util.nlp.mention. SlotMention 
 Set the slot values
   setSpan(Span)   - 
Method in class edu.uchsc.ccp.util.nlp.annotation. TextAnnotation 
 Set the annotation span to be equal to the single input span.
   setSpanEnd(int)   - 
Method in class edu.uchsc.ccp.uima.annotation. CCPSpan 
 setter for spanEnd - sets The character offset for the end of the span.
   setSpanEnd(int, int)   - 
Method in class edu.uchsc.ccp.uima.annotation. CCPSpan_Type 
  
   setSpanEnd(int)   - 
Method in class edu.uchsc.ccp.util.nlp.annotation. Span 
  
   setSpans(FSArray)   - 
Method in class edu.uchsc.ccp.uima.annotation. CCPTextAnnotation 
 setter for spans - sets This FSArray stores the CCPSpans which comprise this annotation.
   setSpans(int, CCPSpan)   - 
Method in class edu.uchsc.ccp.uima.annotation. CCPTextAnnotation 
 indexed setter for spans - sets an indexed value - This FSArray stores the CCPSpans which comprise this annotation.
   setSpans(int, int)   - 
Method in class edu.uchsc.ccp.uima.annotation. CCPTextAnnotation_Type 
  
   setSpans(int, int, int)   - 
Method in class edu.uchsc.ccp.uima.annotation. CCPTextAnnotation_Type 
  
   setSpans(List<Span>)   - 
Method in class edu.uchsc.ccp.util.nlp.annotation. TextAnnotation 
 Set the span list equal to the inputted span list
   setSpanStart(int)   - 
Method in class edu.uchsc.ccp.uima.annotation. CCPSpan 
 setter for spanStart - sets The character offset for the start of the span.
   setSpanStart(int, int)   - 
Method in class edu.uchsc.ccp.uima.annotation. CCPSpan_Type 
  
   setSpanStart(int)   - 
Method in class edu.uchsc.ccp.util.nlp.annotation. Span 
  
   setStem(String)   - 
Method in class edu.uchsc.ccp.uima.annotation.syntactic. CCPTokenAnnotation 
 setter for stem - sets The token stem.
   setStem(int, String)   - 
Method in class edu.uchsc.ccp.uima.annotation.syntactic. CCPTokenAnnotation_Type 
  
   setSubSections(FSArray)   - 
Method in class edu.uchsc.ccp.uima.annotation. CCPDocumentSection 
 setter for subSections - sets Allows for direct linking to CCPDocumentSubSections residing in this document section.
   setSubSections(int, CCPDocumentSubSection)   - 
Method in class edu.uchsc.ccp.uima.annotation. CCPDocumentSection 
 indexed setter for subSections - sets an indexed value - Allows for direct linking to CCPDocumentSubSections residing in this document section.
   setSubSections(int, int)   - 
Method in class edu.uchsc.ccp.uima.annotation. CCPDocumentSection_Type 
  
   setSubSections(int, int, int)   - 
Method in class edu.uchsc.ccp.uima.annotation. CCPDocumentSection_Type 
  
   setTextAnnotation(List<TextAnnotation>)   - 
Method in class edu.uchsc.ccp.util.nlp.mention. ClassMention 
  
   setTitle(String)   - 
Method in class edu.uchsc.ccp.util.nlp.stats. PRFResult 
  
   setTitleText(String)   - 
Method in class edu.uchsc.ccp.util.nlp.document. GenericDocument 
  Deprecated.  
   setTokenNumber(int)   - 
Method in class edu.uchsc.ccp.uima.annotation.syntactic. CCPTokenAnnotation 
 setter for tokenNumber - sets The token number indicates the placement of the token within the document text.
   setTokenNumber(int, int)   - 
Method in class edu.uchsc.ccp.uima.annotation.syntactic. CCPTokenAnnotation_Type 
  
   setTPAnnotations(List<TextAnnotation>)   - 
Method in class edu.uchsc.ccp.util.nlp.stats. PRFResult 
 Set the true positive  TextAnnotation  list
   setTruePositiveCount(int)   - 
Method in class edu.uchsc.ccp.util.nlp.stats. PRFResult 
  
   setTypedDependencies(FSArray)   - 
Method in class edu.uchsc.ccp.uima.annotation.syntactic. CCPTokenAnnotation 
 setter for typedDependencies - sets If this token is known to be involved in any typed dependency structures, then they are linked through this FSArray.
   setTypedDependencies(int, CCPTypedDependency)   - 
Method in class edu.uchsc.ccp.uima.annotation.syntactic. CCPTokenAnnotation 
 indexed setter for typedDependencies - sets an indexed value - If this token is known to be involved in any typed dependency structures, then they are linked through this FSArray.
   setTypedDependencies(int, int)   - 
Method in class edu.uchsc.ccp.uima.annotation.syntactic. CCPTokenAnnotation_Type 
  
   setTypedDependencies(int, int, int)   - 
Method in class edu.uchsc.ccp.uima.annotation.syntactic. CCPTokenAnnotation_Type 
  
   setUseOneLineFormatStatus(boolean)   - 
Method in class edu.uchsc.ccp.util.nlp.annotation.printer. DefaultPrintOutput 
  
   setUseOneLineFormatStatus(boolean)   - 
Method in class edu.uchsc.ccp.util.nlp.annotation.printer. SimpleCollectionReaderFormatOutput 
  
   setValue(int)   - 
Method in class edu.uchsc.ccp.util.nlp. KeyValuePair 
 Sets the value
   SharedEndSpanComparator   - Class in  edu.uchsc.ccp.util.nlp.annotation.comparison  For "shared end" comparison, the spans are required to have the same end index in order to return 0.   SharedEndSpanComparator()   - 
Constructor for class edu.uchsc.ccp.util.nlp.annotation.comparison. SharedEndSpanComparator 
  
   SharedStartOrEndSpanComparator   - Class in  edu.uchsc.ccp.util.nlp.annotation.comparison  For "shared start or end" comparison, the spans are required to have the same start or end indexes in order to return 0.   SharedStartOrEndSpanComparator()   - 
Constructor for class edu.uchsc.ccp.util.nlp.annotation.comparison. SharedStartOrEndSpanComparator 
  
   SharedStartSpanComparator   - Class in  edu.uchsc.ccp.util.nlp.annotation.comparison  For "shared start" comparison, the spans are required to have the same starting index in order to return 0.   SharedStartSpanComparator()   - 
Constructor for class edu.uchsc.ccp.util.nlp.annotation.comparison. SharedStartSpanComparator 
  
   sharesSpanEnd(Span, Span)   - 
Method in class edu.uchsc.ccp.util.nlp.annotation.comparison. SpanComparator 
 Returns 0 if span1 and span2 share the same end index.
   sharesSpanEnd(List<Span>, List<Span>)   - 
Method in class edu.uchsc.ccp.util.nlp.annotation.comparison. SpanComparator 
 Returns 0 if span1 and span2 share the same end index.
   sharesSpanStart(Span, Span)   - 
Method in class edu.uchsc.ccp.util.nlp.annotation.comparison. SpanComparator 
 Returns 0 if span1 and span2 share the same start index.
   sharesSpanStart(List<Span>, List<Span>)   - 
Method in class edu.uchsc.ccp.util.nlp.annotation.comparison. SpanComparator 
 Returns 0 if span1 and span2 share the same start index.
   sharesSpanStartOrEnd(Span, Span)   - 
Method in class edu.uchsc.ccp.util.nlp.annotation.comparison. SpanComparator 
 Returns 0 if span1 and span2 share the same start or end index (or both).
   sharesSpanStartOrEnd(List<Span>, List<Span>)   - 
Method in class edu.uchsc.ccp.util.nlp.annotation.comparison. SpanComparator 
 Returns 0 if span1 and span2 share the same start or end index (or both).
   SimpleCollectionReaderFormatOutput   - Class in  edu.uchsc.ccp.util.nlp.annotation.printer  THis class outputs the document text and documentid into a format that can be read in by the SimpleCollectionReader, namely documentID|documentText   SimpleCollectionReaderFormatOutput()   - 
Constructor for class edu.uchsc.ccp.util.nlp.annotation.printer. SimpleCollectionReaderFormatOutput 
  
   SloppySpanComparator   - Class in  edu.uchsc.ccp.util.nlp.annotation.comparison  For "sloppy" comparison, the spans are required to overlap by at least one character.   SloppySpanComparator()   - 
Constructor for class edu.uchsc.ccp.util.nlp.annotation.comparison. SloppySpanComparator 
  
   SLOT_MENTION_TYPE   - 
Static variable in interface edu.uchsc.ccp.util.nlp.mention. SlotMentionTypes 
 SlotMention Identifier - if this changes, Annotation DB must be adjusted accordingly
   SlotMention   - Class in  edu.uchsc.ccp.util.nlp.mention      SlotMention()   - 
Constructor for class edu.uchsc.ccp.util.nlp.mention. SlotMention 
  Deprecated.  
   SlotMention(String)   - 
Constructor for class edu.uchsc.ccp.util.nlp.mention. SlotMention 
  
   SlotMentionComparator   - Interface in  edu.uchsc.ccp.util.nlp.mention.comparison      SlotMentionTypes   - Interface in  edu.uchsc.ccp.util.nlp.mention      SlotRemovalFilter_AE   - Class in  edu.uchsc.ccp.uima.ae.util.filter  removes slots from a specific class mention.   SlotRemovalFilter_AE()   - 
Constructor for class edu.uchsc.ccp.uima.ae.util.filter. SlotRemovalFilter_AE 
  
   sortHashMapByValues(Map<String, Integer>)   - 
Static method in class edu.uchsc.ccp.util.nlp. GenericTextUtils 
 Sorts a Hashmap by the values contained in the HashMap
   Span   - Class in  edu.uchsc.ccp.util.nlp.annotation      Span(int, int)   - 
Constructor for class edu.uchsc.ccp.util.nlp.annotation. Span 
  
   SpanComparator   - Class in  edu.uchsc.ccp.util.nlp.annotation.comparison      SpanComparator()   - 
Constructor for class edu.uchsc.ccp.util.nlp.annotation.comparison. SpanComparator 
  
   StanfordParser_AE   - Class in  edu.uchsc.ccp.uima.ae.nlp.parser  This class incorporates the StanfordParser into the UIMA analysis engine framework via the StanfordParserUtil.   StanfordParser_AE()   - 
Constructor for class edu.uchsc.ccp.uima.ae.nlp.parser. StanfordParser_AE 
  
   StanfordParser_Util   - Class in  edu.uchsc.ccp.util.external.stanfordparser  This class converts the output of the StanfordParser into a distinct text annotation objects.   StanfordParser_Util(String)   - 
Constructor for class edu.uchsc.ccp.util.external.stanfordparser. StanfordParser_Util 
 Construct a new StanfordParserUtil.
   StanfordParser_Util(String, boolean)   - 
Constructor for class edu.uchsc.ccp.util.external.stanfordparser. StanfordParser_Util 
  
   StanfordParser_Util(String, String)   - 
Constructor for class edu.uchsc.ccp.util.external.stanfordparser. StanfordParser_Util 
 Construct a new StanfordParserUtil while selecting the output tree display format.
   StanfordParser_Util(String, String, boolean)   - 
Constructor for class edu.uchsc.ccp.util.external.stanfordparser. StanfordParser_Util 
  
   startsBefore(Span)   - 
Method in class edu.uchsc.ccp.util.nlp.annotation. Span 
 Returns true if this span starts before the inputted span, false otherwise.
   stem(String)   - 
Method in class edu.uchsc.ccp.util.nlp.stemmer. PorterStemmer_Util 
 Stems the input text
   STOPWORDS   - 
Static variable in class edu.uchsc.ccp.util.nlp. GenericTextUtils 
  
   StrictSpanComparator   - Class in  edu.uchsc.ccp.util.nlp.annotation.comparison  For "strict" comparison, the spans are required to be exactly the same in order to return 0.   StrictSpanComparator()   - 
Constructor for class edu.uchsc.ccp.util.nlp.annotation.comparison. StrictSpanComparator 
  
   SUBCELLULAR_LOCATION   - 
Static variable in interface edu.uchsc.ccp.util.nlp.mention. ClassMentionTypes 
  
   SUBSTITUTION   - 
Static variable in interface edu.uchsc.ccp.util.nlp.mention. ClassMentionTypes 
  
   SUBSTITUTION_MUTANT_ELEMENT   - 
Static variable in interface edu.uchsc.ccp.util.nlp.mention. ComplexSlotMentionTypes 
  
   SUBSTITUTION_WILDTYPE_ELEMENT   - 
Static variable in interface edu.uchsc.ccp.util.nlp.mention. ComplexSlotMentionTypes 
  
   summarizeJCas(String, JCas)   - 
Method in class edu.uchsc.ccp.uima.util.opendmap. TestSummarizer 
 Generate summary strings for all annotations in the JCAS that are described in the
 pattern string for this summarizer.
   swapAnnotationInfo(CCPTextAnnotation, TextAnnotation, JCas)   - 
Static method in class edu.uchsc.ccp.uima.util. UIMA_Util 
 This method transfers general annotation info, i.e.
   swapAnnotationInfo(CCPTextAnnotation, CCPTextAnnotation)   - 
Static method in class edu.uchsc.ccp.uima.util. UIMA_Util 
 Swap from UIMA Annotation to another UIMA Annotation
   swapAnnotationInfo(TextAnnotation, CCPTextAnnotation, JCas)   - 
Static method in class edu.uchsc.ccp.uima.util. UIMA_Util 
 This method transfers general annotation info, i.e.
   swapAnnotationInfo(TextAnnotation, TextAnnotation)   - 
Static method in class edu.uchsc.ccp.util.nlp.annotation. Annotation_Util 
  
   swapAnnotationSetInfo(CCPAnnotationSet, AnnotationSet)   - 
Static method in class edu.uchsc.ccp.uima.util. UIMA_Util 
  
   swapAnnotationSetInfo(AnnotationSet, CCPAnnotationSet)   - 
Static method in class edu.uchsc.ccp.uima.util. UIMA_Util 
  
   swapAnnotatorInfo(CCPAnnotator, Annotator)   - 
Static method in class edu.uchsc.ccp.uima.util. UIMA_Util 
  
   swapAnnotatorInfo(Annotator, CCPAnnotator)   - 
Static method in class edu.uchsc.ccp.uima.util. UIMA_Util 
  
   swapClassMentionInfo(CCPClassMention, CCPClassMention)   - 
Static method in class edu.uchsc.ccp.uima.util. UIMA_Util 
  
   swapComplexSlotMentionInfo(CCPComplexSlotMention, CCPComplexSlotMention)   - 
Static method in class edu.uchsc.ccp.uima.util. UIMA_Util 
  
   swapDocumentInfo(JCas, GenericDocument)   - 
Static method in class edu.uchsc.ccp.uima.util. UIMA_Util 
  
   swapNonComplexSlotMentionInfo(CCPNonComplexSlotMention, CCPNonComplexSlotMention)   - 
Static method in class edu.uchsc.ccp.uima.util. UIMA_Util 
  
 
 
    
 T  
 
   TABLE_ID   - 
Static variable in interface edu.uchsc.ccp.util.nlp.mention. SlotMentionTypes 
  
   TAGGER_TYPE_GENE   - 
Static variable in class edu.uchsc.ccp.util.external.penn. PennBioTaggerEISystem 
  
   TAGGER_TYPE_MALIGNANCY   - 
Static variable in class edu.uchsc.ccp.util.external.penn. PennBioTaggerEISystem 
  
   TAGGER_TYPE_VARIATION   - 
Static variable in class edu.uchsc.ccp.util.external.penn. PennBioTaggerEISystem 
  
   TAXON   - 
Static variable in interface edu.uchsc.ccp.util.nlp.mention. ClassMentionTypes 
 For Species Classification
   TestPrinter   - Class in  edu.uchsc.ccp.uima.cc.summary.opendmap  This class looks through the JCas for annotations of a certain type
 and prints them in a serialized format appropriate for later comparison
 using the TestChecker.   TestPrinter()   - 
Constructor for class edu.uchsc.ccp.uima.cc.summary.opendmap. TestPrinter 
  
   TestSummarizer   - Class in  edu.uchsc.ccp.uima.util.opendmap  This class prints out selected contents of the CAS in the standard
 annotation summery format.   TestSummarizer(String)   - 
Constructor for class edu.uchsc.ccp.uima.util.opendmap. TestSummarizer 
 Create a new TestSummarizer that will generate summary strings for the mentions and slots
 specified in the pattern.
   TexasCorpusParser   - Class in  edu.uchsc.ccp.util.nlp.parser.corpus  A parser for the Texas corpus.   TexasCorpusParser(String)   - 
Constructor for class edu.uchsc.ccp.util.nlp.parser.corpus. TexasCorpusParser 
 Initialize a new parser for the texas corpus.
   TextAnnotation   - Class in  edu.uchsc.ccp.util.nlp.annotation  This class is a generalized representation of a text annotation.   TextAnnotation(int, int, String, Annotator, AnnotationSet, int, int, String, int, ClassMention)   - 
Constructor for class edu.uchsc.ccp.util.nlp.annotation. TextAnnotation 
  
   TextAnnotation()   - 
Constructor for class edu.uchsc.ccp.util.nlp.annotation. TextAnnotation 
  Deprecated.  
   THREONINE   - 
Static variable in interface edu.uchsc.ccp.util.nlp.mention. ClassMentionTypes 
  
   TISSUE   - 
Static variable in interface edu.uchsc.ccp.util.nlp.mention. ClassMentionTypes 
  
   TITLE_ID   - 
Static variable in interface edu.uchsc.ccp.util.nlp.document. DocumentSectionTypes 
  
   TITLE_NAME   - 
Static variable in interface edu.uchsc.ccp.util.nlp.document. DocumentSectionTypes 
  
   TOKEN   - 
Static variable in interface edu.uchsc.ccp.util.nlp.mention. ClassMentionTypes 
 Syntactic Class Mentions
   TOKEN_IS_ANAPHOR_FOR   - 
Static variable in interface edu.uchsc.ccp.util.nlp.mention. SlotMentionTypes 
  
   TOKEN_LEMMA   - 
Static variable in interface edu.uchsc.ccp.util.nlp.mention. SlotMentionTypes 
  
   TOKEN_NUMBER   - 
Static variable in interface edu.uchsc.ccp.util.nlp.mention. SlotMentionTypes 
  
   TOKEN_PARTOFSPEECH   - 
Static variable in interface edu.uchsc.ccp.util.nlp.mention. SlotMentionTypes 
 Syntactic Slot Mentions
   TOKEN_STEM   - 
Static variable in interface edu.uchsc.ccp.util.nlp.mention. SlotMentionTypes 
  
   TOKEN_TYPED_DEPENDENCY   - 
Static variable in interface edu.uchsc.ccp.util.nlp.mention. SlotMentionTypes 
  
   tokenizeIntoSentences(String, String, String)   - 
Static method in class edu.uchsc.ccp.util.nlp. GenericTextUtils 
 This method tokenizes the input text into sentences via the KeX sentence tokenizing utility. 
  
 It should be noted that sometimes the KeX sentence tokenizer modifies the output text (usually by adding or deleting a space) so that it is not
 possible to determine the offset in the original text.
   Tokenizer   - Interface in  edu.uchsc.ccp.util.nlp.tokenizer  This class is an interface for tokenizers   toString()   - 
Method in class edu.uchsc.ccp.uima.cc.summary.comparison. AnnotationGroupID 
  
   toString()   - 
Method in class edu.uchsc.ccp.uima.util.opendmap. AnnotationString 
 Return the standard format string for this annotation.
   toString()   - 
Method in class edu.uchsc.ccp.util.external.stanfordparser. TypedDependencyCls 
  
   toString()   - 
Method in class edu.uchsc.ccp.util.nlp.annotation. Span 
  
   trainGENIATagger(String, String)   - 
Static method in class edu.uchsc.ccp.util.external.lingpipe. LingPipe_Util 
  
   trainMedPostTagger(String, String)   - 
Static method in class edu.uchsc.ccp.util.external.lingpipe. LingPipe_Util 
 Much of this code was taken from: http://alias-i.com/lingpipe/demos/tutorial/posTags/src/TrainMedPost.java
   TRANSPORT_GATED   - 
Static variable in interface edu.uchsc.ccp.util.nlp.mention. ClassMentionTypes 
  
   trimCCPTextAnnotation(CCPTextAnnotation)   - 
Static method in class edu.uchsc.ccp.uima.util. UIMA_Annotation_Util 
 trims spaces and   from ends of a CCPTextAnnotation
   TRYPTOPHAN   - 
Static variable in interface edu.uchsc.ccp.util.nlp.mention. ClassMentionTypes 
  
   type   - 
Static variable in class edu.uchsc.ccp.uima.annotation. CCPAnnotation 
  
   type   - 
Static variable in class edu.uchsc.ccp.uima.annotation. CCPAnnotationSet 
  
   type   - 
Static variable in class edu.uchsc.ccp.uima.annotation. CCPAnnotator 
  
   type   - 
Static variable in class edu.uchsc.ccp.uima.annotation. CCPDocumentAnnotation 
  
   type   - 
Static variable in class edu.uchsc.ccp.uima.annotation. CCPDocumentInformation 
  
   type   - 
Static variable in class edu.uchsc.ccp.uima.annotation. CCPDocumentSection 
  
   type   - 
Static variable in class edu.uchsc.ccp.uima.annotation. CCPDocumentSubSection 
  
   type   - 
Static variable in class edu.uchsc.ccp.uima.annotation. CCPSemanticAnnotation 
  
   type   - 
Static variable in class edu.uchsc.ccp.uima.annotation. CCPSpan 
  
   type   - 
Static variable in class edu.uchsc.ccp.uima.annotation. CCPSyntacticAnnotation 
  
   type   - 
Static variable in class edu.uchsc.ccp.uima.annotation. CCPTextAnnotation 
  
   type   - 
Static variable in class edu.uchsc.ccp.uima.annotation.syntactic. CCPClauseAnnotation 
  
   type   - 
Static variable in class edu.uchsc.ccp.uima.annotation.syntactic. CCPPhraseAnnotation 
  
   type   - 
Static variable in class edu.uchsc.ccp.uima.annotation.syntactic. CCPSentenceAnnotation 
  
   type   - 
Static variable in class edu.uchsc.ccp.uima.annotation.syntactic. CCPTokenAnnotation 
  
   type   - 
Static variable in class edu.uchsc.ccp.uima.annotation.syntactic. CCPTypedDependency 
  
   type   - 
Static variable in class edu.uchsc.ccp.uima.mention. CCPClassMention 
  
   type   - 
Static variable in class edu.uchsc.ccp.uima.mention. CCPComplexSlotMention 
  
   type   - 
Static variable in class edu.uchsc.ccp.uima.mention. CCPMention 
  
   type   - 
Static variable in class edu.uchsc.ccp.uima.mention. CCPNonComplexSlotMention 
  
   type   - 
Static variable in class edu.uchsc.ccp.uima.mention. CCPSlotMention 
  
   TypedDependencyCls   - Class in  edu.uchsc.ccp.util.external.stanfordparser      TypedDependencyCls(int, int, String)   - 
Constructor for class edu.uchsc.ccp.util.external.stanfordparser. TypedDependencyCls 
  
   TypedDependencyCls_Util   - Class in  edu.uchsc.ccp.util.external.stanfordparser      TypedDependencyCls_Util()   - 
Constructor for class edu.uchsc.ccp.util.external.stanfordparser. TypedDependencyCls_Util 
  
   typeIndexID   - 
Static variable in class edu.uchsc.ccp.uima.annotation. CCPAnnotation 
  
   typeIndexID   - 
Static variable in class edu.uchsc.ccp.uima.annotation. CCPAnnotation_Type 
  
   typeIndexID   - 
Static variable in class edu.uchsc.ccp.uima.annotation. CCPAnnotationSet 
  
   typeIndexID   - 
Static variable in class edu.uchsc.ccp.uima.annotation. CCPAnnotationSet_Type 
  
   typeIndexID   - 
Static variable in class edu.uchsc.ccp.uima.annotation. CCPAnnotator 
  
   typeIndexID   - 
Static variable in class edu.uchsc.ccp.uima.annotation. CCPAnnotator_Type 
  
   typeIndexID   - 
Static variable in class edu.uchsc.ccp.uima.annotation. CCPDocumentAnnotation 
  
   typeIndexID   - 
Static variable in class edu.uchsc.ccp.uima.annotation. CCPDocumentAnnotation_Type 
  
   typeIndexID   - 
Static variable in class edu.uchsc.ccp.uima.annotation. CCPDocumentInformation 
  
   typeIndexID   - 
Static variable in class edu.uchsc.ccp.uima.annotation. CCPDocumentInformation_Type 
  
   typeIndexID   - 
Static variable in class edu.uchsc.ccp.uima.annotation. CCPDocumentSection 
  
   typeIndexID   - 
Static variable in class edu.uchsc.ccp.uima.annotation. CCPDocumentSection_Type 
  
   typeIndexID   - 
Static variable in class edu.uchsc.ccp.uima.annotation. CCPDocumentSubSection 
  
   typeIndexID   - 
Static variable in class edu.uchsc.ccp.uima.annotation. CCPDocumentSubSection_Type 
  
   typeIndexID   - 
Static variable in class edu.uchsc.ccp.uima.annotation. CCPSemanticAnnotation 
  
   typeIndexID   - 
Static variable in class edu.uchsc.ccp.uima.annotation. CCPSemanticAnnotation_Type 
  
   typeIndexID   - 
Static variable in class edu.uchsc.ccp.uima.annotation. CCPSpan 
  
   typeIndexID   - 
Static variable in class edu.uchsc.ccp.uima.annotation. CCPSpan_Type 
  
   typeIndexID   - 
Static variable in class edu.uchsc.ccp.uima.annotation. CCPSyntacticAnnotation 
  
   typeIndexID   - 
Static variable in class edu.uchsc.ccp.uima.annotation. CCPSyntacticAnnotation_Type 
  
   typeIndexID   - 
Static variable in class edu.uchsc.ccp.uima.annotation. CCPTextAnnotation 
  
   typeIndexID   - 
Static variable in class edu.uchsc.ccp.uima.annotation. CCPTextAnnotation_Type 
  
   typeIndexID   - 
Static variable in class edu.uchsc.ccp.uima.annotation.syntactic. CCPClauseAnnotation 
  
   typeIndexID   - 
Static variable in class edu.uchsc.ccp.uima.annotation.syntactic. CCPClauseAnnotation_Type 
  
   typeIndexID   - 
Static variable in class edu.uchsc.ccp.uima.annotation.syntactic. CCPPhraseAnnotation 
  
   typeIndexID   - 
Static variable in class edu.uchsc.ccp.uima.annotation.syntactic. CCPPhraseAnnotation_Type 
  
   typeIndexID   - 
Static variable in class edu.uchsc.ccp.uima.annotation.syntactic. CCPSentenceAnnotation 
  
   typeIndexID   - 
Static variable in class edu.uchsc.ccp.uima.annotation.syntactic. CCPSentenceAnnotation_Type 
  
   typeIndexID   - 
Static variable in class edu.uchsc.ccp.uima.annotation.syntactic. CCPTokenAnnotation 
  
   typeIndexID   - 
Static variable in class edu.uchsc.ccp.uima.annotation.syntactic. CCPTokenAnnotation_Type 
  
   typeIndexID   - 
Static variable in class edu.uchsc.ccp.uima.annotation.syntactic. CCPTypedDependency 
  
   typeIndexID   - 
Static variable in class edu.uchsc.ccp.uima.annotation.syntactic. CCPTypedDependency_Type 
  
   typeIndexID   - 
Static variable in class edu.uchsc.ccp.uima.mention. CCPClassMention 
  
   typeIndexID   - 
Static variable in class edu.uchsc.ccp.uima.mention. CCPClassMention_Type 
  
   typeIndexID   - 
Static variable in class edu.uchsc.ccp.uima.mention. CCPComplexSlotMention 
  
   typeIndexID   - 
Static variable in class edu.uchsc.ccp.uima.mention. CCPComplexSlotMention_Type 
  
   typeIndexID   - 
Static variable in class edu.uchsc.ccp.uima.mention. CCPMention 
  
   typeIndexID   - 
Static variable in class edu.uchsc.ccp.uima.mention. CCPMention_Type 
  
   typeIndexID   - 
Static variable in class edu.uchsc.ccp.uima.mention. CCPNonComplexSlotMention 
  
   typeIndexID   - 
Static variable in class edu.uchsc.ccp.uima.mention. CCPNonComplexSlotMention_Type 
  
   typeIndexID   - 
Static variable in class edu.uchsc.ccp.uima.mention. CCPSlotMention 
  
   typeIndexID   - 
Static variable in class edu.uchsc.ccp.uima.mention. CCPSlotMention_Type 
  
   TYROSINE   - 
Static variable in interface edu.uchsc.ccp.util.nlp.mention. ClassMentionTypes 
  
 
 
    
 U  
 
   UIMA_Annotation_Util   - Class in  edu.uchsc.ccp.uima.util      UIMA_Annotation_Util()   - 
Constructor for class edu.uchsc.ccp.uima.util. UIMA_Annotation_Util 
  
   UIMA_Util   - Class in  edu.uchsc.ccp.uima.util  This is a utility class for converting between UIMA annotations and TextAnnotations.   UIMA_Util()   - 
Constructor for class edu.uchsc.ccp.uima.util. UIMA_Util 
  
   UIMASyntacticAnnotation_Util   - Class in  edu.uchsc.ccp.uima.util  This class has been constructed to automate the conversion of annotations with class mention types of "token", "phrase", "clause", "sentence" into
 TokenAnnotations, PhraseAnnotations, ClauseAnnotations, and SentenceAnnotations, respectively.   UIMASyntacticAnnotation_Util()   - 
Constructor for class edu.uchsc.ccp.uima.util. UIMASyntacticAnnotation_Util 
  
   UNDEFINED   - 
Static variable in interface edu.uchsc.ccp.util.nlp.mention. ClassMentionTypes 
 For MedLEE
 
 
    
 V  
 
   validateCCPClassMention(CCPClassMention)   - 
Static method in class edu.uchsc.ccp.uima.util. UIMA_Util 
 This method checks each slot mention associated with the input class mention for valid slot fillers.
   validateCCPComplexSlotMention(CCPComplexSlotMention)   - 
Static method in class edu.uchsc.ccp.uima.util. UIMA_Util 
 This method checks for valid slot fillers of the input CCPComplexSlotMention, i.e.
   validateCCPNonComplexSlotMention(CCPNonComplexSlotMention)   - 
Static method in class edu.uchsc.ccp.uima.util. UIMA_Util 
 This method checks for valid slot fillers of the input CCPNonComplexSlotMention, i.e.
   validateCCPSlotMention(CCPSlotMention)   - 
Static method in class edu.uchsc.ccp.uima.util. UIMA_Util 
 This method determines the type of slot mention inputted, and calls either validateCCPComplexSlotMention() or
 validateCCPNonComplexSlotMention()
   validateCCPTextAnnotation(CCPTextAnnotation)   - 
Static method in class edu.uchsc.ccp.uima.util. UIMA_Util 
 This method provides a sanity check when constructing CCPTextAnnotations, and in particular the class mention structure from scratch.
   validateXML(String)   - 
Static method in class edu.uchsc.ccp.util.xml. XMLValidityChecker 
 Validates the input XML file.
   VALINE   - 
Static variable in interface edu.uchsc.ccp.util.nlp.mention. ClassMentionTypes 
  
   VARIATION_EVENT   - 
Static variable in interface edu.uchsc.ccp.util.nlp.mention. ClassMentionTypes 
  
   VARIATION_TYPE   - 
Static variable in interface edu.uchsc.ccp.util.nlp.mention. ClassMentionTypes 
  
   VIRUS   - 
Static variable in interface edu.uchsc.ccp.util.nlp.mention. ClassMentionTypes 
  
 
 
    
 W  
 
   warn(String)   - 
Static method in class edu.uchsc.ccp.util.external.lingpipe. LingPipe_Util 
  
   warn(String)   - 
Method in class edu.uchsc.ccp.util.nlp.parser.corpus. CorpusParser 
  
 
 
    
 X  
 
   XMLEntityExtractor   - Class in  edu.uchsc.ccp.util.nlp.ei.xml      XMLEntityExtractor()   - 
Constructor for class edu.uchsc.ccp.util.nlp.ei.xml. XMLEntityExtractor 
  
   XMLValidityChecker   - Class in  edu.uchsc.ccp.util.xml  This class utilizes the XMLReader class to validate XML.   XMLValidityChecker()   - 
Constructor for class edu.uchsc.ccp.util.xml. XMLValidityChecker 
  
 
 
    
 Y  
 
   YapexCorpusParser   - Class in  edu.uchsc.ccp.util.nlp.parser.corpus  This class is used to parse out the gold standard annotations from the Yapex corpus. 
  
 Output Classes: 
 
 protein
    YapexCorpusParser(String, boolean)   - 
Constructor for class edu.uchsc.ccp.util.nlp.parser.corpus. YapexCorpusParser 
 The constructor for the iterator-like corpus parser.
 
 
 A   B   C   D   E   F   G   H   I   K   L   M   N   O   P   R   S   T   U   V   W   X   Y  

 
   
  
 
 
 
   
 
   
          Overview     
        Package   
        Class   
          Tree     
          Deprecated     
       Index    
          Help     
   
 
 
  
 
 
 

 
  
 PREV 
 NEXT  
  
    FRAMES     
   NO FRAMES     
  
    All Classes  ');
  }
  //-->
 
 
    All Classes
